# NI-BODIPY-GO Nanocomposites for Targeted PDT

**DOI:** 10.1021/acsomega.2c06900

**Published:** 2023-02-22

**Authors:** Ezel Öztürk Gündüz, Berkan Tasasız, M. Emre Gedik, Gürcan Günaydın, Elif Okutan

**Affiliations:** † Department of Chemistry, Faculty of Science, Gebze Technical University, Gebze, Kocaeli 41400, Turkey; ‡ Department of Basic Oncology, Cancer Institute, Hacettepe University, Çankaya, Ankara 06800, Turkey

## Abstract

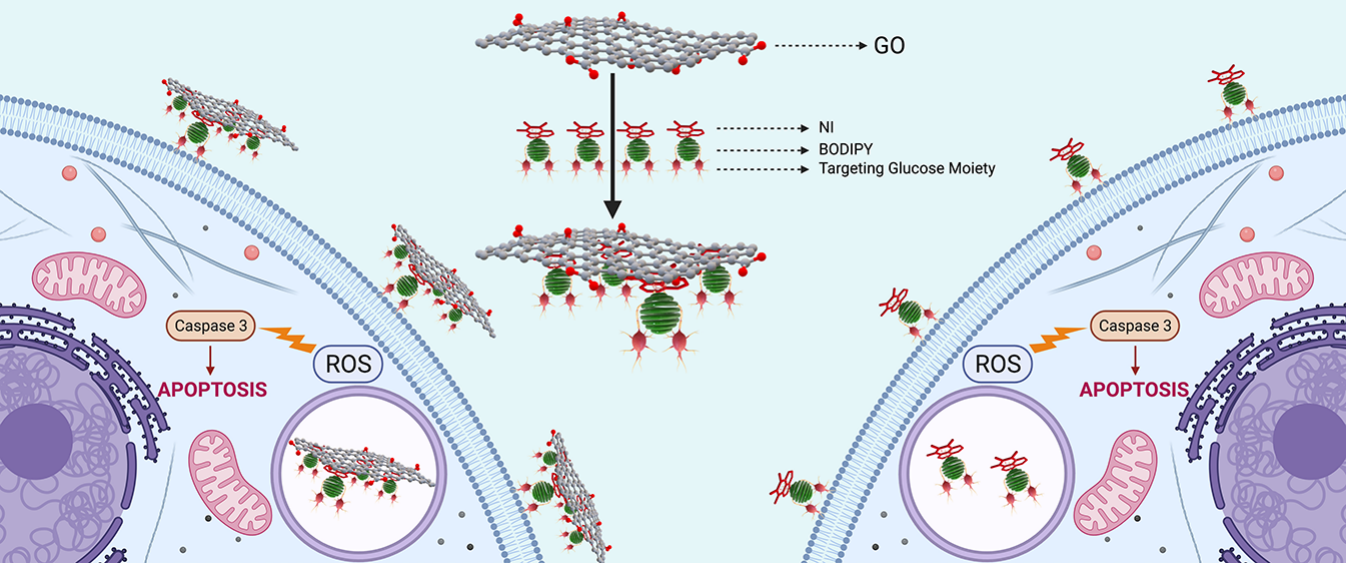

Three multifunctional targeted NI-BODIPYs (**10**–**12**) and **GO**-(**10**–**12**) nanocarriers were fabricated. NI-BODIPYs are designed to facilitate non-covalent interaction with graphene oxide (GO) and target toward cancer cells for specific recognition with glucose moieties while efficiently producing singlet oxygen. We probed detailed characterization, fundamental photophysical/photochemical properties, and interactions with GO of such triplet photosensitizers and nanocarriers. The effect of the formation of nanohybrids with GO on singlet oxygen formation as well as on the efficacies of the molecules in terms of *in vitro* killing of cancer cells was evaluated with K562 human chronic myelogenous leukemia cells. Amazingly, it was observed that GO exhibited favorable interactions with the NI-BODIPY dyads and promoted the formation of singlet oxygen, while not showing any dark toxicity.

## Introduction

Photodynamic therapy (PDT), which acts via irradiation of photosensitizers (PSs) with an appropriate wavelength of light that carry energy to surrounding molecular oxygen (^3^O_2_) for the generation of reactive oxygen species (ROS) such as singlet oxygen (^1^O_2_), has been comprehensively studied as a non-invasive therapeutic strategy for various types of cancer.^1−3^ There are several advantages of PDT over conventional remedies, such as localized high efficiency and tumor peculiar treatment without any accumulating toxicity. However, some drawbacks certainly hinder the clinical applications of PDT.^4^ Numerous convenient PSs lack the property of hydrophilic/hydrophobic balance, which led to aggregation or biocompatibility problems, thus constituting challenges for transportation in biological environments.^5−7^ Since the locally generated lifetime (0.6 × 10^–6^ s) and diffusion range of ^1^O_2_ are both very short, it is crucial to design the PSs for high ^1^O_2_ generation efficiency and selectivity with targeting sites.^8−10^ It is, therefore, substantial to shape advanced delivery systems to address the aforementioned issues. Nanomaterials such as gold, silica, and carbon nanotubes have been studied as carriers, and several of them have been reported to bear high aqueous dispersibility, bioavailability, and having suitable dimensions for tumor uptake.^11−13^ In order to achieve higher uptake and reduced side effects, these nanomaterials can be assembled properly with active anticancer agents and targeting moieties. Usually, drug molecules are targeted to desired specific cells or organs via passive (size, property, etc.) or active (particular targeting ligands) targeting, in which passive targeting strategies are known as less efficient.^14,15^ The active targeting strategy involving carbohydrates is controllable and efficient and benefits from the carbohydrates’ well-defined chemical structure, biocompatibility, biodegradability, and water solubility for nanomedicine.^15−17^ Recently, graphene and its derivative graphene oxide (GO) have been utilized as theranostic nanocarriers due to their high specific surface area.^18,19^ Graphene and GO as two-dimensional single-layer sp^2^-bonded carbon atoms packed into a honeycomb lattice are indeed ideal platforms for highly efficient drug loading. This carbon allotrope has gained significant interest in life sciences due to its electronic, optical, and structural properties.^20−22^ In particular, GO with abundant functional groups of carboxylic acid, hydroxide, and epoxides can be loaded with various drugs by the non-covalent method via π–π stacking, hydrophobic/electrostatic interactions, and hydrogen bonding.^23^ It has been successfully employed as a novel vehicle for the delivery of biomolecules and drugs for cancer chemotherapy, ferro magnetics for hyperthermia, or PSs for photothermal therapy and PDT,^20,24−27^ which might increase PS load compared to free PS-mediated approaches. The key convertible element of PDT is the PS (i.e., the drug), which, upon irradiation, needs to be effectively excited to the triplet state for efficient generation of ^1^O_2_. The process of intersystem crossing (ISC) is electron spin forbidden; thus, a loophole is needed to enhance the upturn of the electron spin with a magnetic torque.^28,29^ To date, an extensive series of promising BODIPY-based triplet PSs have been developed via the general strategy of enhancing the triplet state formation by using the heavy-atom effect (mostly I or Br).^30−32^ Among the various fluorescent dyes, BODIPY derivatives are significantly stable in biological conditions. They bear unique photophysical properties, including narrow absorption/emission band, high absorption coefficients, and fluorescence quantum yields.^33−37^ As these heavy atom-functionalized BODIPY derivatives show tunable strong absorption of visible light and enhanced triplet state formation, it is very appealing to investigate the effects of GO introduction on their ISC efficiencies. 1,8-Naphthalimides (NI) are also conventional fluorescent dyes and are known as DNA-intercalating agents and anticancer compounds for applications as sensors and in medicine. The structural properties such as rigidity, planarity, and hydrophobicity of the skeleton makes NIs suitable for application in cancer treatment where the discovery of new antitumor agents is one of the most active research areas.^38−41^ Several groups have successfully employed GO as a nanotheranostic carrier of PSs for the destruction of tumor cells by light irradiation, but BODIPY-GO-based delivery systems are still not well established.^1,5,42−44^ Thus, it is of great interest to further develop tailor-made GO-based photoactive systems and investigate the effects of GO on the cytotoxic efficiencies of such systems against tumor cells.

Herein, we developed three novel heavy atom free (**10**), dibromo- (**11**), and diiodo- (**12**) distyryl-BODIPY derivatives ornamented with carbohydrates to endow them with active targeting ability toward tumor cells and NI moieties as an anticancer agent that can interact with GO via π–π stacking. The quantum yields of ^1^O_2_ of NI-BODIPY derivatives (**10**–**12**) were improved by the addition of GO compared with free PSs (**10**–**12**). Interestingly, even heavy atom free nanocomposite **GO**-**10** exhibited slightly higher ^1^O_2_ production than dyad **10**. Last, the *in vitro* studies revealed efficiency of anti-tumor features of the compounds.

## Results and Discussion

### Synthesis and Characterization

**Scheme 1:**
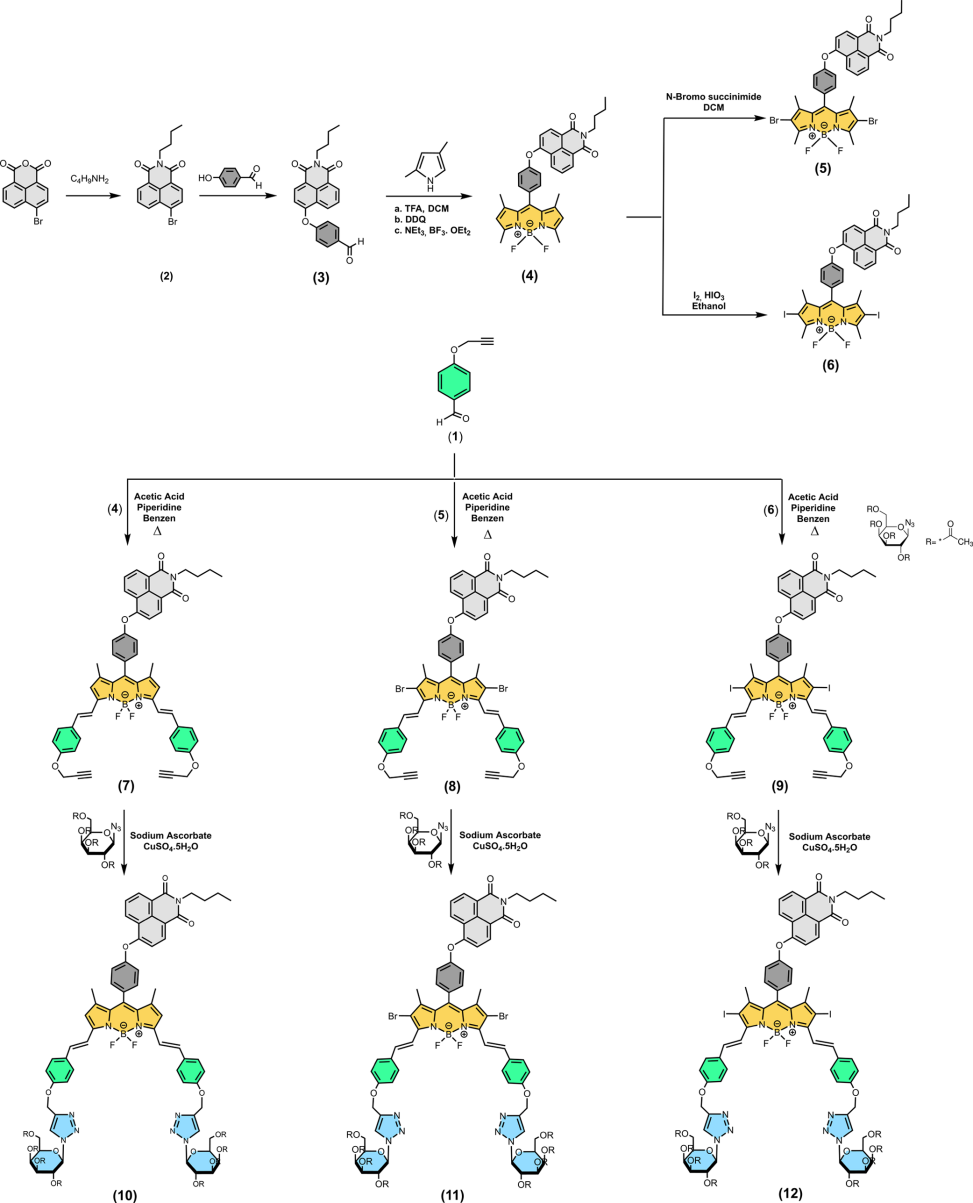
Synthesis Pathway of the NI-BODIPY-Glucose Dyads (**10**–**12**)

The synthesis of the three new glucose-substituted NI-BODIPY dyads (**10**–**12**) are presented in Scheme 1. First, we prepared a NI-BODIPY core according to our previous report.^45^ Dibromo- and diiodo-NI-BODIPY derivatives (**6** and **7**) were introduced by the reaction with *N*-bromosuccinimide (NBS) in DCM and I_2_/HIO_3_ in ethanol, respectively, to synthesize **11** and **12**. BODIPY cores **4**–**6** were functionalized with aldehyde groups via double Knoevenagel condensation for the alkyne introduction and to initiate light absorption in the photodynamic window. Two targeting units on the designed NI-BODIPYs (**10**–**12**) were provided by reacting distyryl BODIPYs **7**–**9** with a commercially available glucose derivative bearing azide under click reaction conditions in 25–29% yields. These yields are comparable to those observed in previous studies and can be attributed to the formation of mono-adducts in the reaction mixture, and the bis adducts were separated from the mono-adducts by column chromatography on silica gel.^46^ The structural properties of all new compounds were characterized by FT-IR, mass, ^1^H and ^13^C NMR spectroscopy techniques (Experimental Section, ESI Figures S1–S27). The NMR spectra of all targeted compounds have characteristic signals for protons and carbons located at the BODIPY fragment. The mass spectra of **10**–**12** were found to be in full agreement with their elemental composition, although in some cases both [M]^+^ and [M-F]^+^ ions were simultaneously observed. Well-resolved ^1^H NMR spectra of **10**–**12** showed sets of signals for meso-NI and aromatic protons in ∼6.9–9 ppm regions. The N=CH protons on triazole rings were observed at around 7.9–8.0 ppm, and the *trans* C=H protons were present at ∼7.6 and 7.2 ppm as doublets with ∼16.5 Hz coupling. The pyrrole ring −CH protons appeared as sharp singlets at ∼6.7 ppm for compound **10**, whereas the peak disappeared after the functionalization with Br or I. The −N–CH and −CH peaks belong to the glucose units were observed between 5.9 and 4.0 ppm while diastereotopic −CH_2_ protons differentiated and were detected at ∼4.3 and ∼4.2 ppm as quintets. The peak around 5.2 ppm is attributed to −OCH_2_ protons close to the triazole unit, and the triplet peak at 4.2 ppm was assigned to −NCH_2_ on imide moiety. The methyl protons on both glucose and the BODIPY core were placed between 2.0 and 1.0 ppm as singlets. The ^13^C NMR spectra of **10**–**12** showed peaks at ∼170 ppm, indicating that carbonyl carbons and between 150 and 115 ppm aromatic carbons were resonated. The aliphatic carbon atoms were observed between 85 and 14 ppm. The ^13^C NMR spectra of all dyads exhibited a similar NMR pattern.

**Figure 1:**
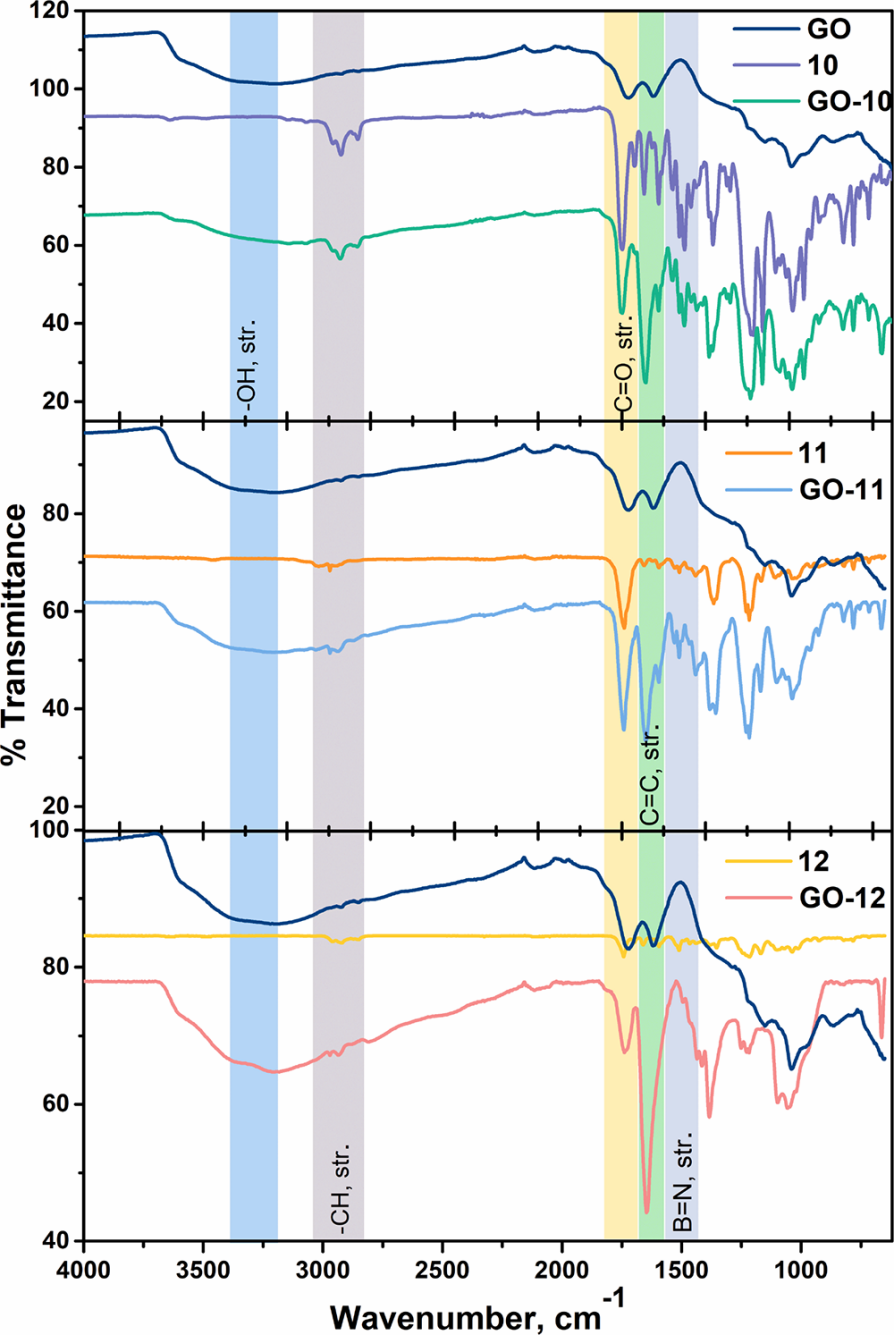
FT-IR spectra of GO, compounds **10–12**, and **GO-(10–12)**.

**Figure 2:**
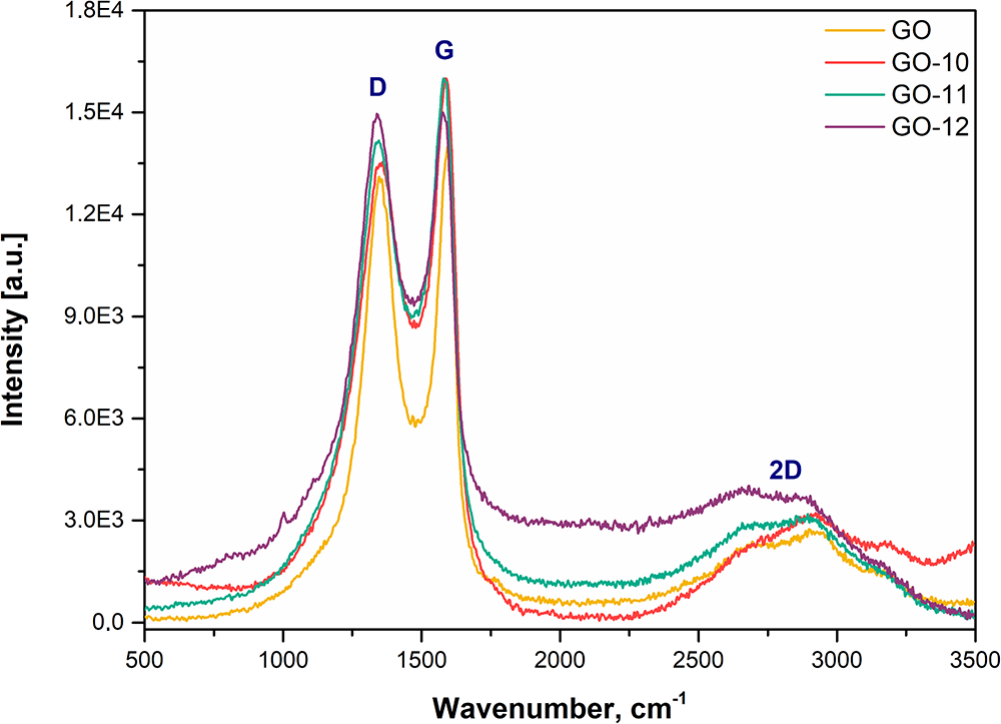
Raman spectra of GO and **GO-**(**10–12**) nanocarriers.

**Scheme 2:**
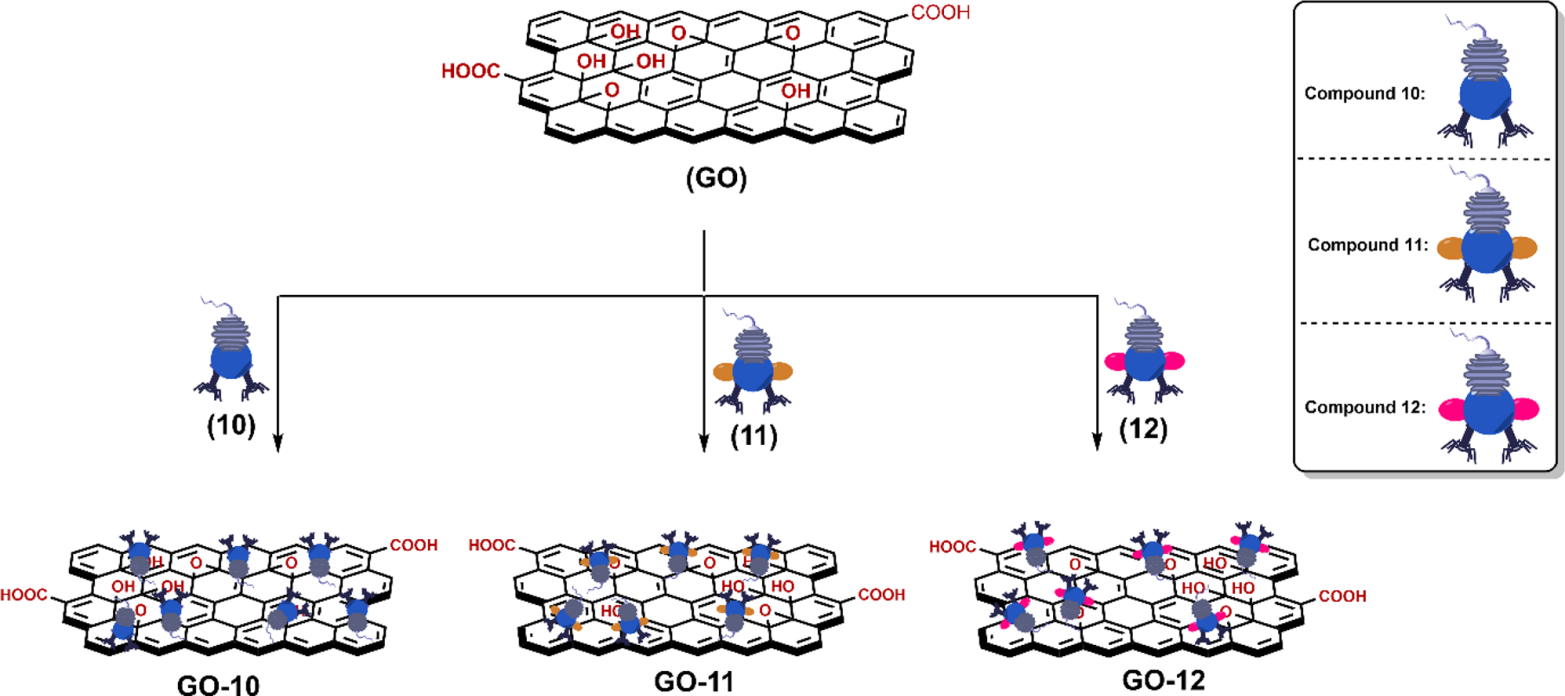
Schematic Representation of NI-BODIPY-GO Nanocomposites

The desired nanocarriers NI-BODIPY-GO **GO-**(**10–12**) were prepared successfully via the sonication-assisted exfoliation of GO then adsorption of drug by a non-covalent interaction (Scheme 2). The loading of novel PSs to the carrier was achieved by stirring the carrier and **10**–**12** for 40 h at room temperature to give **GO**-(**10**–**12**) nanocomposites. The formation of NI-BODIPY-GO conjugates was first confirmed via FT-IR spectroscopy. The FT-IR spectra of GO-based nanocomposites were displayed comparatively (Figure 1). In addition to the sp^2^-hybridized carbons on the network of GO’s present π–π interaction with NI moieties, carboxylic acid/alcohol functional groups can also form strong non-covalent interactions like hydrogen bonding, van der Waals interactions, and electrostatic attractions with NI-BODIPY dyads (**10**–**12**). For **GO-10**, the broad peak is located at 3271.99 cm^–1^ corresponding to −OH stretching on GO. The peaks located at 2965.53, 2925.39, and 2850.45 cm^–1^ were aromatic C–H stretching, and the peaks belonging to C=O groups on both GO and NI-BODIPY were observed at 1754.42 cm^–1^. The peaks between 1652.72 and 1593.83 cm^–1^ are attributed to C=C, and the peaks around 1590 and 1208.42 cm^–1^ are assigned to B–N and C–N moieties, respectively. Structural properties of the NI-BODIPY-GO composites were also revealed via Raman spectroscopy obtained at an excitation wavelength of 532 nm (Figure 2). In the spectrum of GO, three typical modes are observed. The D band at 1356 cm^–1^ is related to the presence of a certain amount of sp^3^ carbon atoms due to amorphization and functionalization of graphite during the oxidation process. The G mode at 1596 cm^–1^ originates from the in-plane vibration of sp^2^ carbon atoms.^1^ The broad band at 2685 cm^–1^ is assigned as a 2D mode corresponding to double resonance transitions resulting in the production of two phonons with opposite momentums, whereas the weak and broad 2D peaks are an indication of disorder.^47^ Since weak and broad 2D peaks are another indication of disorder and D peak, which is Raman active only in the presence of defects, the 2D peak is active even in the absence of any defects.

**Figure 3:**
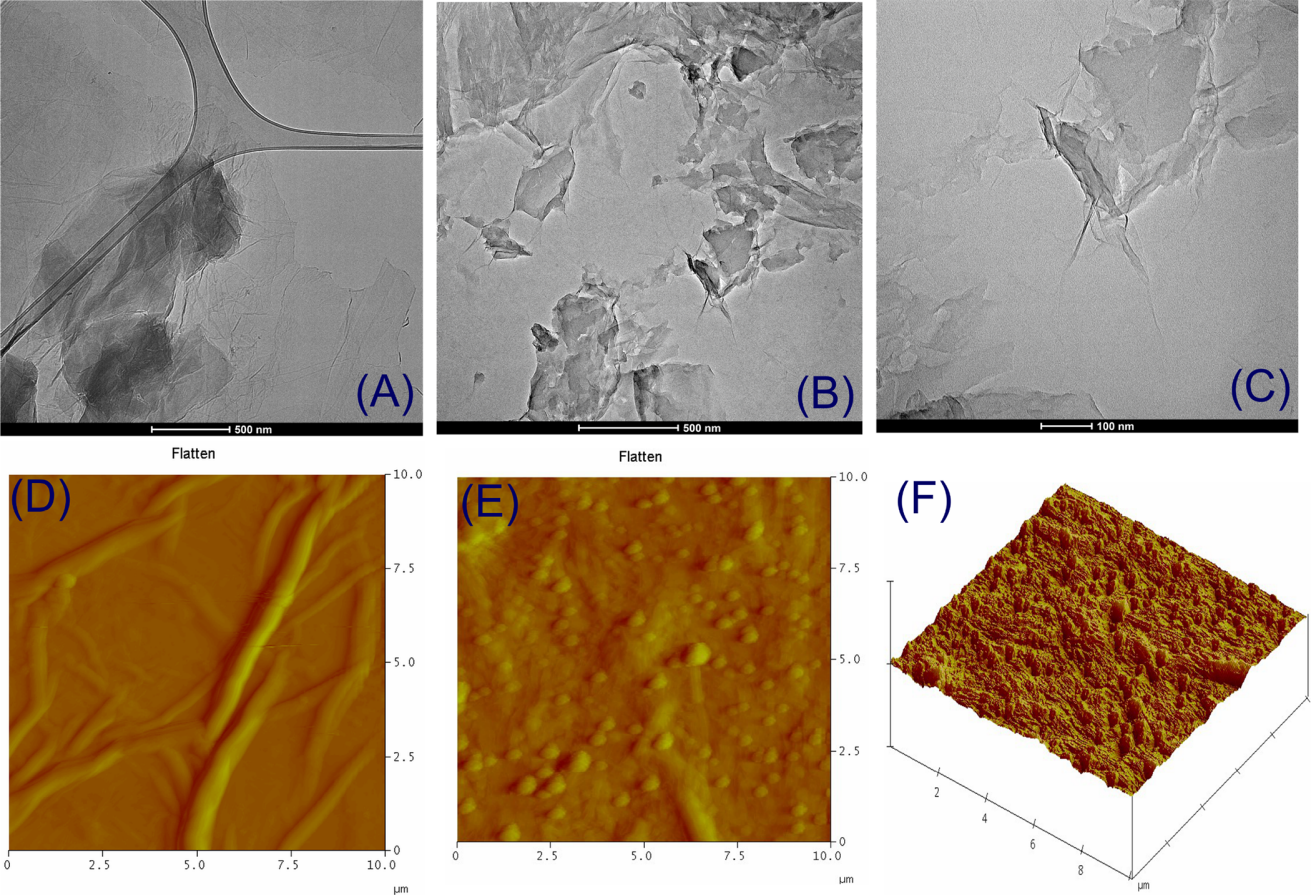
TEM images of (A) **GO** and (B, C) **GO**-**10** and AFM images of (D) GO, and (E, F) **GO-10** nanocomposite sheets.

The TEM micrographs of synthesized **GO**-(**10**–**12**) nanocarriers with different scale bars are given in Figure 3 and Figures S28–S30. From the figures, it is possible to distinguish the edges of sheets, including wrinkled areas. It is observed that GO has a layered ultrathin structure, and after the functionalization and sonication, the flakes were observed to be shapeshifted and ruptured. The EDX analysis also displayed the presence of anticipated elements such as N, C, and O (Figures S31–S33). The 3D structure of the nanocarriers along with AFM images is also shown in Figure 3 and Figures S34–S36. The samples for AFM explorations were prepared by drop-casting ultrasonicated solutions (0.1 mg/ mL in water) of **GO**-(**10**–**12**) on an unsoiled glass surface. The topography and average roughness of nanocomposite indicate that the morphology of material has been changed by coverage of the synthesized material, thereby confirming non-covalent attachment of BODIPY.

### Photophysical Properties

**Figure 4:**
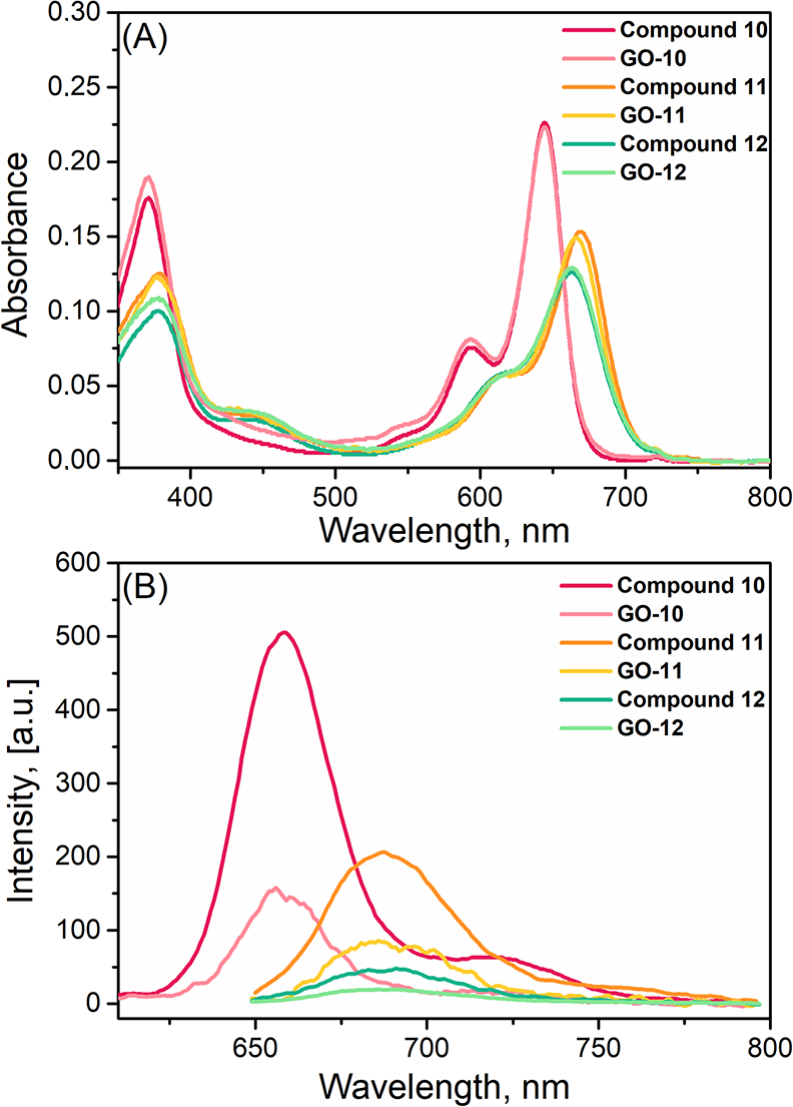
(A) Absorbance and (B) fluorescence emission spectra of compounds **10**–**12** and **GO-(10–12)** in dichloromethane (2 μM).

The UV–vis absorption profiles of compounds **10–12** and nanocarriers **GO-**(**10–12**) were investigated in different solvents such as acetone, chloroform, dichloromethane, dimethyl sulfoxide, water:dimethyl sulfoxide (99:1 and 95:5, v:v), acetonitrile, methanol, and tetrahydrofuran at 2 μM concentration (Figures S37–S42). The absorption spectra of heavy atom free NI-BODIPY **10** and related nanocomposite (**GO**-**10**) in organic solvents with different polarities except methanol remained nearly the same (λ_max_ ∼ 644 nm), whereas the absorbance intensities in water:dimethyl sulfoxide (99:1 and 95:5, v:v) systems were almost zero and the broadened and red-shifted peak formation was coherent with the aggregation.^48^ Compounds **11** and **12** and related nanocarriers (**GO**-**11** and **-12**) showed a maximum absorption peak between 654 and 669 nm with a vibrational peak around 600 nm. Distyryl BODIPY derivatives **10**–**12** in dichloromethane displayed a maximum absorption peak at ∼660 nm responsive to the lowest-energy spin-allowed S_0_–S_1_ transitions.^45^ Furthermore, there was a second characteristic peak around 370–380 nm due to the S_0_–S_2_ transition (Figure 4A). Molar absorption coefficients (ε) of the BODIPYs (**10**–**12**) were calculated by plotting maximum absorbance against concentration in dichloromethane (12.02, 9.03, and 7.66 × 10,^4^ respectively) (Table 1 and Figures S43–S45). Unsubstituted NI-BODIPY **10** showed an emission maximum in the studied solvent between 652 and 665 nm when excited at 590 nm with florescence quantum yield of 0.68. As expected, after the formation of the nanocomposite influenced the emission property, the fluorescence emission intensity of nanocomposite **GO**-**10** decreased explicitly (Figures S46 and S47, Table 1). The fluorescence profiles of dibromo- (**11**) and diiodo- (**12**) NI-BODIPYs were found to be much weaker (λ_em_ = 687 nm) (Φ_F_ = 0.31 and 0.15, respectively) and even quenched for the **GO**-**11** and **GO**-**12** nanocomposites when excited at 635 nm in organic solvents with florescence quantum yields of 0.28 and 0.10 (dichloromethane), respectively (Figure 4B, Figures S48–S51). Stokes shifts of the dyads were about 12 nm. The fluorescence lifetimes of the dyads and nanocomposites were also in the range of 1–3 ns (Figure S52). All photophysical parameters including fluorescence lifetime and fluorescence quantum yields are given in Table 1, and the related graphs are in the Supporting Information.

**Table 1:** Photophysical and Photochemical Properties of NI-BODIPYs (**10**–**12**) and NI-BODIPY-GO Nanocarriers

compound number	absorption λ_abs_ (nm)	emission λ_em_ (nm)	Stokes shift (nm)	ε^a^	Φ_F_^b^	τ_F_ (ns)^c^	Φ_Δ_^d^
**10**	371, 593, 644	659	15	12.02	0.68	3.08	−e
**11**	379, 616, 669	688	19	9.03	0.31	3.55	0.45
**12**	378, 614, 664	691	27	7.66	0.15	2.13	0.50
**GO-10**	371, 593, 644	657	13		0.51	3.89	−e
**GO-11**	377, 615, 666	687	21		0.28	3.88	0.48
**GO-12**	379, 614, 663	693	30		0.10	2.31	0.57

a Molar extinction coefficient, dichloromethane, 10^4^ (M^–1^ cm^–1^).

b Fluorescence quantum yield.

c Fluorescence lifetime.

d Singlet oxygen quantum yield.

e Below 1%.

### Detection of ^1^O_2_ Generation in Solution

**Figure 5:**
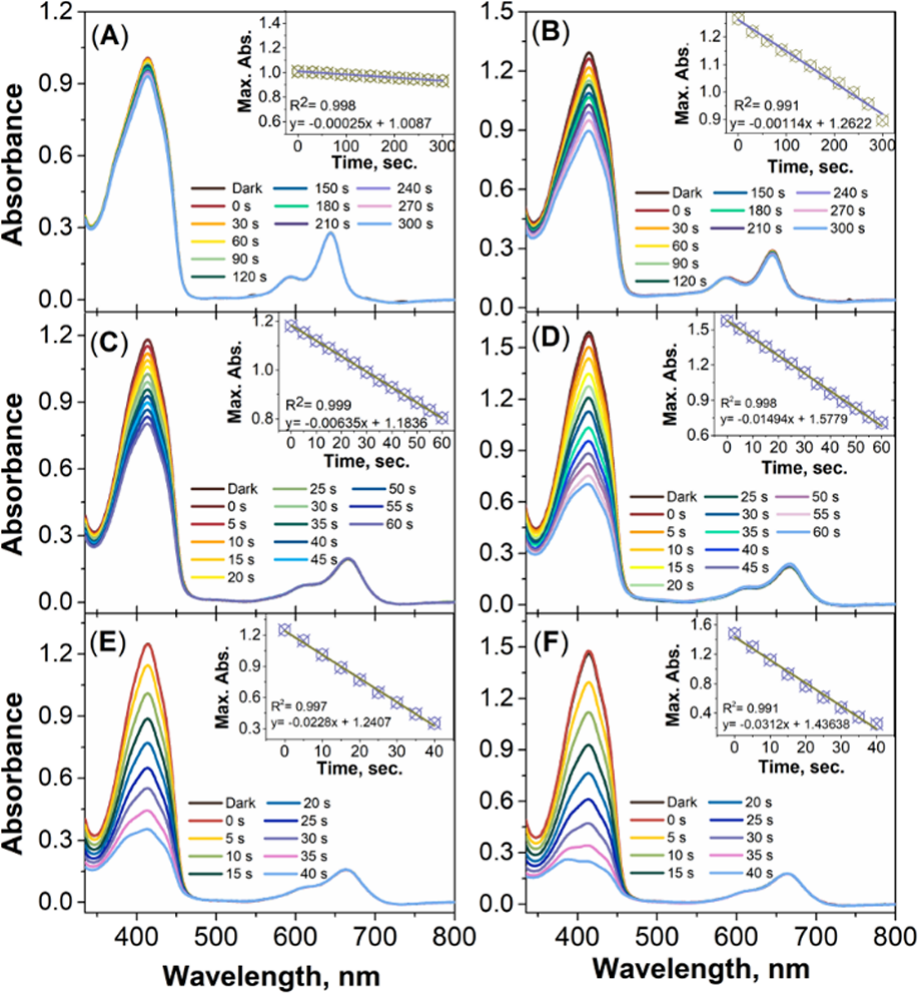
Decline in the absorbance of singlet oxygen trap molecule DPBF in the presence of (A) **10**, (B) **GO-10**, (C) **11**, (D) **GO-11**, (E) **12**, and (F) **GO-12** in dichloromethane (2 μM) upon irradiation.

In order to evaluate the photosensitizing properties of these NI-BODIPY derivatives and their GO-based nanocarriers, ^1^O_2_ quantum yields in organic solvent were first studied via the general procedure using 1,3-diphenylisobenzofuran (DPBF) as a ^1^O_2_ scavenger and methylene blue (MB) as the standard, which has a ^1^O_2_ yield of 0.57 in dichloromethane (Figure S53).^49^ The ROS generation abilities of targeted NI-BODIPY dyads and NI-BODIPY-GO nanocarriers (2 μM) with 630 nm LED irradiation were investigated. Absorption of DPBF degraded by ROS production from PSs did not change in the dark, which proves the lack of dark toxicity. In line with this, heavy atom free NI-BODIPY **10** and related nanocarrier **GO-10** were initially treated with light in DCM solution. Irradiation of the compound **10** solution at 630 nm did not cause a gradual decrease in the absorption signal of DBPF at 414 nm, whereas a minimal gradual decrease was observed for **GO-10**, which clearly suggests photosensitized ^1^O_2_ generation after the formation of the nanocomposite (Figure 5A,B). The ROS generation induced by **11** and **12** in dichloromethane upon light irradiation proved the efficient formation of ^1^O_2_ using DPBF, whose absorption significantly quenched. ^1^O_2_ quantum yields were calculated by using MB as a reference PS (Figure S53) and found to be 0.45 and 0.50 for **11** and **12** and 0.48 and 0.57 for **GO-11** and **GO-12**, respectively (Table 1). The experiment was also repeated in the presence of GO to control the possible interference of GO alone to ^1^O_2_ quantum yields. GO did not produce a considerable amount of ^1^O_2_ (Figure S54). Studies via theoretical calculations and experimental data showed that thanks to the electronic properties, graphene and its derivatives can act as an efficient fluorescence quencher for fluorophores.^13,50−52^ This quenching may contribute to the formation of ROS for the benefit of ^1^O_2_. In addition, the DPBF decrease rates of most dyads were larger than MB (Figure S55). As anticipated, the quantum yields of the dyads were increased with the addition of GO. Furthermore, as a water-soluble ^1^O_2_ selective trap molecule ^1^O_2_ sensor, 9,10-anthracenediylbis(methylene)dimalonic acid (ABDA) was employed to demonstrate ^1^O_2_ generation from developed PSs and nanocarriers in aqueous solutions (1% dimethyl sulfoxide in PBS, pH 7.4). ABDA produces corresponding endoperoxides, which cause changes in its absorption bands at 360, 380, and 400 nm when it reacts with ^1^O_2_. Compounds **10**–**12** and **GO-(10**–**12**) (10 μM) were placed in a cuvette for 30 min at 25 °C, and then the absorption of the PSs was first checked to investigate the dark side reactions and possible solubility problems. When the mixtures were irradiated with a 660 nm LED (irradiation intensity of 25 mW/ cm^2^) for 10 min, the characteristic absorption bands of ABDA were gradually decreased (Figure S56). Thus, these results confirmed the generation of ^1^O_2_ from NI-BODIPY dyads and GO-based nanocarriers in aqueous conditions.

### Effects of NI-BODIPY Derivatives and GO-Based Nanocarriers on Cancer Cells

**Figure 6:**
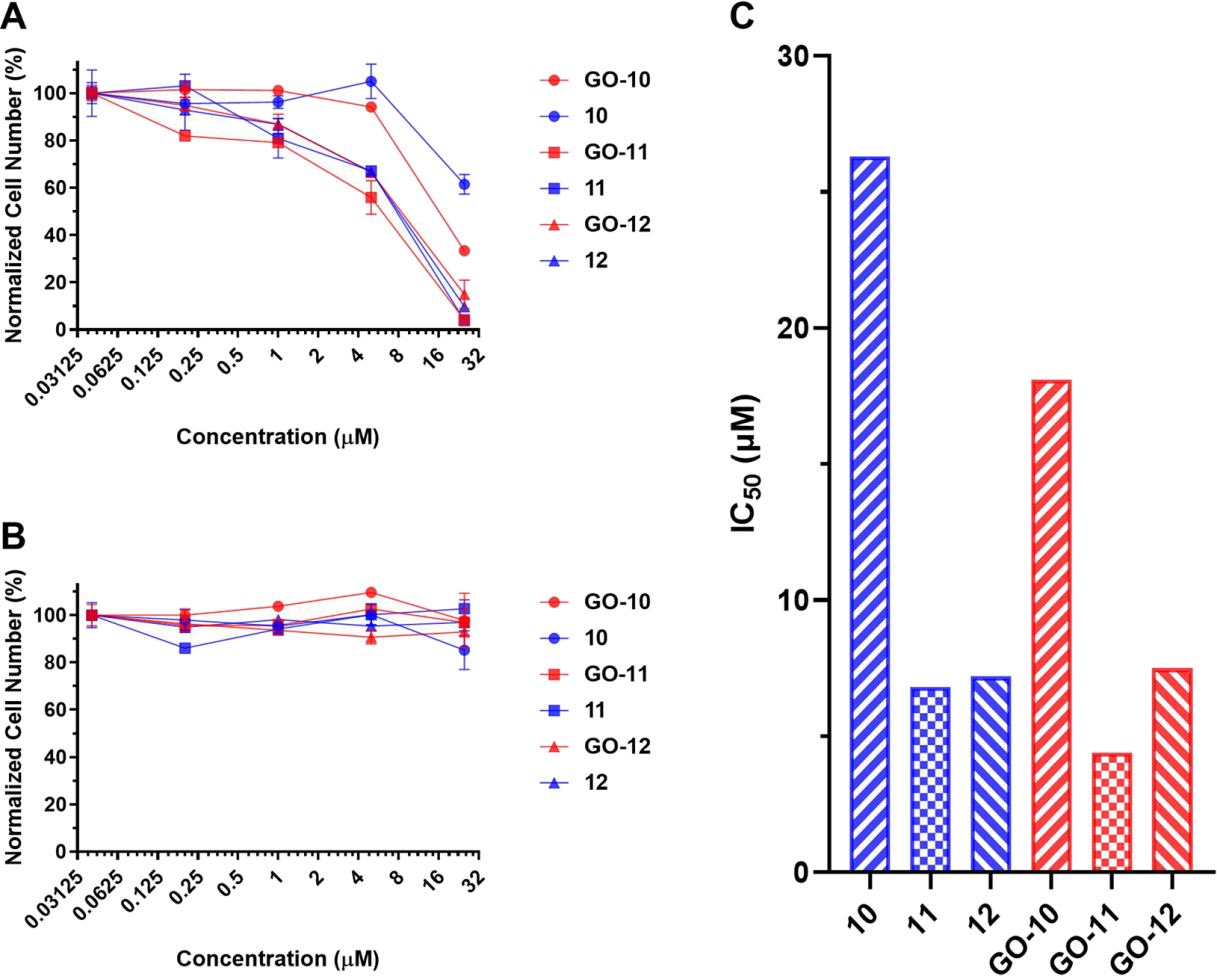
(A) The effects of compounds **10**–**12** and **GO-(10–12)** on K562 cancer cells when the cells were illuminated with a light source for 8 h and then kept in the dark for 40 h. (B) The effects of compounds **10**–**12** and **GO-(10–12)** on K562 cancer cells when the cells were kept in the dark for 48 h. (**C**) IC_50_ values of compounds **10**–**12** and **GO-(10–12)** when the cells were illuminated with a light source for 8 h and then kept in the dark for 40 h (estimated by fitting models with nonlinear regression).

PDT effects of NI-BODIPY derivatives as well as their GO-based nanocarriers were investigated in *in vitro* cell cultures. Varying concentrations of **10**–**12** and **GO-**(**10–12**) were incubated with K562 cells (human cancer suspension cell line-chronic myelogenous leukemia). Cells were illuminated with a light source for 8 h and then kept in the dark for 40 h to provide sufficient time for apoptosis. Cytotoxic effects of the chemicals were analyzed via MTT (3-(4,5-dimethylthiazol)-2,5-diphenyltetrazolium bromide) assay. Cell viabilities decrease gradually as the concentrations of the drugs increase (Figure 6A). The IC_50_ values of the compounds (**10**–**12**) were calculated to be 26.3, 6.8, and 7.2 μM (respectively), demonstrating the high efficacy of the NI-BODIPY derivatives on cancer cells (Figure 6C). Compounds **10**–**12** demonstrated negligible dark toxicity as seen by the high survival rate of the cells when the cells were kept in the dark (and treated with **10**–**12**) (Figure 6B). The IC_50_ values of GO-loaded NI-BODIPY derivatives [**GO-**(**10–12**)] were calculated as 18.1, 4.4, and 7.5 μM (respectively), demonstrating the higher efficacy of the NI-BODIPY-GO nanocarriers on cancer cells (Figure 6C). The K562 cells incubated with 25 μM compound **10** demonstrated about 61% cell viability, while K562 cells showed about 33% viability at the same concentration of compound **GO-10** (Figure 6A). Adding GO to compounds **10**–**12** seems to increase the potency of the drugs against cancer cells, since the IC_50_ values decreased for the compounds in comparison to their counterparts that were not loaded onto GO. Surprisingly, the positive effect of GO on the efficacy of **10** was observed to be higher than for **12**. Compound **12** already has a very potent IC_50_ in which the expected affirmative effect of GO on **12** may not be clearly seen under experimental conditions. On the other hand, among the synthesized compounds, **10** showed the highest IC_50_ value and the effect of GO is clearly seen by adding GO. Furthermore, **GO-**(**10–12**) demonstrated negligible dark toxicity as seen by the high survival rate of the cells when the cells were kept in the dark (and treated with **GO-**(**10–12**) (Figure 6B).

## Conclusions

In summary, we synthesized three new red absorbing NI-BODIPY derivatives (**10**–**12**), which were ornamented with carbohydrates to endow them with active targeting ability toward tumor cells and napthalimide moieties as the anticancer agent. Given the fact that GO has recently been utilized as an efficient theranostic nanocarrier, herein, we developed three novel NI-BODIPY-GO [**GO-**(**10**–**12**)] nanocarriers, since both napthalimide and carbohydrate moieties are capable of interacting with GO via π–π stacking, electrostatic interactions, and hydrogen bonding. NI-BODIPY-GO [**GO-**(**10**–**12**)] were prepared successfully via the sonication-assisted exfoliation of GO then adsorption of drug by a non-covalent interaction. Indeed, the quantum yields of ^1^O_2_ generation of NI-BODIPY derivatives (**10**–**12**) were significantly improved by the addition of GO. Even the ^1^O_2_ production capacity of heavy atom free nanocomposite **10** can be augmented by adding GO (**GO**-**10**). Our results concerning ^1^O_2_ generation are in strong agreement with the findings that GO might represent an ideal platform for highly efficient drug loading. Furthermore, the ^1^O_2_ production ability of **10**–**12** and **GO**-(**10**–**12**) was evaluated in cell culture studies against cancer cells. The results of both studies confirmed the generation of ^1^O_2_ from NI-BODIPY dyads and GO-based nanocarriers in aqueous conditions as well as demonstrating their anti-tumor efficiencies. According to the literature, numerous promising BODIPY-based PSs have been developed via the general strategy of enhancing the triplet state formation by using the heavy-atom effect. However, we propose to utilize GO in order to achieve highly efficient PDT by means of potentiating the PSs utilized in PDT. Our results clearly show that GO-based nanocarriers for BODIPY derivatives provide a propitious treatment approach for tumors. Given the biological effectiveness of our newly synthesized compounds against tumor cells, our approach may open up new avenues for future applications of graphene and GO as theranostic nanocarriers. We strongly think it is pivotal to further develop custom-made platforms that stem from GO nanocarriers in order to achieve highly effective PDT for cancer treatment.

## Experimental Section

### Materials

The deuterated solvent (CDCl_3_) used for NMR spectroscopy, silica gel 60 (230–400 mesh) for column chromatography, trifluoroacetic acid, *p*-chloranil, MB, DPBF, triethylamine, benzene, acetonitrile, sodium ascorbate, copper sulfate pentahydrate, cresyl violet, and boron trifluoride diethyl etherate were provided by Merck. The following chemicals were obtained from Sigma-Aldrich: ethanol, methanol tetrahydrofuran, sodium thiosulfate, dimethyl sulfoxide, 2,4-dimethylpyrrole, *N*,*N*-dimethylformamide, acetone, dichloromethane, iodic acid, iodine, hexane, sodium sulfate, *N*-bromosuccinimide, and glacial acetic acid. *n*-Butylamine was purchased from Alfa Aesar. The following chemicals were obtained from Acros Organics: piperidine and 4-hydroxybenzaldehyde. Bromo-1,8-naphthalic anhydride was purchased from TCI. Zinc phthalocyanine and 1-azido-1-deoxy-β-d-glucopyranoside tetraacetate were purchased from ABCR. The rest of the chemicals used in the synthesis were of reagent grade unless otherwise specified.

### Equipment

Mass spectra were acquired in linear modes with an average of 50 shots on a Bruker Daltonics microflex mass spectrometer (Bremen, Germany) equipped with a nitrogen UV laser operating at 337 nm. ^1^H and ^13^C NMR spectra were recorded in CDCl_3_ solutions on a Varian 500 MHz spectrometer. Analytical thin-layer chromatography (TLC) was performed on silica gel plates (Merck, Kieselgel 60 Å, 0.25 mm thickness) with an F_254_ indicator. Column chromatography was performed on silica gel (Merck, Kieselgel 60 Å, 230–400 mesh). Electronic absorption spectra were recorded with a Shimadzu 2101 UV spectrophotometer in the UV–visible region. Fluorescence excitation and emission spectra were recorded on a Varian Eclipse spectrofluorometer using 1 cm pathlength cuvettes at room temperature. The fluorescence lifetimes were obtained using Horiba-Jobin-Yvon-SPEX Fluorolog 3-2iHR instrument with a FluoroHub-B single-photon counting controller. Signal acquisition was performed using a time-correlated single photon counting (TCSPC) module. AFM images were recorded by Digital Instruments NanoScope IV AFM device. Raman spectra were obtained via Bruker FRA 106/S. Talos F200S TEM 200 kV was used for TEM and EDX analysis.

### Synthesis

Synthesis compounds **1**–**4** were carried out according to the literature.^45,53^

### Synthesis of Compound **5**

In a 50 mL round-bottom flask, compound **4** (0.15 g; 0.25 mmol) was dissolved in 30 mL of dichloromethane. *N*-Bromosuccinimide (0.2 g; 0.64 mmol) was dissolved in 10 mL of DCM and added to the reaction mixture dropwise. After the addition, the reaction mixture was stirred at room temperature for 3 h. The reaction mixture was extracted with water (200 mL, three times) and the organic layer was dried over anhydrous Na_2_SO_4_ and concentrated on a rotary evaporator until the solvent was removed. Compound **5** was isolated from column chromatography on silica gel (230–400 mesh) with dichloromethane as the eluent (yield: 64%).

#### Spectral Data of Compound **5** (Figures S1–S3)

^1^H NMR (500 MHz, CDCl_3_, 298 K, δ ppm): 8.69 (d, *J* = 7.67 Hz, 1H, Ar–CH), 8.67 (d, *J* = 7.97 Hz, 1H, Ar–CH), 8.52 (d, *J* = 8.31 Hz, 1H, Ar–CH), 7.81 (t, *J* = 7.74 Hz, 1H, Ar–CH), 7.39 (d, *J =* 8.49 Hz, 2H, Ar–CH), 7.35 (d, *J* = 8.26 Hz, 2H, Ar–CH), 6.99 (d, *J* = 8.20 Hz, 1H, Ar–CH), 4.19 (t, *J* = 7.51 Hz, 2H, N–CH_2_), 2.63 (s, 6H, −CH_3_), 1.73 (q, *J* = 7.51 Hz, 2H, −CH_2_−), 1.54 (s, 6H, −CH_3_), 1.48–1.44 (m, 2H, −CH_2_−), 0.99 (t, *J* = 7.33 Hz, 3H, −CH_3_).^13^C NMR (126 MHz, CDCl_3_, 298 K, δ ppm): 164.19, 163.54, 158.60, 156.34, 154.44, 140.22, 132.44, 132.04, 131.36, 130.47, 130.44, 130.23, 129.80, 128.24, 126.82, 124.14, 122.86, 121.39, 111.34, 40.26, 30.25, 20.41, 14.00, 13.87, 13.74. MS (MALDI-TOF) (DIT) *m*/*z* (%). Calc.: 749.26, found: 749.59 [M]^+^, 728.154 [M-F]^+^.

### Synthesis of Compound **6**

In a 250 mL round-bottom flask, compound **4** (0.15 g, 0.25 mmol) and I_2_ (0.19 g, 0.76 mmol) were dissolved in 70 mL of ethanol. Iodic acid HIO_3_ (0.13 g, 0.76 mmol) was dissolved in 1 mL of water and added into the reaction mixture. The reaction mixture was stirred at 50 °C for a few hours until the reactant was consumed. Then, saturated sodium thiosulfate solution was added (50 mL) and it was stirred at room temperature for an additional 30 min. Then, it was extracted with water (200 mL, three times) and the organic layer was dried over anhydrous Na_2_SO_4_ and concentrated on a rotary evaporator until the solvent was removed. Compound **6** was isolated from column chromatography on silica gel (230–400 mesh) with dichloromethane as the eluent (yield: 40%).

#### Spectral Data of Compound **6** (Figures S4–S6)

^1^H NMR (500 MHz, CDCl_3_, 298 K, δ ppm): 8.69 (d, *J* = 7.40 Hz, 1H, Ar–CH), 8.67 (d, *J* = 7.39 Hz, 1H, Ar–CH), 8.53 (d, *J* = 8.17 Hz, 1H, Ar–CH), 7.81 (t, *J =* 7.84 Hz, 1H, Ar–CH), 7.38 (d, *J =* 8.46 Hz, 2H, Ar–CH), 7.34 (d, *J =* 8.65 Hz, 2H, Ar–CH), 6.99 (d, *J =* 8.14 Hz, 1H, Ar–CH), 4.19 (t, *J =* 7.53 Hz, 2H, N–CH_2_), 2.66 (s, 6H, −CH_3_), 1.73 (q, *J =* 7.52 Hz, 2H, −CH_2_−), 1.55 (s, 6H, −CH_3_), 1.49–1.44 (m, 2H, −CH_2_−), 0.99 (t, *J =* 7.34 Hz, 3H, −CH_3_). ^13^C NMR (126 MHz, CDCl_3_, 298 K, δ ppm): 157.25, 156.32, 144.95, 132.47, 132.05, 131.35, 130.24, 128.25, 126.83, 121.43, 111.32, 40.26, 30.26, 20.41, 17.29, 16.08, 13.86157.25, 156.32, 144.95, 132.47, 132.05, 131.35, 130.24, 128.25, 126.83, 121.43, 111.32, 40.26, 30.26, 20.41, 17.29, 16.08, 13.86. MS (MALDI-TOF) (NOM) *m*/*z* (%). Calc.: 843.25, found: 843.030 [M]^+^, 824.014 [M-F]^+^.

### Synthesis of Compounds **7**–**9**

In a three-necked 100 mL round-bottom flask, BODIPY derivatives (**4**–**6**) (1 eqv.) and compound **1** (2.4 eqv.) were dissolved in 40 mL of benzene. Piperidine (0.3 mL) and glacial acetic acid (0.3 mL) were added. The solution was refluxed using a Dean–Stark apparatus. When the solution was concentrated, the reaction was followed by TLC until the starting compound (**4**–**6**) was consumed. The reaction mixture was extracted with dichloromethane/water (200 mL, three times), and the organic layer was dried over anhydrous Na_2_SO_4_ and concentrated on a rotary evaporator until the solvent was removed. Compounds **7**–**9** were isolated by column chromatography on silica gel (230–400 mesh).

#### Spectral Data of Compound **7** (Figures S7–S9)

^1^H NMR (500 MHz, CDCl_3_, 298 K, δ ppm): 8.71 (d, *J =* 8.65 Hz, 1H, Ar–CH), 8.69 (d, *J =* 8.14 Hz, 1H, Ar–CH), 8.52 (d, *J =* 8.19 Hz, 1H, Ar–CH), 7.82 (t, *J =* 7.81 Hz, 1H, Ar–CH), 7.65–7.60 (m, 4H + 2H, Ar–CH + *trans* C=H), 7.46 (d, *J =* 8.18 Hz, 2H, Ar–CH), 7.34 (d, *J =* 8.18 Hz, 2H, Ar–CH), 7.24 (d, *J =* 16.31 Hz, 2H, *trans* C=H), 7.03 (d, *J =* 8.40 Hz, 4H, Ar–CH), 6.96 (d, *J =* 8.19 Hz, 1H, Ar–CH), 6.67 (s, 2H, pyrrole −CH), 4.75 (d, *J =* 2.41 Hz, 4H, O–CH_2_), 4.20 (t, *J =* 7.46 Hz, 2H, −NCH_2_), 2.56 (t, *J =* 2.30 Hz, 2H, C ≡ CH), 1.73 (q, *J =* 7.57 Hz, 2H, −CH_2_−), 1.60 (s, 6H, −CH_3_), 1.49–1.44 (m, 2H, −CH_2_−), 0.99 (t, *J =* 7.47 Hz, 3H, −CH_3_). ^13^C NMR (126 MHz, CDCl_3_, 298 K, δ ppm): 164.27, 163.62, 159.10, 158.33, 152.95, 141.39, 136.74, 135.89, 133.23, 132.57, 132.50, 132.00, 130.92, 130.31, 129.78, 129.01, 128.34, 126.71, 122.85, 121.25, 117.84, 117.58, 117.37, 115.25, 55.88, 30.26, 29.70, 22.34, 20.41, 14.92, 14.06, 13.86.MS (MALDI-TOF) (DHB) *m*/*z* (%). Calc.: 875.76, found: 875.389 [M]^+^, 855.953 [M-F]^+^.

#### Spectral Data of Compound **8** (Figures S10–S12)

^1^H NMR (500 MHz, CDCl_3_, 298 K, δ ppm): 8.69 (d, *J =* 7.47 Hz, 1H + 1H, Ar–CH), 8.54 (d, *J =* 8.17 Hz, 1H, Ar–CH), 8.13 (d, *J =* 16.78 Hz, 2H, *trans* C=H), 7.82 (t, *J =* 7.78 Hz, 1H, Ar–CH), 7.66–7.62 (m, 4H + 2H, Ar–CH + *trans* C=H), 7.42 (d, *J =* 8.41 Hz, 2H, Ar–CH), 7.36 (d, *J =* 8.51 Hz, 2H, Ar–CH), 7.05 (d, *J =* 8.58 Hz, 4H, Ar–CH), 7.01 (d, *J =* 8.16 Hz, 1H, Ar–CH), 4.76 (d, *J =* 2.14 Hz, 4H, O–CH_2_), 4.20 (t, *J =* 7.50 Hz, 2H, −NCH_2_), 2.56 (t, *J =* 2.13 Hz, 2H, C ≡ CH), 1.74 (q, *J =* 7.52 Hz, 2H, −CH_2_−), 1.55 (s, 6H, −CH_3_), 1.49–1.44 (m, 2H, −CH_2_−), 0.99 (t, *J =* 7.28 Hz, 3H, −CH_3_). ^13^C NMR (126 MHz, CDCl_3_, 298 K, δ ppm): 167.78, 132.47, 130.89, 129.32, 128.81, 115.32, 68.17, 67.99, 55.89, 53.43, 38.74, 34.13, 30.37, 29.72, 28.93, 25.62, 23.75, 22.99, 22.35, 14.06, 10.97. MS (MALDI-TOF) (DHB) *m*/*z* (%). Calc.: 1033.55, found: 1033.150 [M]^+^, 1014.258 [M-F]^+^.

#### Spectral Data of Compound **9** (Figures S13–S15)

^1^H NMR (500 MHz, CDCl_3_, 298 K, δ ppm): 8.70 (d, *J =* 7.73 Hz, 1H + 1H, Ar–CH), 8.54 (d, *J =* 7.93 Hz, 1H, Ar–CH), 8.16 (d, *J =* 16,65 Hz, 2H, *trans* C=H), 7.83 (t, *J =* 7.73 Hz, 1H, Ar–CH), 7.65–7.59 (m, 4H + 2H, Ar–CH + *trans* C=H), 7.42 (d, *J =* 7.93 Hz, 2H, Ar–CH), 7.36 (d, *J =* 7.73 Hz, 2H, Ar–CH), 7.04 (d, *J =* 8.22 Hz, 4H, Ar–CH), 7.01 (d, *J =* 8.07 Hz, 1H, Ar–CH), 4.76 (d, *J =* 2.02 Hz, 4H, O–CH_2_), 4.20 (t, *J =* 7.22 Hz, 2H, −NCH_2_), 2.56 (t, *J =* 2.09 Hz, 2H, C ≡ CH), 1.74 (q, *J =* 7.64 Hz, 2H, −CH_2_−), 1.62 (s, 6H, −CH_3_), 1.49–1.45 (m, 2H, −CH_2_−), 0.99 (t, *J =* 7.26 Hz, 3H, −CH_3_). ^13^C NMR (126 MHz, CDCl_3_, 298 K, δ ppm): 163.57, 158.72, 150.88, 145.31, 139.31, 130.78, 129.26, 123.95, 123.38, 121.41, 115.29, 53.41, 30.92, 30.27, 29.70, 29.28, 25.61, 20.41, 17.83, 14.24, 13.88, 9.70. MS (MALDI-TOF) (DIT) *m*/*z* (%). Calc.: 1127.56, found: 1127.081 [M]^+^, 1108.302 [M-F]^+^.

### Synthesis of Compounds **10**–**12**

BODIPY derivatives (**7**–**9**) (1 eqv.) and 1-azido-1-deoxy-β-d-glucopyranoside tetraacetate (3 eqv.) were dissolved in 20 mL tetrahydrofuran/water (3:1; v:v) in a two-necked round-bottom flask. CuSO_4_·5H_2_O (0.2 eqv.) and sodium ascorbate (0.5 eqv.) were added to this solution. The reaction mixture was refluxed at 60 °C for 72 h. The reaction mixture was extracted with dichloromethane/water, and the organic layer was dried over anhydrous Na_2_SO_4_ and concentrated on a rotary evaporator until the solvent was removed. Compounds **10**–**12** were isolated by column chromatography on silica gel (230–400 mesh).

#### Spectral Data of Compound **10** (Figures S16–S19)

FT-IR (ATR, cm^–1^): 2965.53 (C–H, str), 2925.39 (C–H, str), 2850.45 (C–H, str), 1754.42 (C=O, str), 1652.72 (C=C, str), 1593.83 (C=C, str), 1490.79 (B–N, str), 1383.73 (C–H, bending), 1208.42 (C–N, str), 1037.12 (C–O, str). ^1^H NMR (500 MHz, CDCl_3_, 298 K, δ ppm): 8.70 (d, *J* = 8.78 Hz, 1H, Ar–CH), 8.68 (d, *J* = 7.24 Hz, 1H, Ar–CH), 8.51 (d, *J* = 8.11 Hz, 1H, Ar–CH), 7.89 (s, 2H, N=CH), 7.81 (t, *J* = 7.73 Hz, 1H, Ar–CH), 7.62 (d, *J* = 16,61 Hz, 2H, *trans* C=H), 7.59 (d, *J =* 8.43 Hz, 4H, Ar–CH), 7.45 (d, *J* = 8.13 Hz, 2H, Ar–CH), 7.33 (d, *J* = 8.02 Hz, 2H, Ar–CH), 7.23 (d, *J* = 16.40 Hz, 2H, *trans* C=H), 7.02 (d, *J* = 8.38 Hz, 4H, Ar–CH), 6.96 (d, *J* = 8.21 Hz, 1H, Ar–CH), 6.66 (s, 2H, pyrrole–CH), 5.90 (d, *J* = 8.89 Hz, 2H, Gly N–CH),5.47 (t, *J* = 9.10 Hz, 2H, Gly–CH), 5.42 (t, *J* = 9.20 Hz, 2H, Gly–CH), 5.27 (s, 4H, O–CH_2_), 5.25 (t, *J* = 9.23 Hz, 2H, Gly–CH), 4.31 (dd, *J*_1_ = 12.66 Hz, *J*_2_ = 5.05 Hz, 2H, diastereotopic Gly–CH), 4.19 (t, *J* = 7.69 Hz, 2H, −NCH_2_), 4.16 (dd, *J*_1_ = 12.94 Hz, *J*_2_ = 2.33 Hz, 2H, diastereotopic Gly–CH), 4.03–4.00 (m, 2H, Gly–CH), 2.09 (s, 6H, Gly–CH_3_), 2.07 (s, 6H, Gly–CH_3_), 2.02 (s, 6H, Gly–CH_3_), 1.86 (s, 6H, Gly–CH_3_), 1.73 (q, *J* = 7.52 Hz, 2H, −CH_2_−), 1.59 (s, 6H, −CH_3_), 1.48–1.44 (m, 2H, −CH_2_−), 0.99 (t, *J* = 7.41 Hz, 3H, −CH_3_). ^13^C NMR (126 MHz, CDCl_3_, 298 K, δ ppm): 170.53, 169.94, 169.35, 168.94, 158.92, 155.69, 144.75, 135.96, 132.04, 130.95, 130.11, 129.15, 124.09, 121.25, 115.22, 85.82, 72.65, 70.25, 67.70, 61.90, 61.53, 53.43, 30.27, 29.71, 20.72, 20.55, 20.53, 20.42, 20.14, 14.94, 13.88. MS (MALDI-TOF) (CHCA) *m*/*z* (%). Calc.: 1622.39, found: 1622.267 [M]^+^, 1645.309 [M + Na]^+^.

#### Spectral Data of Compound **11** (Figures S20–S23)

FT-IR (ATR, cm^–1^): 3014.04 (C–H, str), 2971.69 (C–H, str), 2937.55 (C–H, str), 1743.51 (C=O, str), 1646.20 (C=C, str), 1593.60 (C=C, str), 1509.45 (B–N, str), 1359.53 (C–H, bending), 1217.52 (C–N, str), 1033.42 (C–O, str). ^1^H NMR (500 MHz, CDCl_3_, 298 K, δ ppm): 8.69 (d, *J* = 7.84 Hz, 1H + 1H, Ar–CH), 8.53 (d, *J* = 8.17 Hz, 1H, Ar–CH), 8.12 (d, *J* = 16,60 Hz, 2H, *trans* C=H), 7.91 (s, 2H, N=CH), 7.82 (t, *J* = 7.83 Hz, 1H, Ar–CH), 7.64–7.61 (m, 4H + 2H, Ar–CH+ *trans* C=H), 7.41 (d, *J* = 8.42 Hz, 2H, Ar–CH), 7.35 (d, *J* = 8.37 Hz, 2H, Ar–CH), 7.05 (d, *J* = 8.60 Hz, 4H, Ar–CH), 7.00 (d, *J* = 8.17 Hz, 1H, Ar–CH), 5.91 (d, *J* = 9.09 Hz, 2H, Gly N–CH), 5.48 (t, *J* = 9.33 Hz, 2H, Gly–CH), 5.43 (t, *J* = 9.30 Hz, 2H, Gly–CH), 5.29 (s, 4H, O–CH_2_), 5.25 (t, *J* = 9.62 Hz, 2H, Gly–CH), 4.31 (dd, *J*_1_ = 12.59 Hz, *J*_2_ = 4.97 Hz, 2H, diastereotopic Gly–CH), 4.20 (t, *J* = 7.69 Hz, 2H, −NCH_2_), 4.16 (dd, *J*_1_ = 12.70 Hz, *J*_2_ = 1.75 Hz, 2H, diastereotopic Gly–CH), 4.04–4.00 (m, 2H, Gly–CH), 2.08 (s, 6H, Gly–CH_3_), 2.06 (s, 6H, Gly–CH_3_), 2.01 (s, 6H, Gly–CH_3_), 1.85 (s, 6H, Gly–CH_3_), 1.74 (q, *J* = 7.54 Hz, 2H, −CH_2_−), 1.58 (s, 6H, −CH_3_), 1.49–1.44 (m, 2H, −CH_2_−), 0.99 (t, *J* = 7.34 Hz, 3H, −CH_3_)^13^C NMR (126 MHz, CDCl_3_, 298 K, δ ppm): 170.54, 169.94, 169.36, 168.95, 164.27, 163.62, 161.12, 159.30, 145.37, 144.71, 139.06, 132.09, 130.84, 130.29, 129.40, 124.17, 122.92, 121.31, 117.69, 115.29, 85.81, 75.23, 72.66, 70.24, 67.72, 61.91, 61.55, 20.72, 20.55, 20.51, 20.42, 20.14, 14.06, 13.88. MS (MALDI-TOF) (DIT) *m*/*z* (%). Calc.: 1780.19, found: 1780.267 [M]^+^.

#### Spectral Data of Compound **12** (Figures S24–S27)

FT-IR (ATR, cm^–1^): 2960.05 (C–H, str), 2923.82 (C–H, str), 2849.81 (C–H, str), 1744.76 (C=O, str), 1644.53 (C=C, str), 1594.41 (C=C, str), 1506.67 (B–N, str), 1383.40 (C–H, bending), 1214.58 (C–N, str), 1040.50 (C–O, str). ^1^H NMR (500 MHz, CDCl_3_, 298 K, δ ppm): 8.76 (d, *J* = 7.25 Hz, 1H + 1H, Ar–CH), 8.54 (d, *J* = 7.88 Hz, 1H, Ar–CH), 8.16 (d, *J* = 16.36 Hz, 2H, *trans* C=H), 7.90 (s, 2H, N=CH), 7.82 (t, *J* = 7.89 Hz, 1H, Ar–CH), 7.62 (d, *J =* 7.62 Hz, 4H, Ar–CH), 7.60 (d, *J* = 16.82 Hz, 2H, *trans* C=H), 7.41 (d, *J* = 7.19 Hz, 2H, Ar–CH), 7.35 (d, *J* = 6.95 Hz, 2H, Ar–CH), 7.05 (d, *J* = 7.47 Hz, 4H, Ar–CH), 7.00 (d, *J* = 7.88 Hz, 1H, Ar–CH), 5.90 (d, *J* = 8.21 Hz, 2H, Gly N–CH), 5.48 (t, *J* = 9.50 Hz, 2H, Gly–CH), 5.42 (t, *J* = 8.41 Hz, 2H, Gly–CH), 5.29 (s, 4H, O–CH_2_), 5.25 (t, *J* = 9.17 Hz, 2H, Gly–CH), 4.31 (dd, *J*_1_ = 12.83 Hz, *J*_2_ = 3.66 Hz, 2H, diastereotopic Gly–CH), 4.20 (t, *J* = 7.65 Hz, 2H, −NCH_2_), 4.15 (dd, *J*_1_ = 12.81 Hz, *J*_2_ = 4.08 Hz, 2H, diastereotopic Gly–CH), 4.04–4.01 (m, 2H, Gly–CH), 2.08 (s, 6H, Gly–CH_3_), 2.07 (s, 6H, Gly–CH_3_), 2.01 (s, 6H, Gly–CH_3_), 1.85 (s, 6H, Gly–CH_3_), 1.74 (q, *J* = 6.80 Hz, 2H, −CH_2_−), 1.62 (s, 6H, −CH_3_), 1.49–1.44 (m, 2H, −CH_2_−), 0.99 (t, *J* = 6.62 Hz, 3H, −CH_3_)^13^C NMR (126 MHz, CDCl_3_, 298 K, δ ppm): 170.54, 169.94, 169.36, 168.95, 164.27, 159.25, 158.79, 150.82, 145.34, 144.72, 139.33, 132.96, 132.55, 132.38, 132.09, 130.82, 130.11, 129.83, 129.37, 128.32, 126.86, 124.16, 122.92, 121.46, 121.31, 117.68, 116.95, 115.28, 85.81, 75.22, 72.66, 70.24, 67.72, 61.91, 55, 53.44, 40.29, 30.27, 29.71, 20.72, 20.56, 20.52, 20.42, 20.14, 17.84, 13.88. MS (MALDI-TOF) (DIT) m/z (%). Calc.: 1874.19, found: 1897.425 [M + Na]^+^, 1874.305 [M]^+^, 1855.630 [M-F]^+^.

### Preparation of **GO-(10–12)** Nanocomposites

Commercial GO was used for the preparation of NI-BODIPY-GO nanocarriers according to the modified method previously reported elsewhere.^1^ Briefly, 20 mg of GO was dispersed in 60 mL of distilled water by ultrasonication (1.5 h) to obtain a homogeneous suspension of GO. Afterward, 20 mg of NI-BODIPY derivative (**10**–**12**) was added and magnetically stirred at room temperature for several minutes. Then, the mixture was stirred for 40 h for dye adsorption on GO at room temperature. The resulting mixture was filtered through a polycarbonate membrane with 0.2 mm pores, and the obtained solid material was washed with water several times to remove the excess of compounds **10**–**12** then dried in a vacuum oven for 48 h at 45 °C.

### Parameters for Fluorescence Quantum Yields

The fluorescence quantum yields (Φ_F_) of compounds **10–12** and nanocarriers **GO-(10–12)** were determined by the comparative method (eq 1).^54^
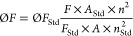
1where *F* and *F*_Std_ are the areas under the fluorescence emission curves of (**10**–**12**) and **GO-(10–12)** and the standard, respectively. *A* and *A*_Std_ are the respective absorbances at the excitation wavelengths. η is the refractive index of the solvents that were employed for calculating the fluorescence quantum yields. Two different standards were used to determine the fluorescence quantum yields: (i) cresyl violet (Φ_F_ = 0.53 in methanol)^54^ and (ii) zinc phthalocyanine (Φ_F_ = 0.20 in dimethyl sulfoxide).^55^

### The Parameters for ^1^O_2_ Quantum Yields

The ^1^O_2_ generating ability of compounds **10–12** and nanocarriers [**(GO-10-12)**] was employed in both dichloromethane and PBS. ^1^O_2_ trap molecules DPBF and ABDA were used. MB was studied as a reference triplet PS. ^1^O_2_ formation can be traced using photobleaching and subsequent decrease in absorbance of DPBF (414 nm) and ADBA (360, 380, and 400 nm). 630 nm (4.0 mW/cm^2^) for DCM and 660 nm (25 mW/cm^2^) for PBS LED bulbs were used as light sources. The light sources were exposed from a 5 cm cell distance, and absorbances were taken at intervals for each solution of PSs. Equation 2 was used to calculate the ^1^O_2_ quantum yields where dyad and ref refer to “NI-BODIPY and NI-BODIPY-GO” and “MB”, respectively. *k* is the slope of difference in change in absorbance of DPBF (414 nm) with irradiation time. *F* is the absorption correction factor, which is given by *F* = 1–10^–OD^ (OD is the absorption at the irradiation wavelength), and PF is light intensity (energy flux, mW/ cm^2^), which was used to calculate ^1^O_2_ quantum yields.
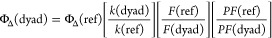
2

### Cell Culture

K562 human chronic myelogenous leukemia suspension cells were cultured in 25 cm^2^ culture flasks with complete Dulbecco’s modified Eagle’s medium (DMEM, supplemented with 2 mM l-glutamine, 20% fetal bovine serum, 100 units/mL penicillin, and 100 μg/mL of streptomycin) under environmental conditions of 37 °C, 5% CO_2_, and 60% humidity. Cells were exposed to varying concentrations of the chemicals and irradiated with a LED source (660 nm).

### MTT Assay Studies

Cytotoxicity of the chemicals was investigated via MTT assay. Briefly, 50 μL cell suspensions in complete DMEM containing 1 × 10^4^ K562 cells were plated in 96-well flat-bottom culture plates (Corning, Massachusetts, USA) and incubated for 12 h to recover from handling. Varying concentrations of compounds **10–12** and **GO-10-12** in complete DMEM were added into each well. The experimental group of the cells was illuminated with an LED source (660 nm) for 8 h in a culture incubator (37 °C, 5% CO_2_, 60% humidity). This 8 h period of irradiation was followed by a 40 h period of incubation in the dark (total 48 h) also in the incubator. The control group of the cells was incubated in the dark, for a duration of 48 h under identical environmental conditions except irradiation. According to the assay protocol, 25 μL of the MTT reagent (Sigma-Aldrich, Missouri, USA) was added to each well in order to assess cell viability (final concentration: 1 mg/mL) at the end of the 48 h incubation period. Following a 4 h incubation of the cells with the MTT reagent, the generated formazan precipitates were solubilized by addition of the lysing buffer (80 μL, pH: 4.7), which is composed of 23% SDS (sodium dodecyl sulfate) dissolved in a solution of 45% DMF. After an overnight incubation at 37 °C, the absorbance values (of each well) were measured at 570 nm in a microtiter plate reader (SpectraMax Plus, Molecular Devices, California, USA) at 25 °C. Cells incubated in culture medium only (without any drug) served as the control for cell viability both for the illuminated plates and for the ones kept in the dark. Normalized cell number (%) was calculated with normalization of the values. The IC_50_ values of the chemicals were estimated by fitting a model with nonlinear regression.

## Supporting Information Available

Analyses of the compounds (**10**–**12**) and nanocomposites [**GO**-(**10**–**12**)]; MALDI-TOF mass, FTIR, ^1^H NMR, ^13^C NMR, Raman, UV–vis absorption, and fluorescence emission spectra; TEM, EDX, and AFM images; UV–vis spectral change due to singlet oxygen generation (PDF)

## References

[ref1] Wojtoniszak M.; Rogińska D.; Machaliński B.; Drozdzik M.; Mijowska E. Graphene Oxide Functionalized with Methylene Blue and Its Performance in Singlet Oxygen Generation. Mater. Res. Bull. 2013, 48, 2636–2639. 10.1016/j.materresbull.2013.03.040.

[ref2] Tian B.; Wang C.; Zhang S.; Feng L.; Liu Z. Photothermally Enhanced Photodynamic Therapy Delivered by Nano-Graphene Oxide. ACS Nano 2011, 5, 7000–7009. 10.1021/nn201560b. 21815655

[ref3] Turan I. S.; Yildiz D.; Turksoy A.; Gunaydin G.; Akkaya E. U. A Bifunctional Photosensitizer for Enhanced Fractional Photodynamic Therapy: Singlet Oxygen Generation in the Presence and Absence of Light. Angew. Chem., Int. Ed. 2016, 55, 2875–2878. 10.1002/anie.201511345. 26799149

[ref4] Gunaydin G.; Gedik M. E.; Ayan S. Photodynamic Therapy—Current Limitations and Novel Approaches. Front. Chem. 2021, 9, 691697 10.3389/fchem.2021.691697. 34178948 PMC8223074

[ref5] Yan L.; Chang Y.-N.; Yin W.; Tian G.; Zhou L.; Liu X.; Xing G.; Zhao L.; Gu Z.; Zhao Y. On-Demand Generation of Singlet Oxygen from a Smart Graphene Complex for the Photodynamic Treatment of Cancer Cells. Biomater. Sci. 2014, 2, 1412–1418. 10.1039/C4BM00143E. 32481917

[ref6] Zhou L.; Wang W.; Tang J.; Zhou J. H.; Jiang H. J.; Shen J. Graphene Oxide Noncovalent Photosensitizer and Its Anticancer Activity in Vitro. Chem. – Eur. J. 2011, 17, 12084–12091. 10.1002/chem.201003078. 21915922

[ref7] Conti L.; Macedi E.; Giorgi C.; Valtancoli B.; Fusi V. Combination of Light and Ru(II) Polypyridyl Complexes: Recent Advances in the Development of New Anticancer Drugs. Coord. Chem. Rev. 2022, 469, 214656 10.1016/j.ccr.2022.214656.

[ref8] Takemura T.; Ohta N.; Nakajima S.; Sakata I. Critical Importance Of The Triplet Lifetime Of Photosensitizer In Photodynamic Therapy Of Tumor. Photochem. Photobiol. 1989, 50, 339–344. 10.1111/j.1751-1097.1989.tb04167.x. 2780823

[ref9] Park H.; Na K. Conjugation of the Photosensitizer Chlorin E6 to Pluronic F127 for Enhanced Cellular Internalization for Photodynamic Therapy. Biomaterials 2013, 34, 6992–7000. 10.1016/j.biomaterials.2013.05.070. 23777915

[ref10] Maree M. D.; Kuznetsova N.; Nyokong T. Silicon Octaphenoxyphthalocyanines: Photostability and Singlet Oxygen Quantum Yields. J. Photochem. Photobiol., A 2001, 140, 117–125. 10.1016/S1010-6030(01)00409-9.

[ref11] Ferrari M. Cancer Nanotechnology: Opportunities and Challenges. Nat. Rev. Cancer 2005, 161–171. 10.1038/nrc1566. 15738981

[ref12] Zhu Z.; Tang Z.; Phillips J. A.; Yang R.; Wang H.; Tan W. Regulation of Singlet Oxygen Generation Using Single-Walled Carbon Nanotubes. J. Am. Chem. Soc. 2008, 130, 10856–10857. 10.1021/ja802913f. 18661988

[ref13] Feng L.; Wu L.; Qu X. New Horizons for Diagnostics and Therapeutic Applications of Graphene and Graphene Oxide. Adv. Mater. 2013, 25, 168–186. 10.1002/adma.201203229. 23161646

[ref14] Zhang H.; Ma Y.; Sun X.-L. Recent Developments in Carbohydrate-Decorated Targeted Drug/Gene Delivery. Med. Res. Rev. 2010, 30, 270–289. 10.1002/med.20171. 19626595

[ref15] Kang B.; Opatz T.; Landfester K.; Wurm F. R. Carbohydrate Nanocarriers in Biomedical Applications: Functionalization and Construction. Chem. Soc. Rev. 2015, 44, 8301–8325. 10.1039/C5CS00092K. 26278884

[ref16] Treekoon J.; Pewklang T.; Chansaenpak K.; Gorantla J. N.; Pengthaisong S.; Lai R.-Y.; Ketudat-Cairns J. R.; Kamkaew A. Glucose Conjugated Aza-BODIPY for Enhanced Photodynamic Cancer Therapy. Org. Biomol. Chem. 2021, 19, 5867–5875. 10.1039/D1OB00400J. 34124730

[ref17] Wang J.; Zhang Y.; Lu Q.; Xing D.; Zhang R. Exploring Carbohydrates for Therapeutics: A Review on Future Directions. Front. Pharmacol. 2021, 12, 1–9. 10.3389/fphar.2021.756724. PMC8634948 34867374

[ref18] Yu X.-T.; Sui S.-Y.; He Y.-X.; Yu C.-H.; Peng Q. Nanomaterials-Based Photosensitizers and Delivery Systems for Photodynamic Cancer Therapy. Biomater. Adv. 2022, 135, 212725 10.1016/j.bioadv.2022.212725. 35929205

[ref19] Işıklan N.; Hussien N. A.; Türk M. Multifunctional Aptamer-Conjugated Magnetite Graphene Oxide/Chlorin E6 Nanocomposite for Combined Chemo-Phototherapy. Colloids Surf., A 2022, 645, 128841 10.1016/j.colsurfa.2022.128841.

[ref20] Yang X.; Wang Y.; Huang X.; Ma Y.; Huang Y.; Yang R.; Duan H.; Chen Y. Multi-Functionalized Graphene Oxide Based Anticancer Drug-Carrier with Dual-Targeting Function and PH-Sensitivity. J. Mater. Chem. 2011, 21, 3448–3454. 10.1039/C0JM02494E.

[ref21] Hu Z.; Li J.; Li C.; Zhao S.; Li N.; Wang Y.; Wei F.; Chen L.; Huang Y. Folic Acid-Conjugated Graphene–ZnO Nanohybrid for Targeting Photodynamic Therapy under Visible Light Irradiation. J. Mater. Chem. B 2013, 1, 5003–5013. 10.1039/c3tb20849d. 32261090

[ref22] Li F.; Park S. J.; Ling D.; Park W.; Han J. Y.; Na K.; Char K. Hyaluronic Acid-Conjugated Graphene Oxide/Photosensitizer Nanohybrids for Cancer Targeted Photodynamic Therapy. J. Mater. Chem. B 2013, 1, 1678–1686. 10.1039/c3tb00506b. 32260699

[ref23] Qin X.; Zhang H.; Wang Z.; Jin Y. Magnetic Chitosan/Graphene Oxide Composite Loaded with Novel Photosensitizer for Enhanced Photodynamic Therapy. RSC Adv. 2018, 8, 10376–10388. 10.1039/C8RA00747K. 35540483 PMC9078887

[ref24] Bai L.-Z.; Zhao D.-L.; Xu Y.; Zhang J.-M.; Gao Y.-L.; Zhao L.-Y.; Tang J.-T. Inductive Heating Property of Graphene Oxide–Fe3O4 Nanoparticles Hybrid in an AC Magnetic Field for Localized Hyperthermia. Mater. Lett. 2012, 68, 399–401. 10.1016/j.matlet.2011.11.013.

[ref25] Yang K.; Hu L.; Ma X.; Ye S.; Cheng L.; Shi X.; Li C.; Li Y.; Liu Z. Multimodal Imaging Guided Photothermal Therapy Using Functionalized Graphene Nanosheets Anchored with Magnetic Nanoparticles. Adv. Mater. 2012, 24, 1868–1872. 10.1002/adma.201104964. 22378564

[ref26] Santos C. M.; Tria M. C. R.; Vergara R. A. M. V.; Ahmed F.; Advincula R. C.; Rodrigues D. F. Antimicrobial Graphene Polymer (PVK-GO) Nanocomposite Films. Chem. Commun. 2011, 47, 8892–8894. 10.1039/c1cc11877c. 21670830

[ref27] Zhang L.; Xia J.; Zhao Q.; Liu L.; Zhang Z. Functional Graphene Oxide as a Nanocarrier for Controlled Loading and Targeted Delivery of Mixed Anticancer Drugs. Small 2010, 6, 537–544. 10.1002/smll.200901680. 20033930

[ref28] Mai D. K.; Kim C.; Lee J.; Vales T. P.; Badon I. W.; De K.; Cho S.; Yang J.; Kim H.-J. BODIPY Nanoparticles Functionalized with Lactose for Cancer-Targeted and Fluorescence Imaging-Guided Photodynamic Therapy. Sci. Rep. 2022, 12, 2541 10.1038/s41598-022-06000-5. 35169149 PMC8847361

[ref29] Wang J.; Gong Q.; Wang L.; Hao E.; Jiao L. The Main Strategies for Tuning BODIPY Fluorophores into Photosensitizers. J. Porphyr. Phthalocyanines 2020, 24, 603–635. 10.1142/S1088424619300234.

[ref30] Chen K.; Dong Y.; Zhao X.; Imran M.; Tang G.; Zhao J.; Liu Q. Bodipy Derivatives as Triplet Photosensitizers and the Related Intersystem Crossing Mechanisms. Front. Chem. 2019, 7, 1–14. 10.3389/fchem.2019.00821. 31921760 PMC6920128

[ref31] Nguyen V.-N.; Ha J.; Cho M.; Li H.; Swamy K. M. K.; Yoon J. Recent Developments OfBODIPY-Based Colorimetric and Fluorescent Probes for the Detection of Reactive Oxygen/Nitrogen Species and Cancer Diagnosis. Coord. Chem. Rev. 2021, 439, 213936 10.1016/j.ccr.2021.213936.

[ref32] Zhang T.; Ma C.; Sun T.; Xie Z. Unadulterated BODIPY Nanoparticles for Biomedical Applications. Coord. Chem. Rev. 2019, 390, 76–85. 10.1016/j.ccr.2019.04.001.

[ref33] Sharker S. M.; Jeong C. J.; Kim S. M.; Lee J.-E.; Jeong J. H.; In I.; Lee H.; Park S. Y. Photo- and PH-Tunable Multicolor Fluorescent Nanoparticle-Based Spiropyran- and BODIPY-Conjugated Polymer with Graphene Oxide. Chem. – Asian J. 2014, 9, 2921–2927. 10.1002/asia.201402399. 25056486

[ref34] Lu S.; Lei X.; Ren H.; Zheng S.; Qiang J.; Zhang Z.; Chen Y.; Wei T.; Wang F.; Chen X. PEGylated Dimeric BODIPY Photosensitizers as Nanocarriers for Combined Chemotherapy and Cathepsin B-Activated Photodynamic Therapy in 3D Tumor Spheroids. ACS Appl. Bio Mater. 2020, 3, 3835–3845. 10.1021/acsabm.0c00394. 35025254

[ref35] Chen H.; Bi Q.; Yao Y.; Tan N. Dimeric BODIPY-Loaded Liposomes for Dual Hypoxia Marker Imaging and Activatable Photodynamic Therapy against Tumors. J. Mater. Chem. B 2018, 6, 4351–4359. 10.1039/C8TB00665B. 32254510

[ref36] Üçüncü M.; Karaksu̧ E.; Kurulgan Demirci E.; Sayar M.; Dartar S.; Emrullahoğlu M. BODIPY–Au(I): A Photosensitizer for Singlet Oxygen Generation and Photodynamic Therapy. Org. Lett. 2017, 19, 2522–2525. 10.1021/acs.orglett.7b00791. 28485948

[ref37] Bassan E.; Gualandi A.; Cozzi P. G.; Ceroni P. Design of BODIPY Dyes as Triplet Photosensitizers: Electronic Properties Tailored for Solar Energy Conversion{,} Photoredox Catalysis and Photodynamic Therapy. Chem. Sci. 2021, 12, 6607–6628. 10.1039/D1SC00732G. 34040736 PMC8132938

[ref38] Xu Z.; Zhou Y.; Wang J.; Mao L.; Li W.; Xu G. The Synthesis and Antitumor Activity of 1,8-Naphthalimide Derivatives Linked 1,2,3-Triazole. Front. Bioeng. Biotechnol. 2021, 9, 339 10.3389/fbioe.2021.662432. PMC8076741 33928073

[ref39] Dong H.-Q.; Wei T.-B.; Ma X.-Q.; Yang Q.-Y.; Zhang Y.-F.; Sun Y.-J.; Shi B.-B.; Yao H.; Zhang Y.-M.; Lin Q. 1{,}8-Naphthalimide-Based Fluorescent Chemosensors: Recent Advances and Perspectives. J. Mater. Chem. C 2020, 8, 13501–13529. 10.1039/D0TC03681A.

[ref40] Banerjee S.; Veale E. B.; Phelan C. M.; Murphy S. A.; Tocci G. M.; Gillespie L. J.; Frimannsson D. O.; Kelly J. M.; Gunnlaugsson T. Recent Advances in the Development of 1{,}8-Naphthalimide Based DNA Targeting Binders{,} Anticancer and Fluorescent Cellular Imaging Agents. Chem. Soc. Rev. 2013, 42, 1601–1618. 10.1039/c2cs35467e. 23325367

[ref41] Lv M.; Xu H. Overview of Naphthalimide Analogs as Anticancer Agents. Curr. Med. Chem. 2009, 16, 4797–4813. 10.2174/092986709789909576. 19929786

[ref42] Liu J.; Cui L.; Losic D. Graphene and Graphene Oxide as New Nanocarriers for Drug Delivery Applications. Acta Biomater. 2013, 9, 9243–9257. 10.1016/j.actbio.2013.08.016. 23958782

[ref43] Xu X.-L.; Shao J.; Chen Q.-Y.; Li C.-H.; Kong M.-Y.; Fang F.; Ji L.; Boison D.; Huang T.; Gao J.; Feng C.-J. A Mn(II) Complex of Boradiazaindacene (BODIPY) Loaded Graphene Oxide as Both LED Light and H2O2 Enhanced Anticancer Agent. J. Inorg. Biochem. 2016, 159, 1–6. 10.1016/j.jinorgbio.2016.02.007. 26901626

[ref44] Su Y.; Wang N.; Liu B.; Du Y.; Li R.; Meng Y.; Feng Y.; Shan Z.; Meng S. A Phototheranostic Nanoparticle for Cancer Therapy Fabricated by BODIPY and Graphene to Realize Photo-Chemo Synergistic Therapy and Fluorescence/Photothermal Imaging. Dyes Pigm. 2020, 177, 108262 10.1016/j.dyepig.2020.108262.

[ref45] Eserci H.; Çetin M.; Aydınoğlu F.; Eçik E. T.; Okutan E. Naphthalimide-BODIPY Dyads: Synthesis, Characterization, Photophysical Properties, Live Cell Imaging and Antimicrobial Effect. J. Mol. Struct. 2022, 1265, 133440 10.1016/j.molstruc.2022.133440.

[ref46] Rani K.; Chawla S.; Kumari V.; De A. K.; Sengupta S. Unravelling the Excited State Dynamics of Monofunctionalized Mono- and Distyryl-BODIPY and Perylenediimide Dyads. J. Mater. Chem. C 2022, 10, 10551–10561. 10.1039/D2TC01741E.

[ref47] Pimenta M. A.; Dresselhaus G.; Dresselhaus M. S.; Cançado L. G.; Jorio A.; Saito R. Studying Disorder in Graphite-Based Systems by Raman Spectroscopy. Phys. Chem. Chem. Phys. 2007, 9, 1276–1290. 10.1039/B613962K. 17347700

[ref48] Zhou W.; Liu Y.; Liu G.; Niu X.; Niu X.; Li X.; Feng G.; Zhang Y.; Xing G. Water-Soluble Meso-Ester Substituted BODIPY with Aggregation-Induced Emission Property for Ratiometric Detection of Carboxylesterases in Living Hepatoma Cell. Dyes Pigm. 2022, 201, 110189 10.1016/j.dyepig.2022.110189.

[ref49] Gündüz E. Ö.; Gedik M. E.; Günaydın G.; Okutan E. Amphiphilic Fullerene-BODIPY Photosensitizers for Targeted Photodynamic Therapy. ChemMedChem 2022, 17, e202100693 10.1002/cmdc.202100693. 34859597

[ref50] Morales-Narváez E.; Merkoçi A. Graphene Oxide as an Optical Biosensing Platform. Adv. Mater. 2012, 24, 3298–3308. 10.1002/adma.201200373. 22628274

[ref51] Wang X.; Wang C.; Qu K.; Song Y.; Ren J.; Miyoshi D.; Sugimoto N.; Qu X. Ultrasensitive and Selective Detection of a Prognostic Indicator in Early-Stage Cancer Using Graphene Oxide and Carbon Nanotubes. Adv. Funct. Mater. 2010, 20, 3967–3971. 10.1002/adfm.201001118.

[ref52] Wu X.; Xing Y.; Zeng K.; Huber K.; Zhao J. X. Study of Fluorescence Quenching Ability of Graphene Oxide with a Layer of Rigid and Tunable Silica Spacer. Langmuir 2018, 34, 603–611. 10.1021/acs.langmuir.7b03465. 29275632

[ref53] Sarıkaya S. Y.; Yeşilot S.; Kılıç A.; Okutan E. Novel BODIPY-Cyclotriphosphazene- Fullerene Triads: Synthesis, Characterization and Singlet Oxygen Generation Efficiency. Dyes Pigm. 2018, 153, 26–34. 10.1016/j.dyepig.2018.02.001.

[ref54] Brouwer A. M. Standards for Photoluminescence Quantum Yield Measurements in Solution (IUPAC Technical Report). Pure Appl. Chem. 2011, 2213–2228. 10.1351/PAC-REP-10-09-31.

[ref55] Ogunsipe A.; Chen J.-Y.; Nyokong T. Photophysical and Photochemical Studies of Zinc(Ii) Phthalocyanine Derivatives—Effects of Substituents and Solvents. New J. Chem. 2004, 28, 822–827. 10.1039/B315319C.

