# Supplementary material for: NI-BODIPY-GO Nanocomposites for Targeted PDT
Source: ACS Omega. 2023 Feb 22;8(9):8320–31. doi: 10.1021/acsomega.2c06900 (PMC9996583; doi:10.1021/acsomega.2c06900)
Supplement: Supplementary file 1 — ao2c06900_si_001.pdf [file ao2c06900_si_001.pdf]

Supporting Information

NI-BODIPY-GO Nanocomposites for Targeted PDT

Ezel ÖZTÜRK GÜNDÜZ[a], Berkan TASASIZ[a], M. Emre GEDİK[b], Gürcan GÜNAYDIN[b], Elif OKUTAN[a*]

[a] Department of Chemistry, Faculty of Science, Gebze Technical University, Gebze, Kocaeli, Turkey

[b] Department of Basic Oncology, Cancer Institute, Hacettepe University, Çankaya, Ankara, Turkey

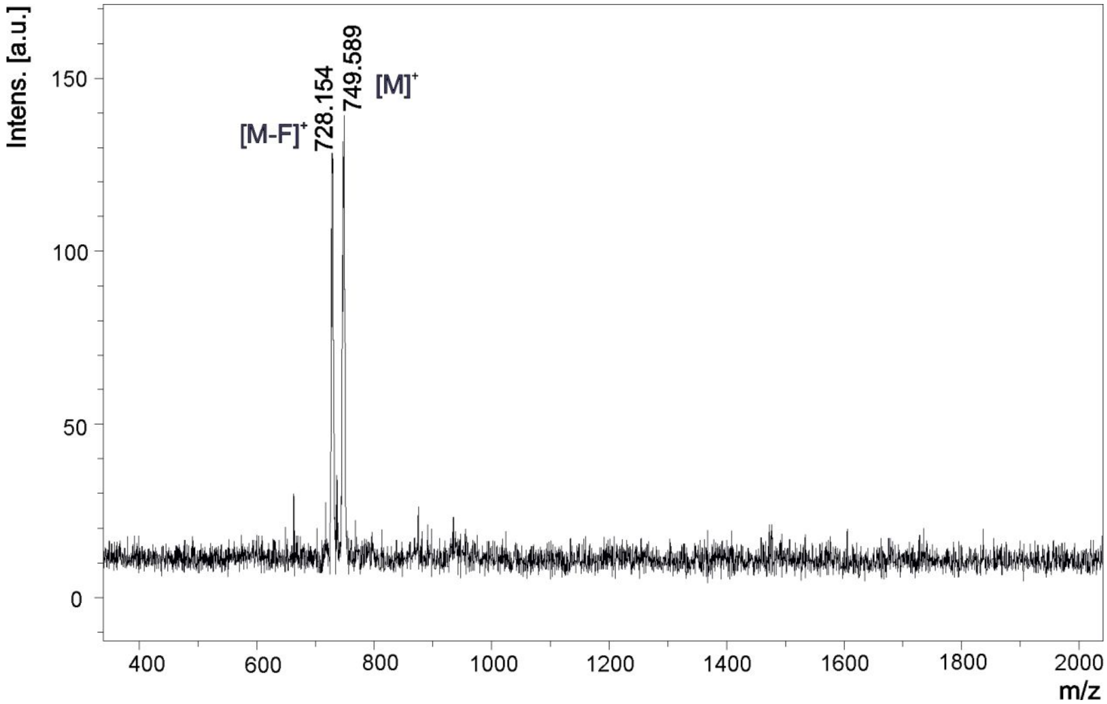

Figure S1. MALDI-MS spectrum of compound **5**

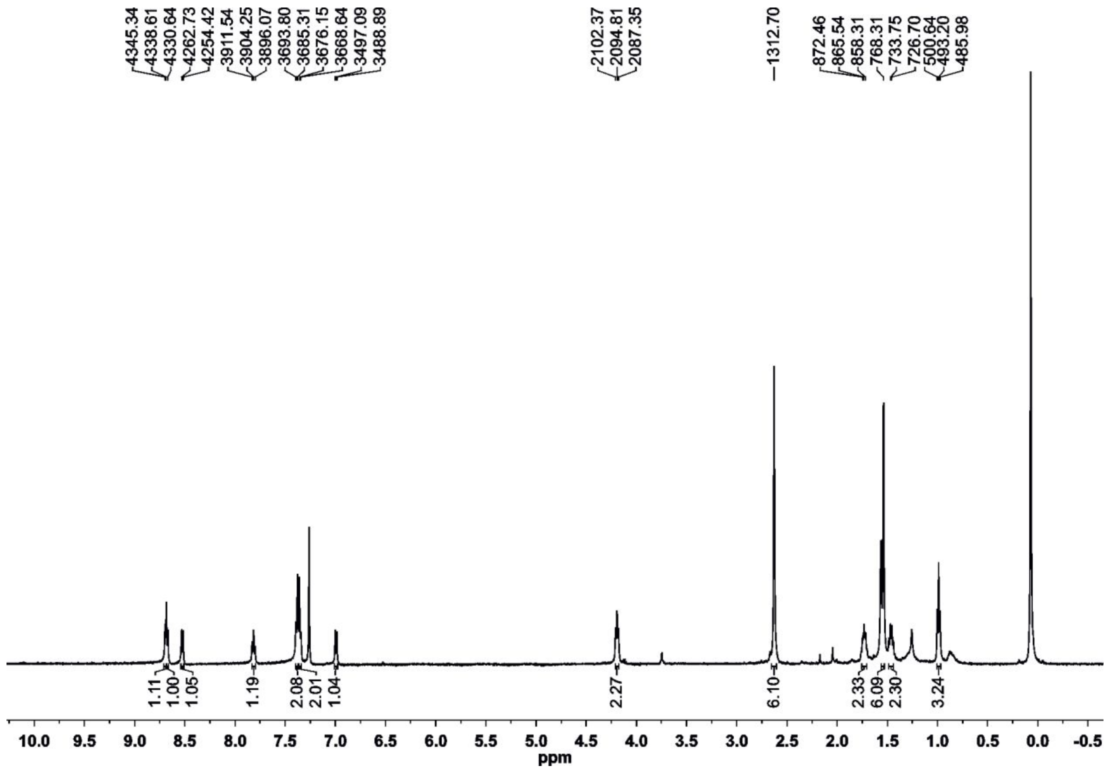

Figure S2. ^{1}H NMR spectrum of compound **5** in $CDCl_3$

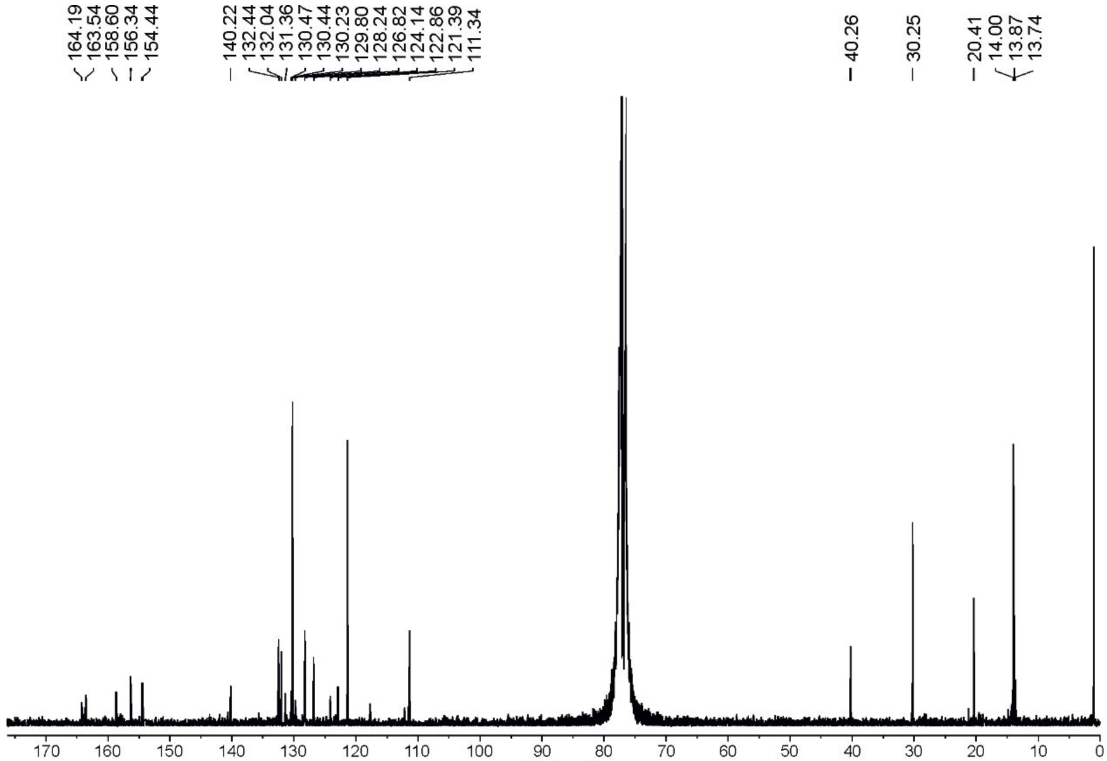

Figure S3. ^{13}C NMR spectrum of compound **5** in $CDCl_3$

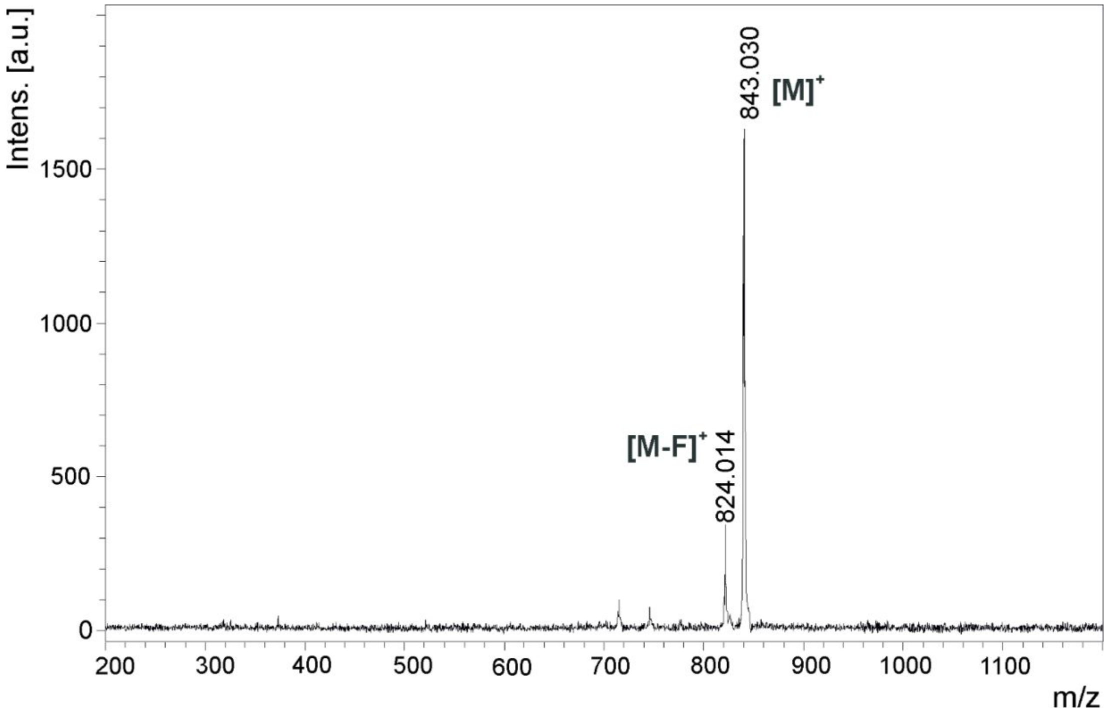

Figure S4. MALDI-MS spectrum of compound **6**

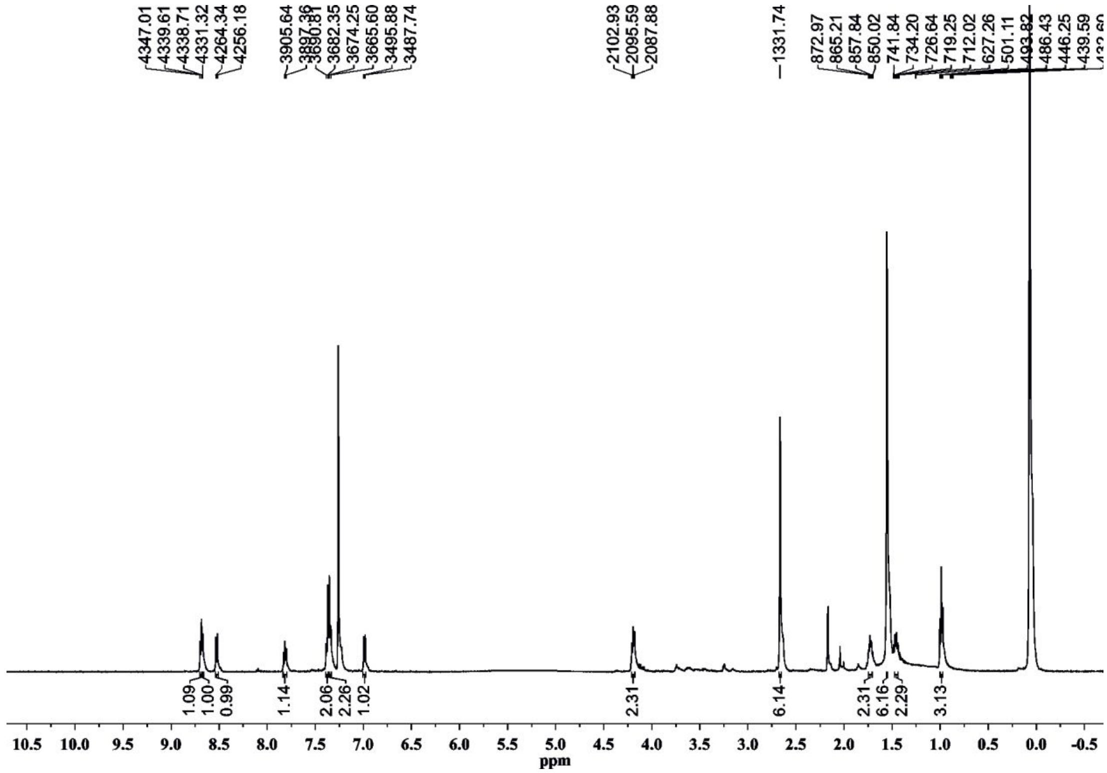

Figure S5. ^{1}H NMR spectrum of compound **6** in $CDCl_3$

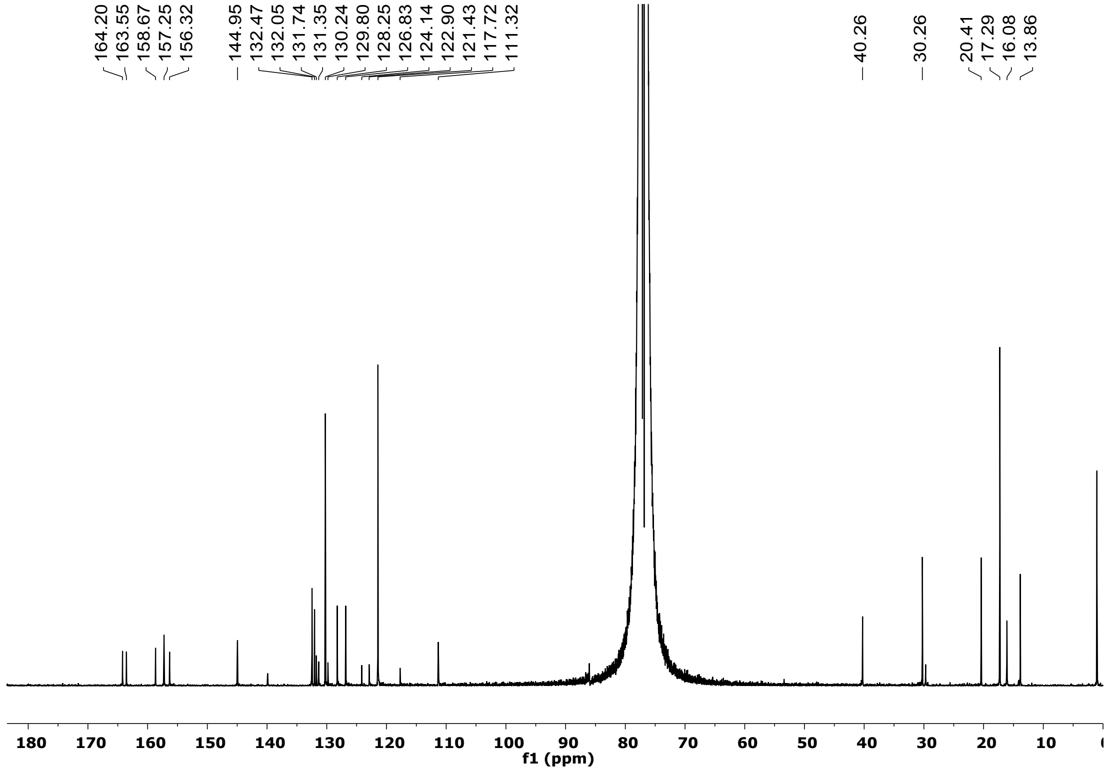

Figure S6. ^{13}C NMR spectrum of compound **6** in $CDCl_3$

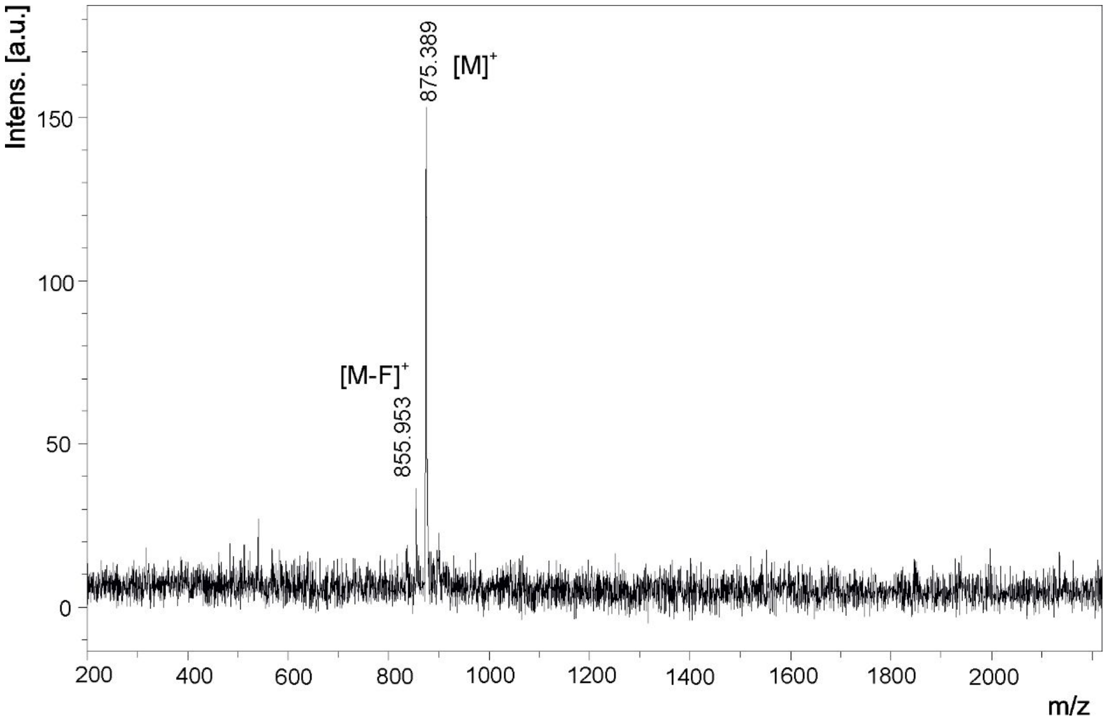

Figure S7. MALDI-MS spectrum of compound **7**

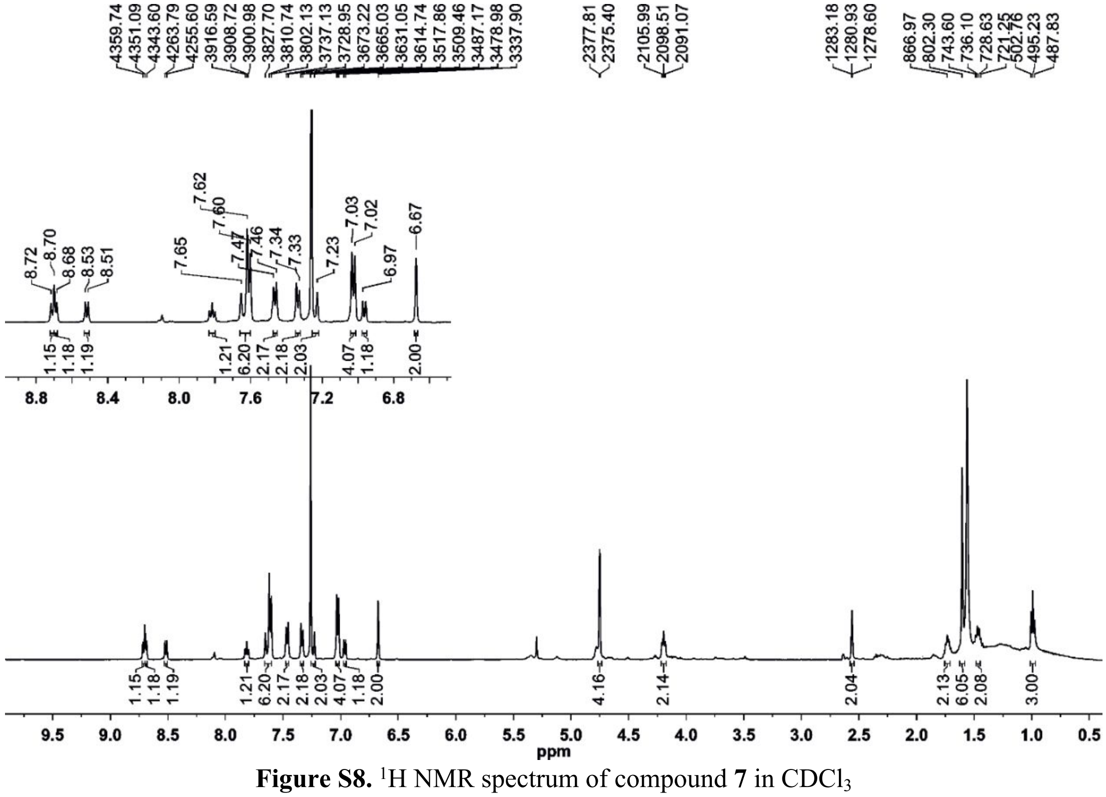

Figure S8. ^{1}H NMR spectrum of compound **7** in $CDCl_3$

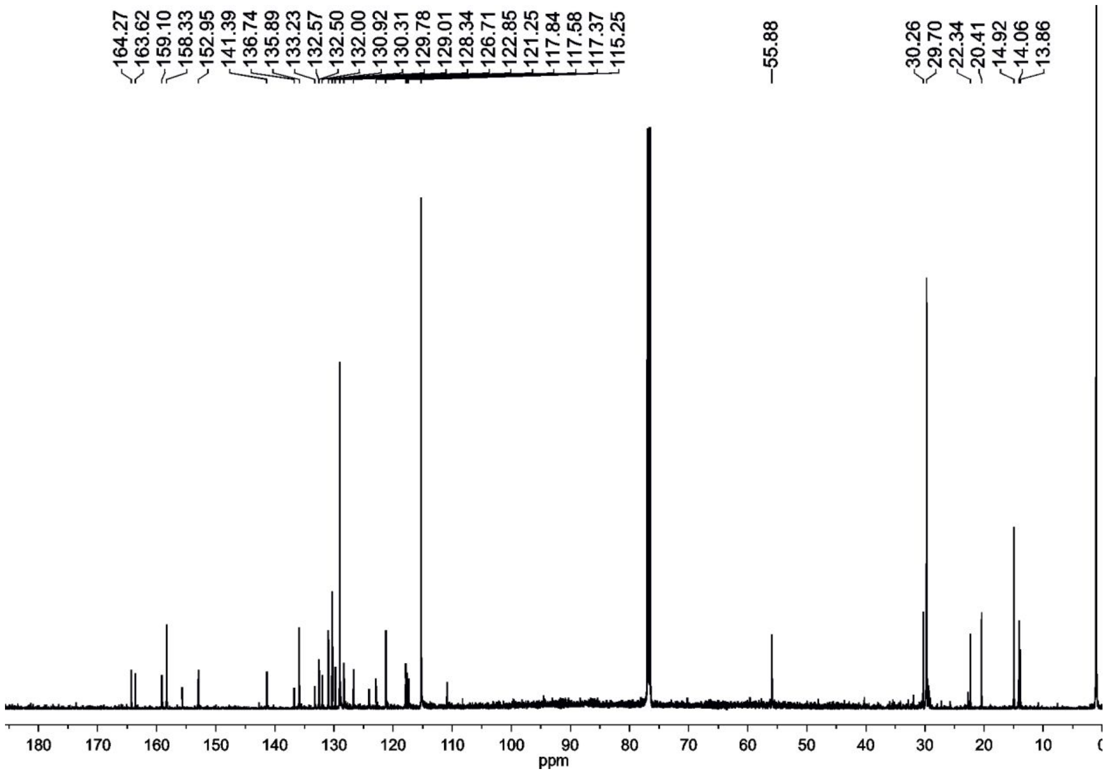

Figure S9. ^{13}C NMR spectrum of compound **7** in $CDCl_3$

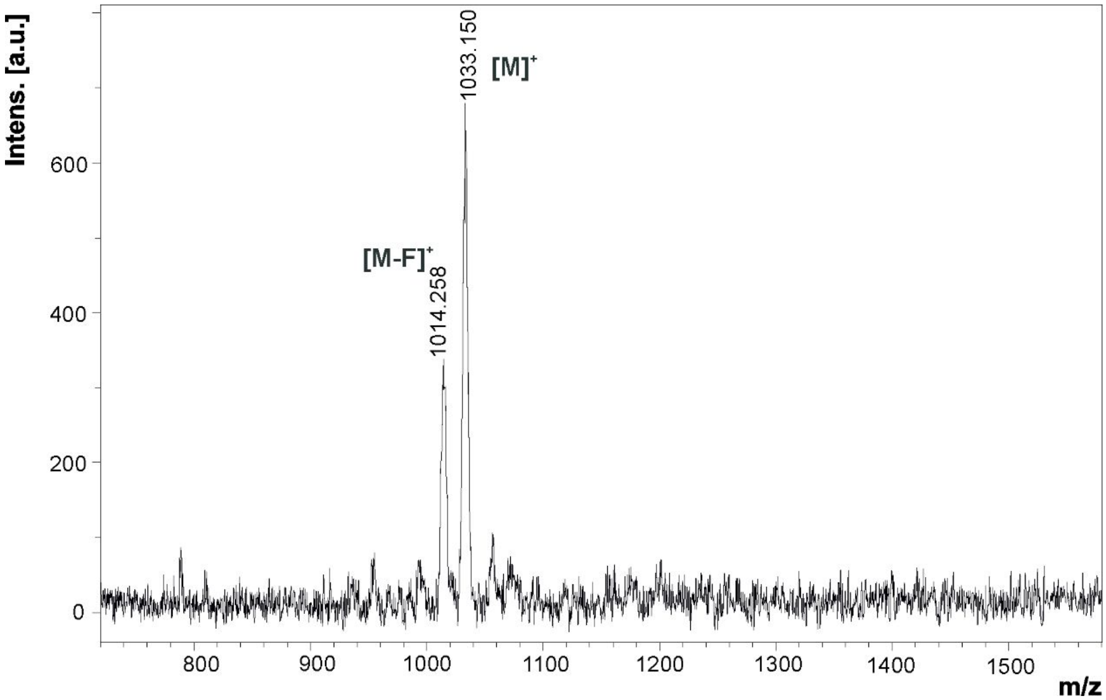

Figure S10. MALDI-MS spectrum of compound **8**

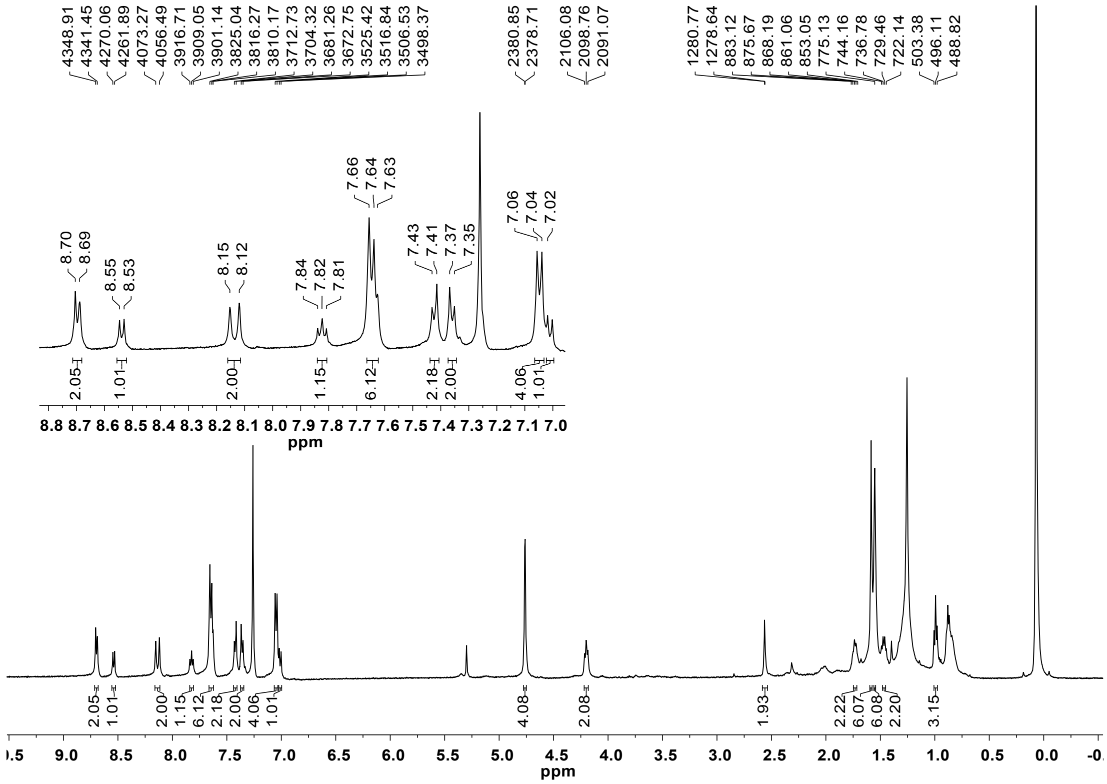

Figure S11. ^{1}H NMR spectrum of compound **8** in $CDCl_3$

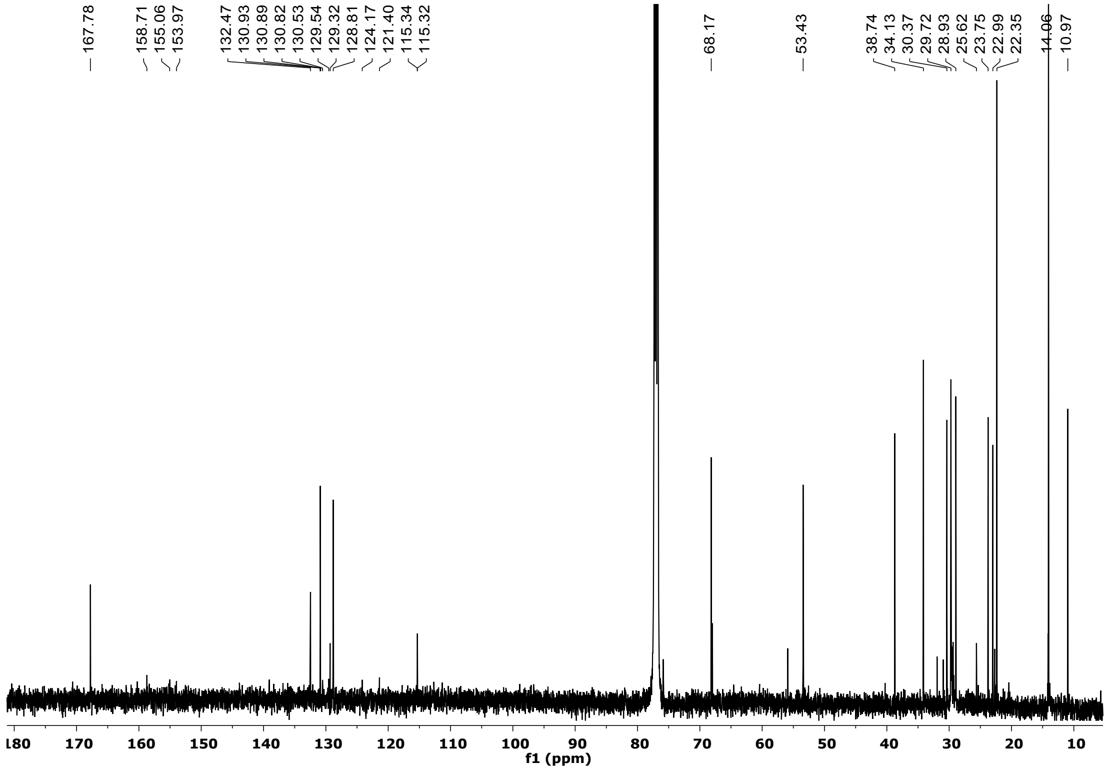

Figure S12. ^{13}C NMR spectrum of compound **8** in $CDCl_3$

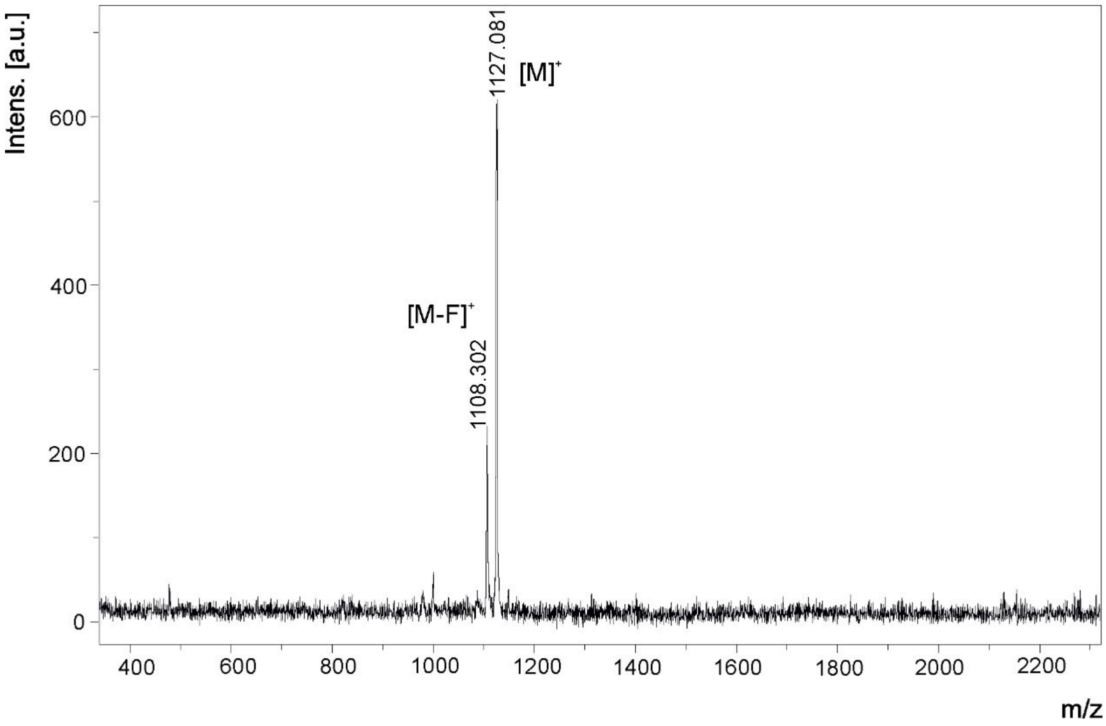

Figure S7. MALDI-MS spectrum of compound **9**

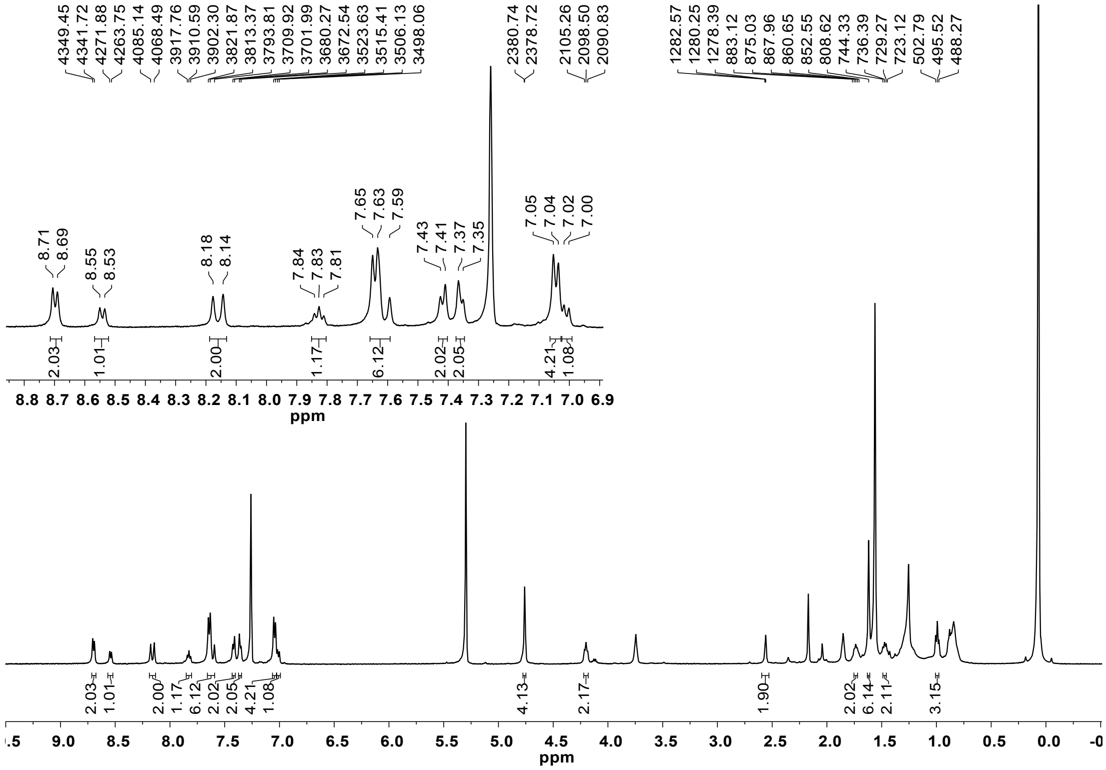

Figure S14. ^{1}H NMR spectrum of compound **9** in $CDCl_3$

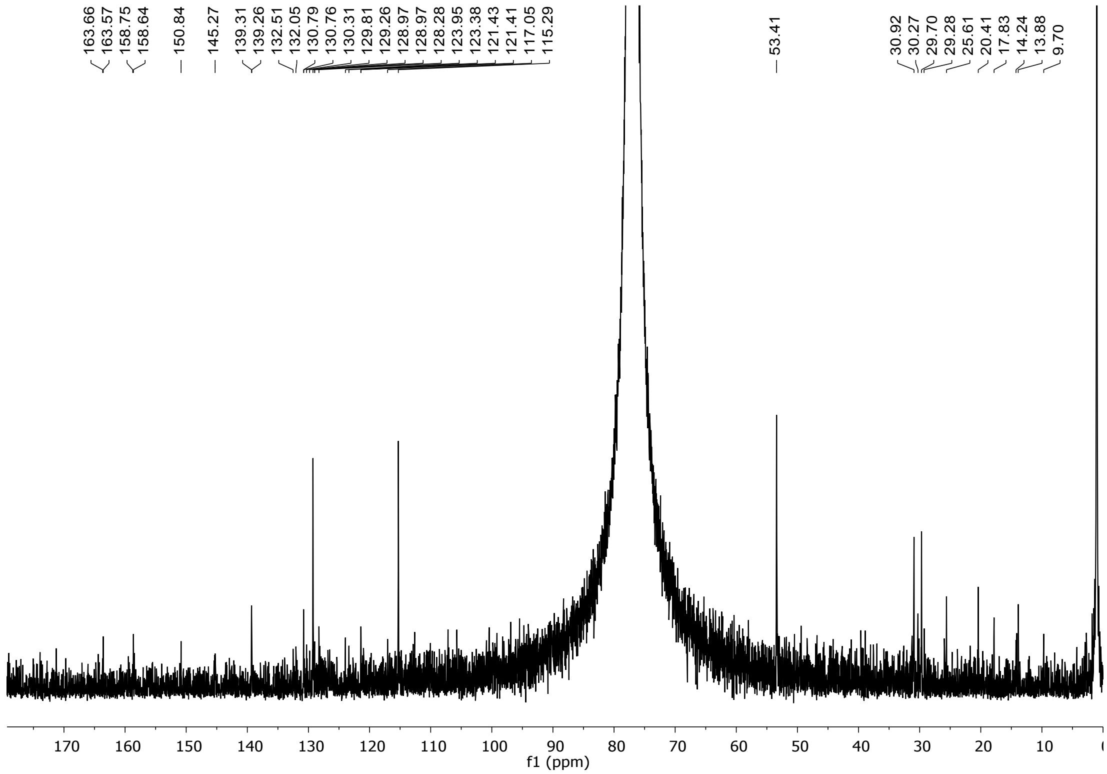

Figure S8. ^{13}C NMR spectrum of compound **9** in $CDCl_3$

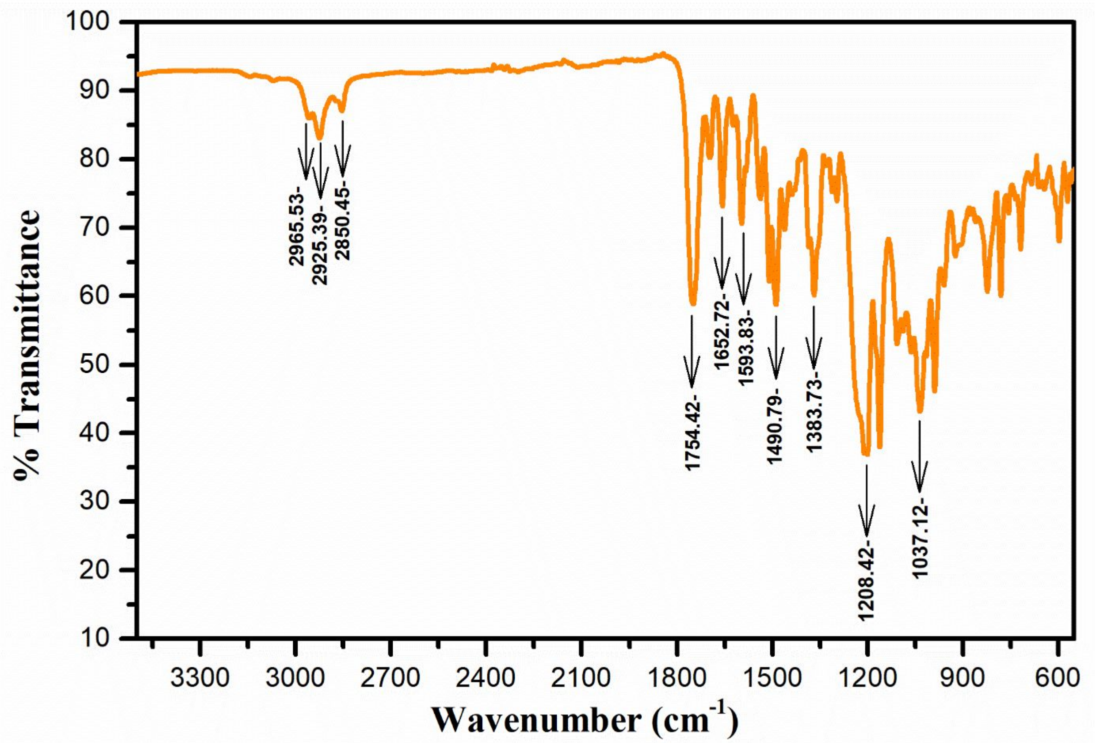

Figure S9. FT-IR spectrum of compound **10**

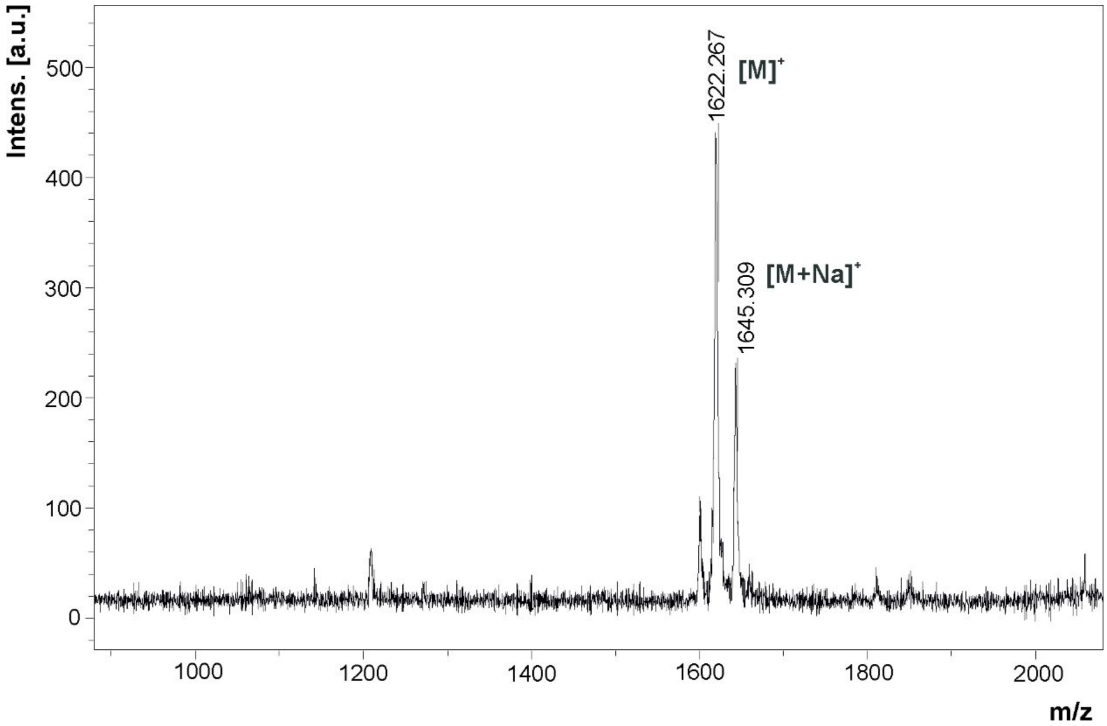

Figure S10. MALDI-MS spectrum of compound **10**

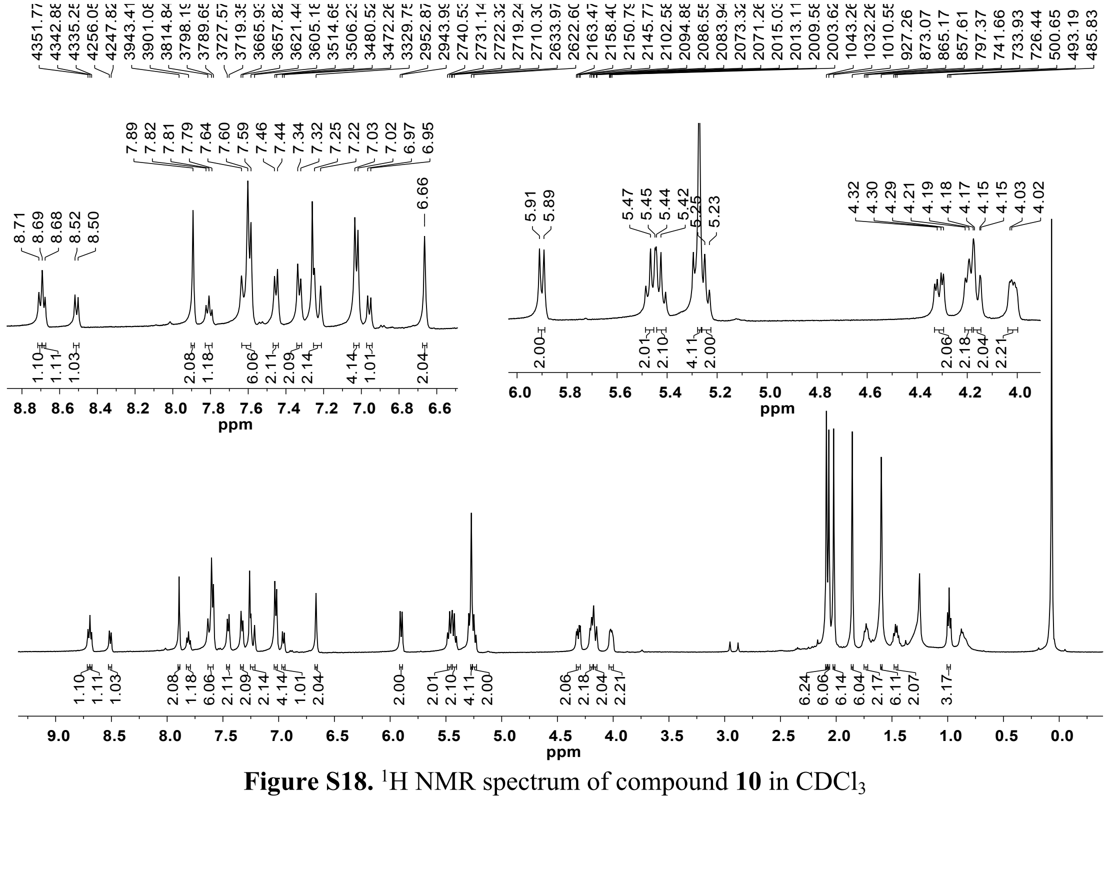

Figure S18. ^{1}H NMR spectrum of compound **10** in $CDCl_3$

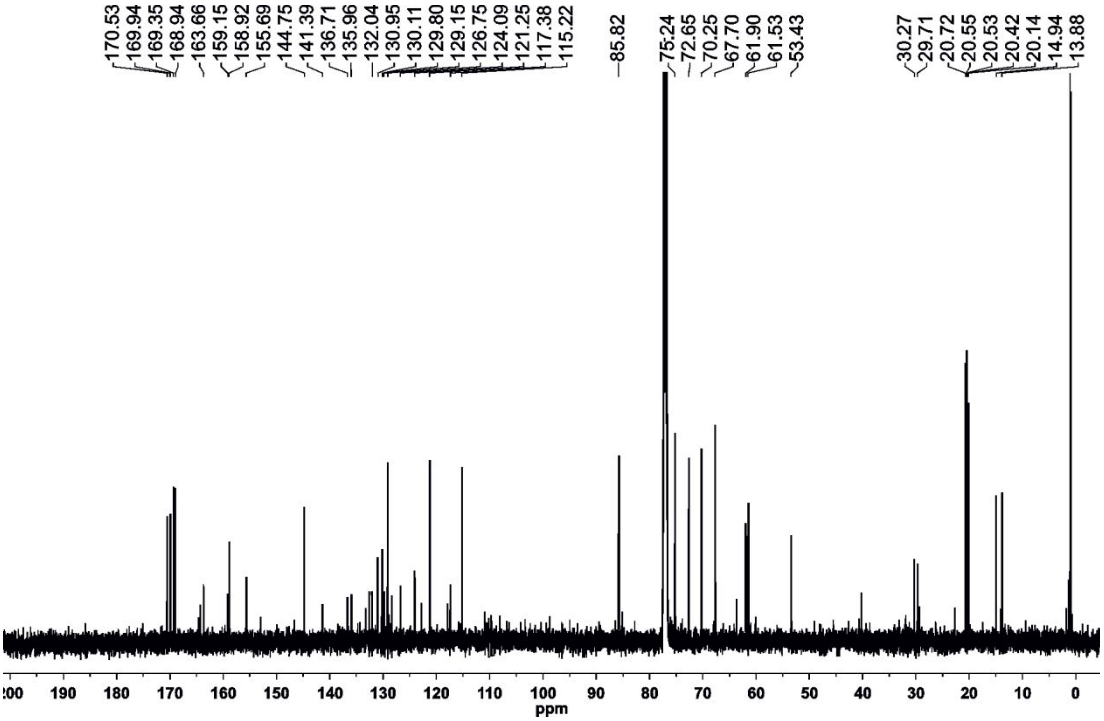

Figure S11. ^{13}C NMR spectrum of compound **10** in $CDCl_3$

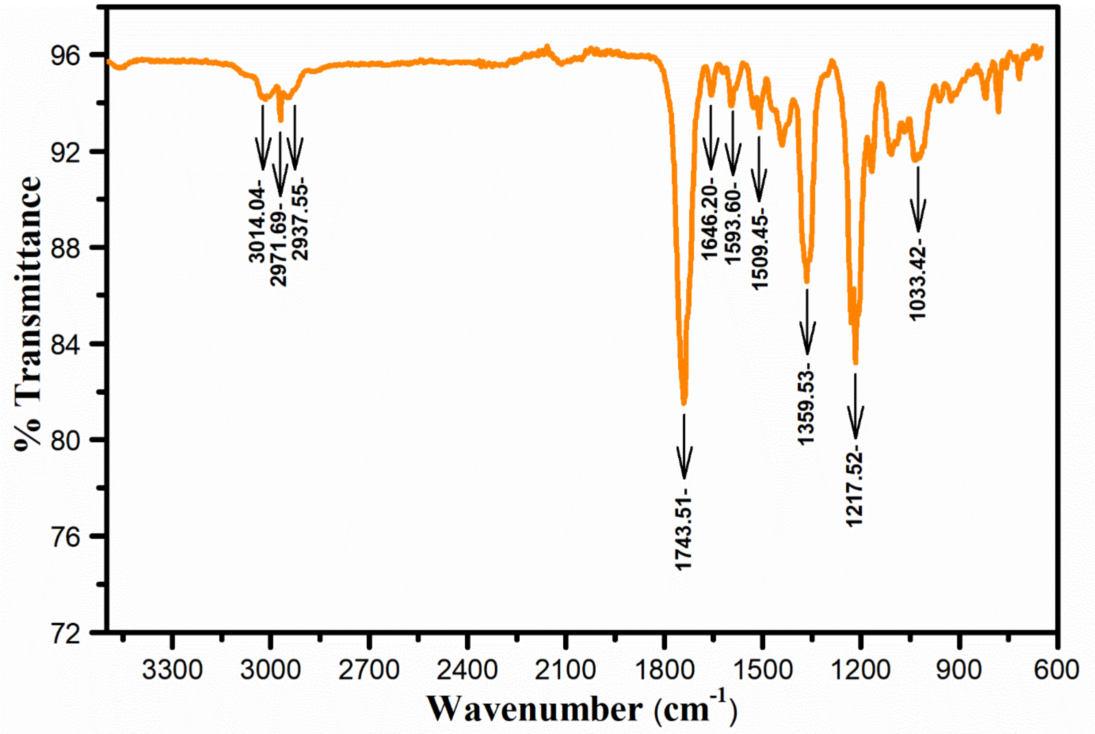

Figure S20. FT-IR spectrum of compound **11**

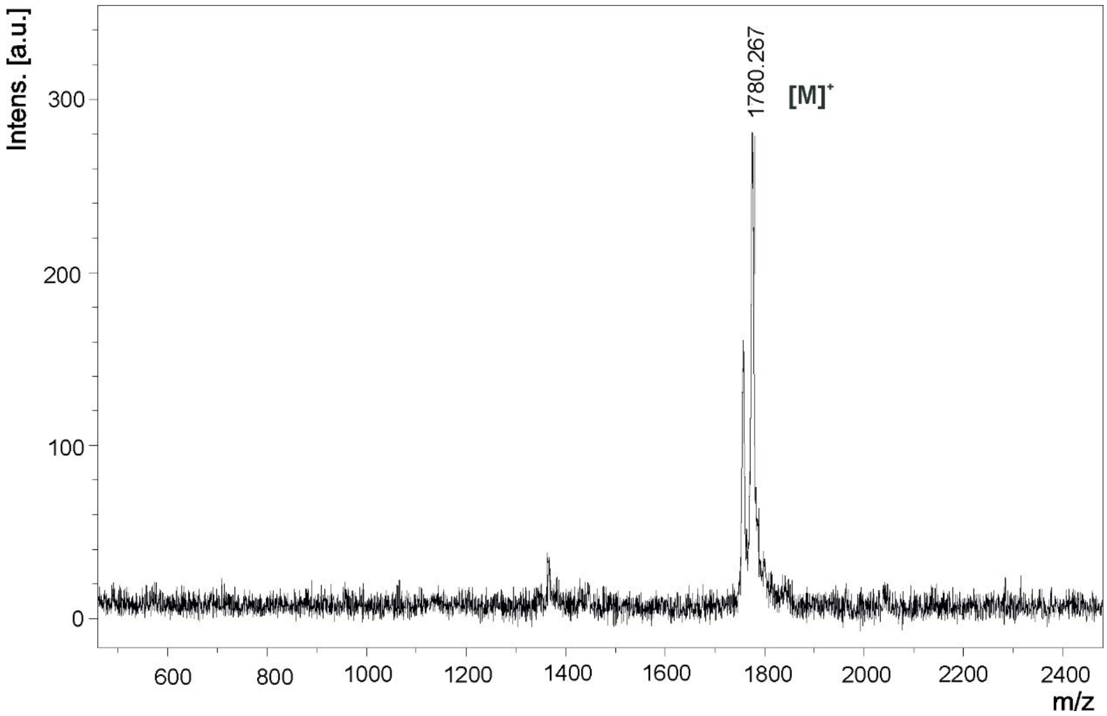

Figure S21. MALDI-MS spectrum of compound **11**

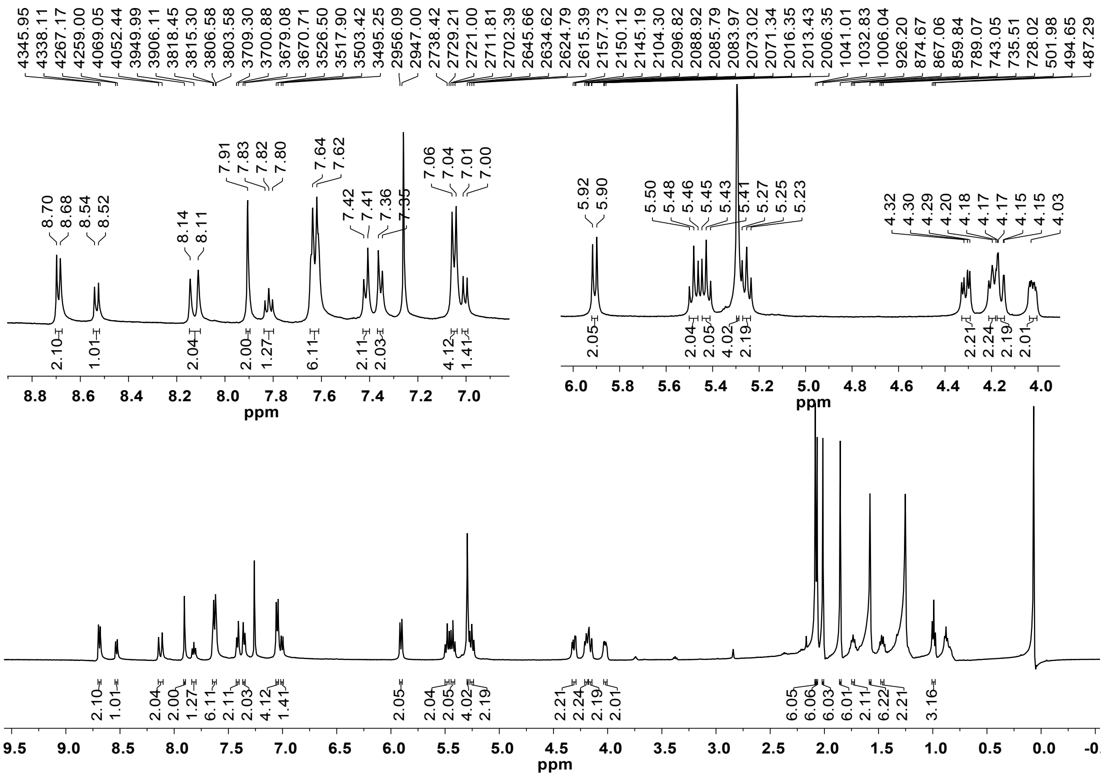

Figure S22. ^{1}H NMR spectrum of compound **11** in $CDCl_3$

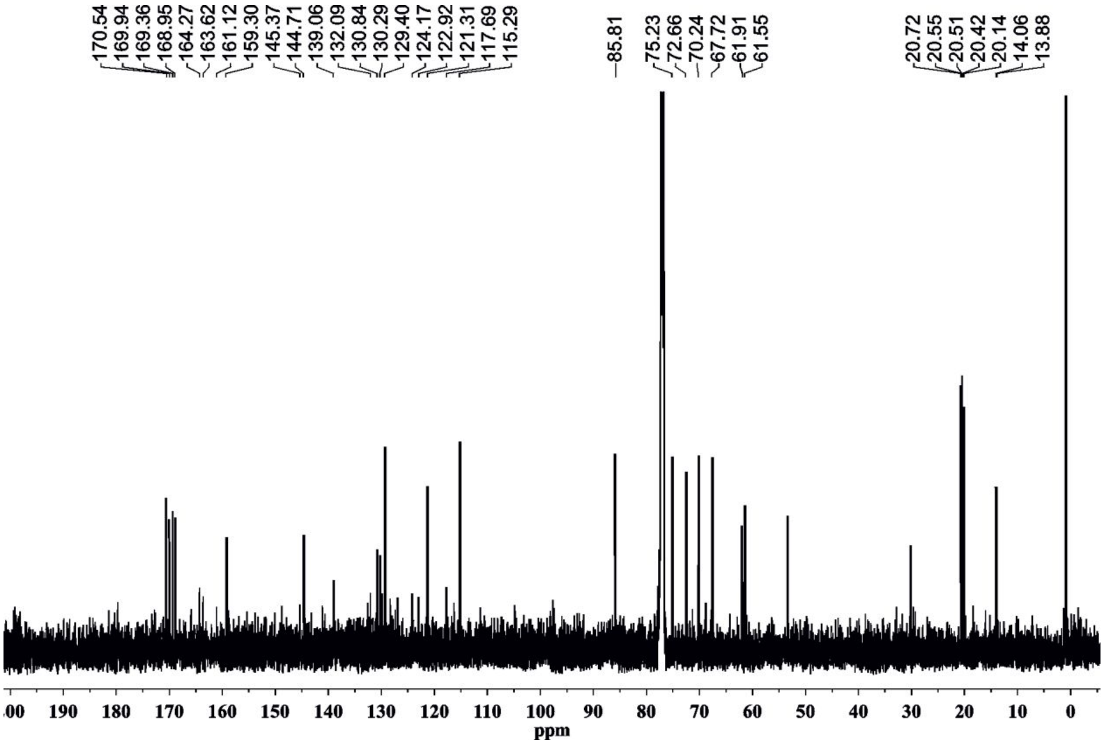

Figure S23. ^{13}C NMR spectrum of compound **11** in $CDCl_3$

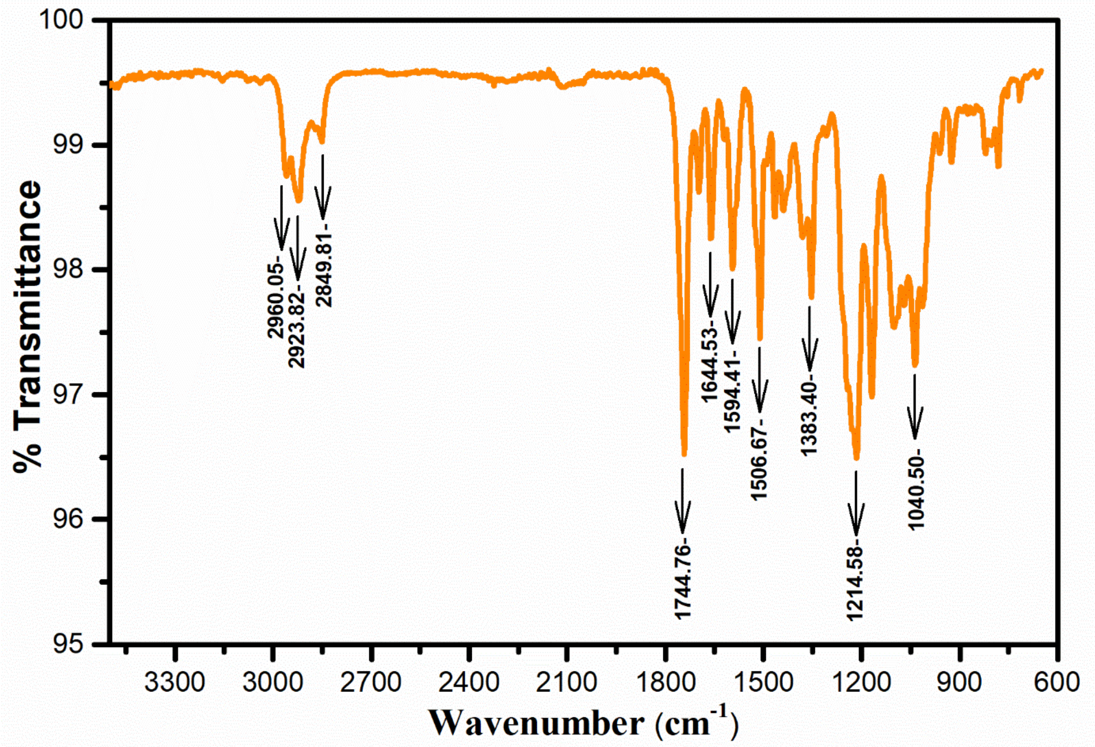

Figure S24. FT-IR spectrum of compound **12**

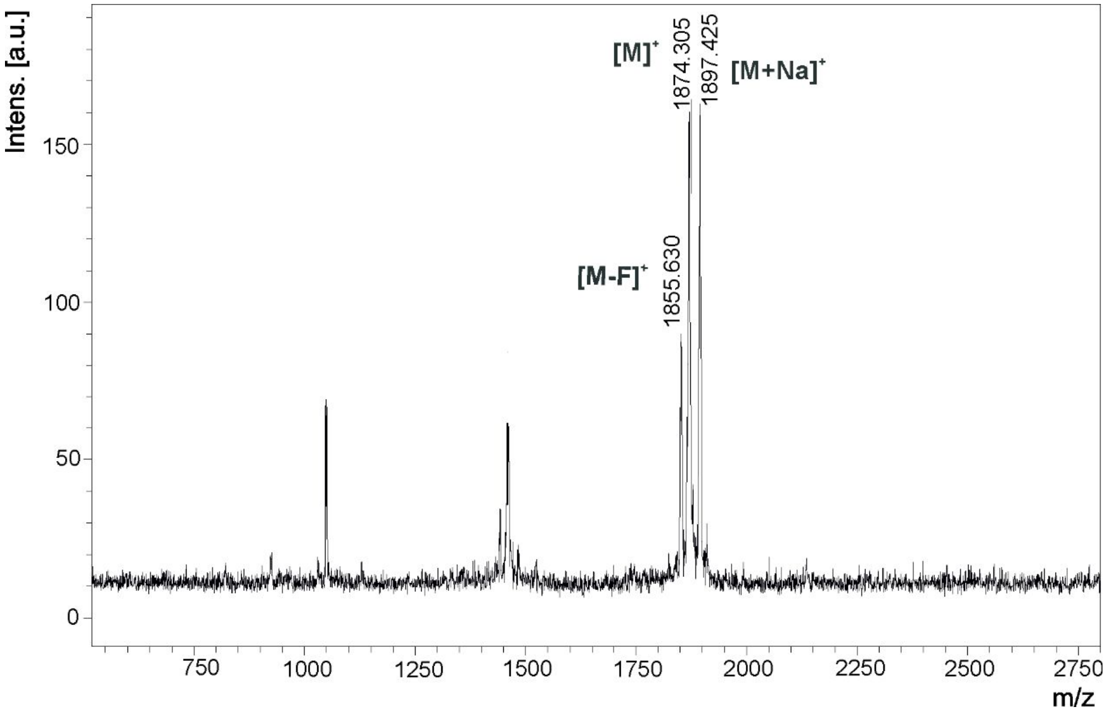

Figure S25. MALDI-MS spectrum of compound **12**

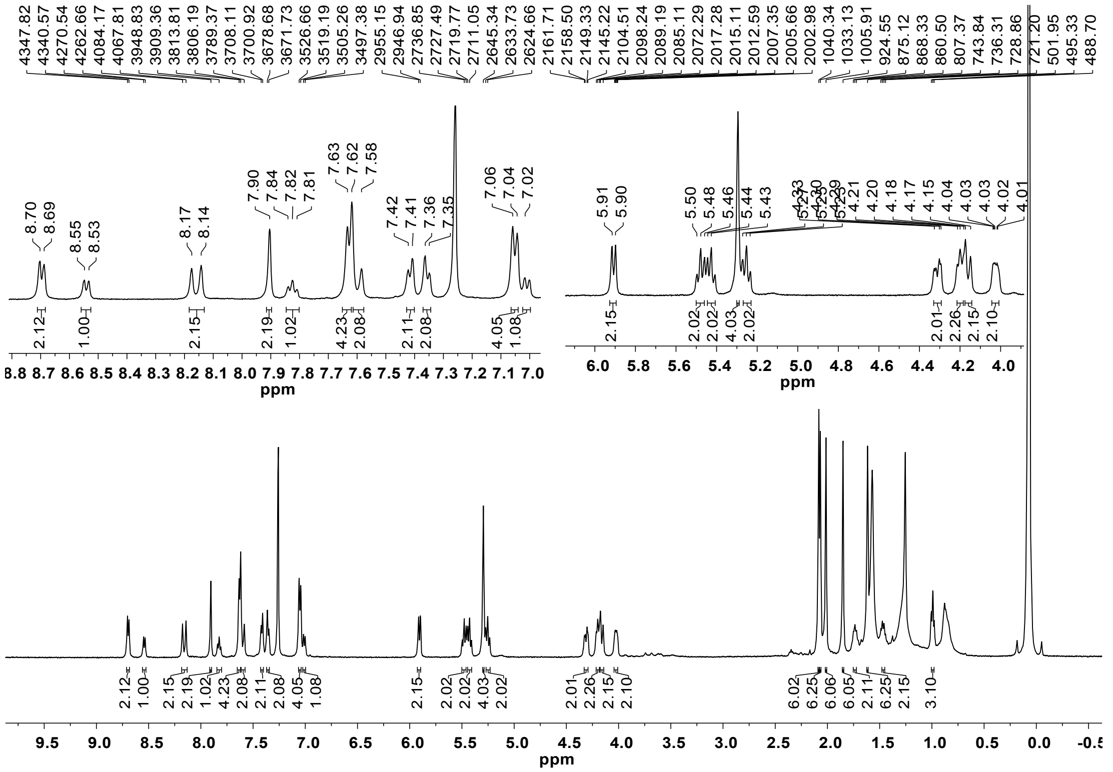

Figure S26. ^{1}H NMR spectrum of compound **12** in $CDCl_3$

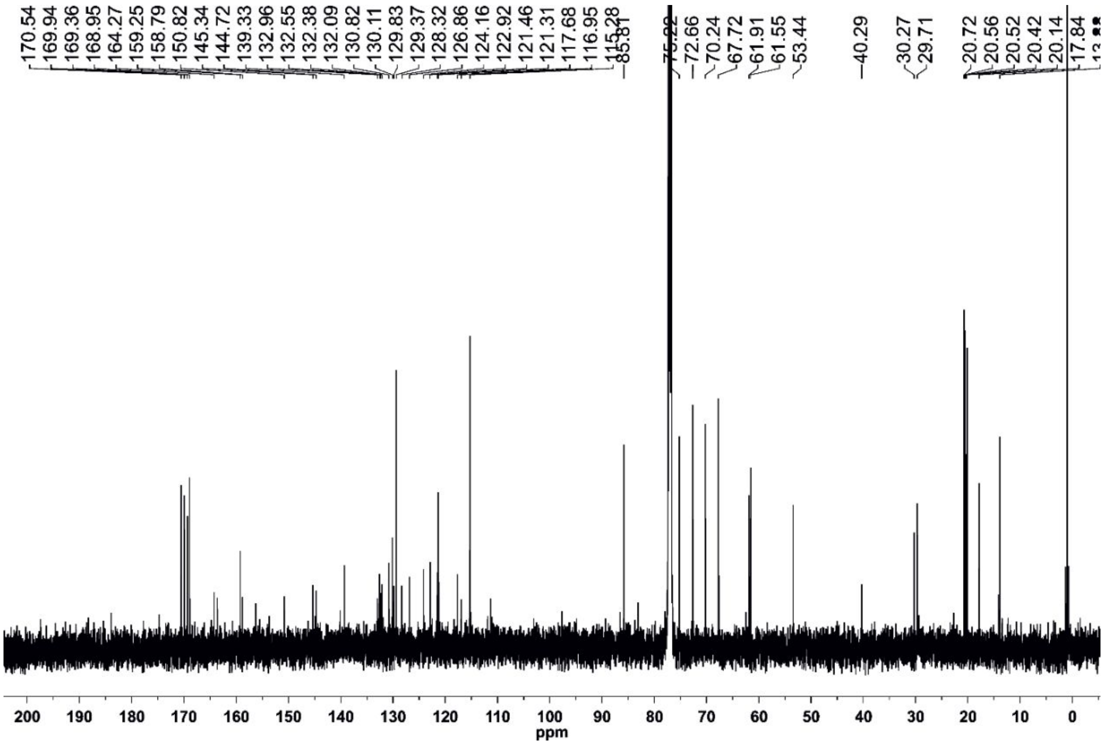

Figure S27. ^{13}C NMR spectrum of compound **12** in $CDCl_3$

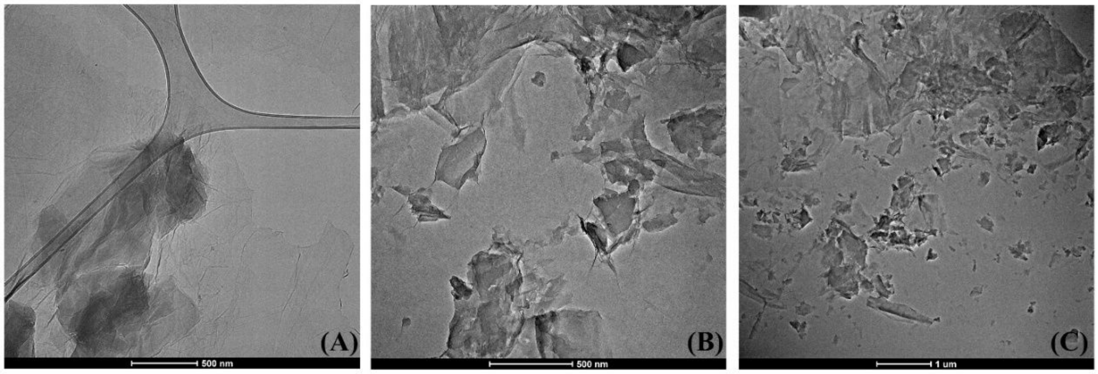

Figure S28. HR-TEM micrographs of (**A**) **GO** and (**B**, **C**) **GO-10**

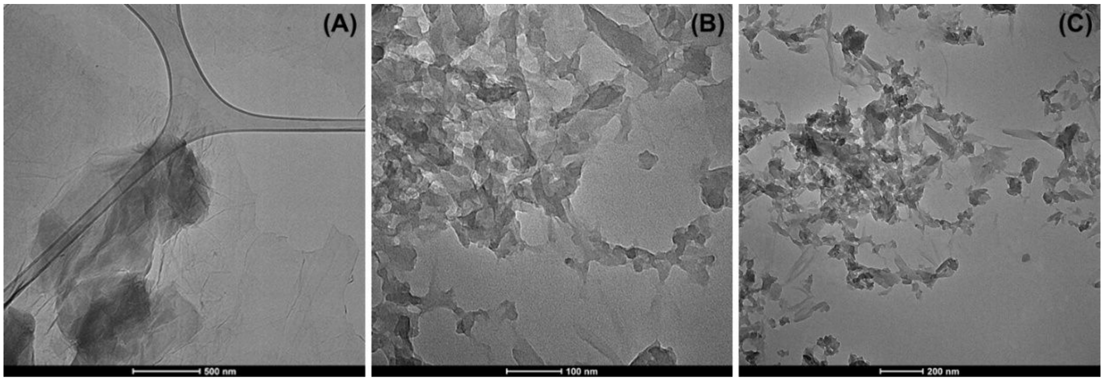

Figure S29. HR-TEM micrographs of (**A**) **GO** and (**B**, **C**) **GO-11**

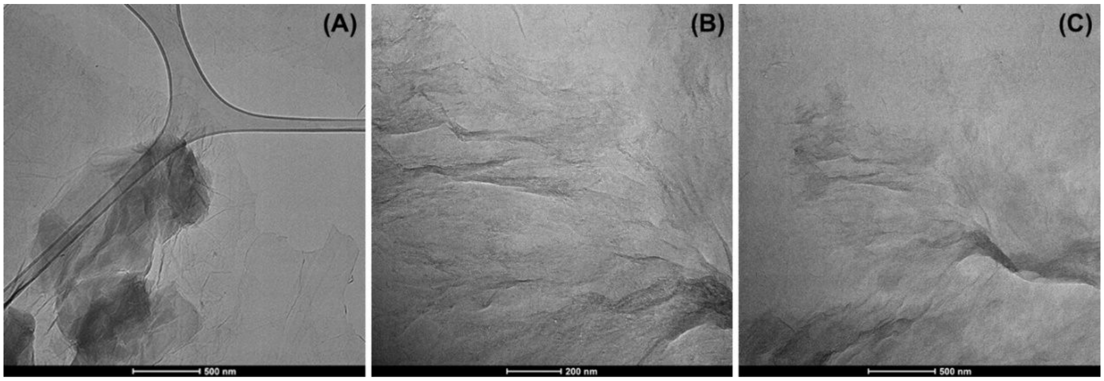

Figure S30. HR-TEM micrographs of (**A**) **GO** and (**B**, **C**) **GO-12**

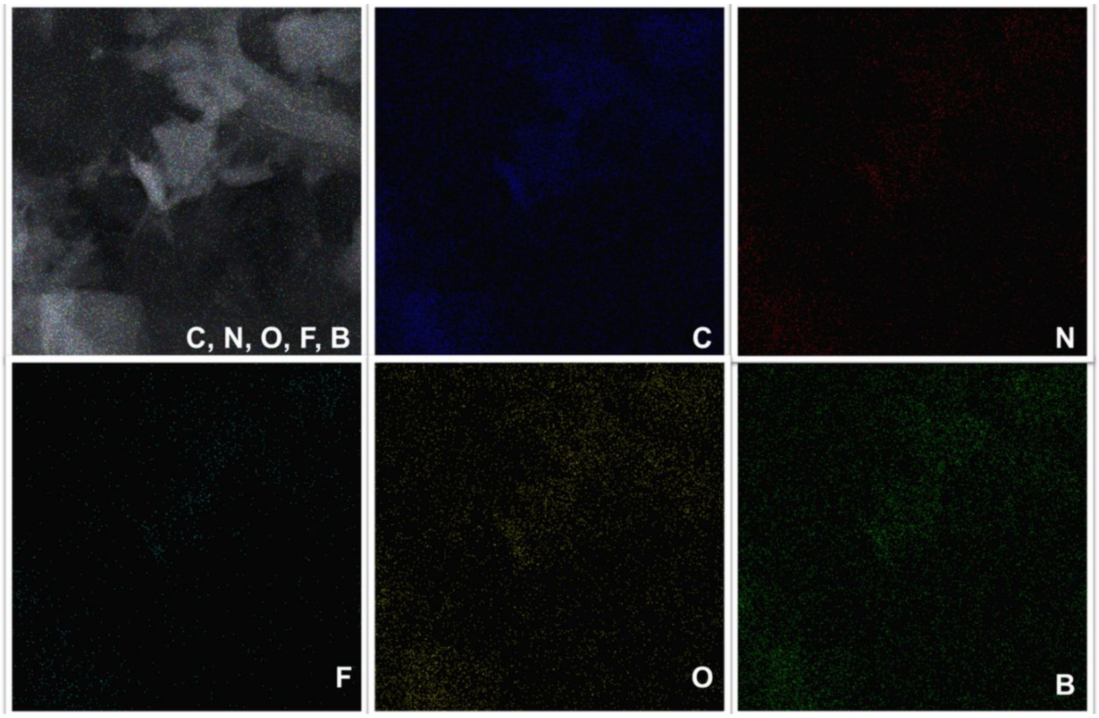

Figure S31. EDX analysis of **GO-10**

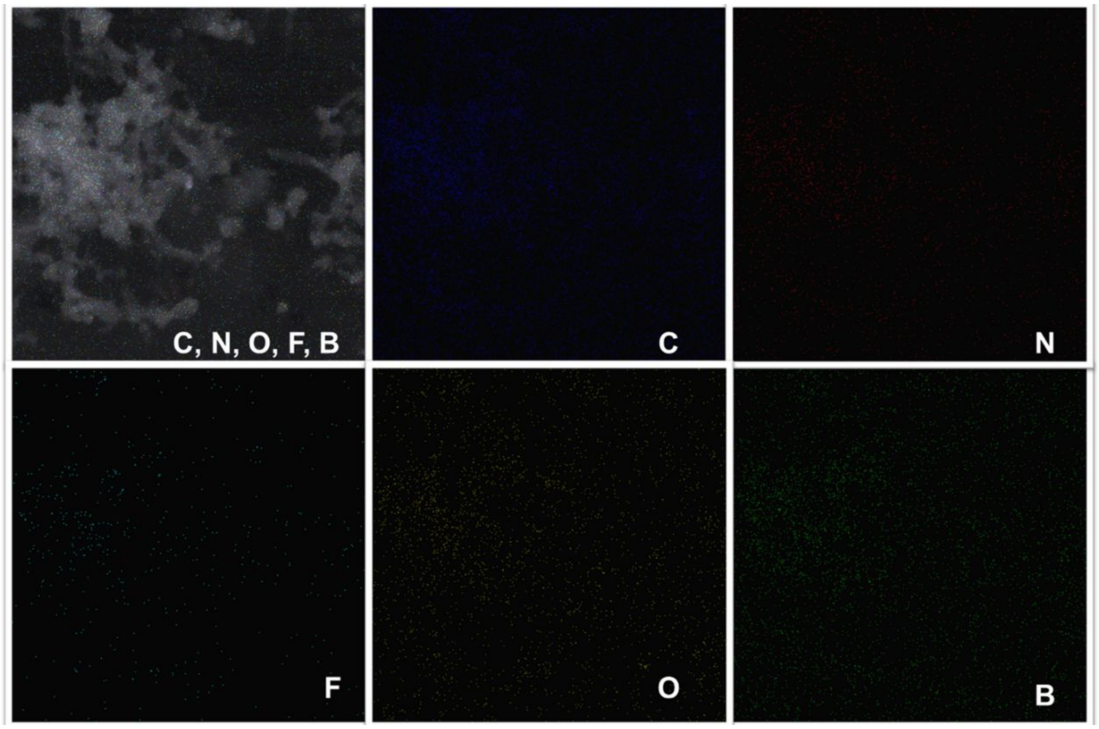

Figure S32. EDX analysis of **GO-11**

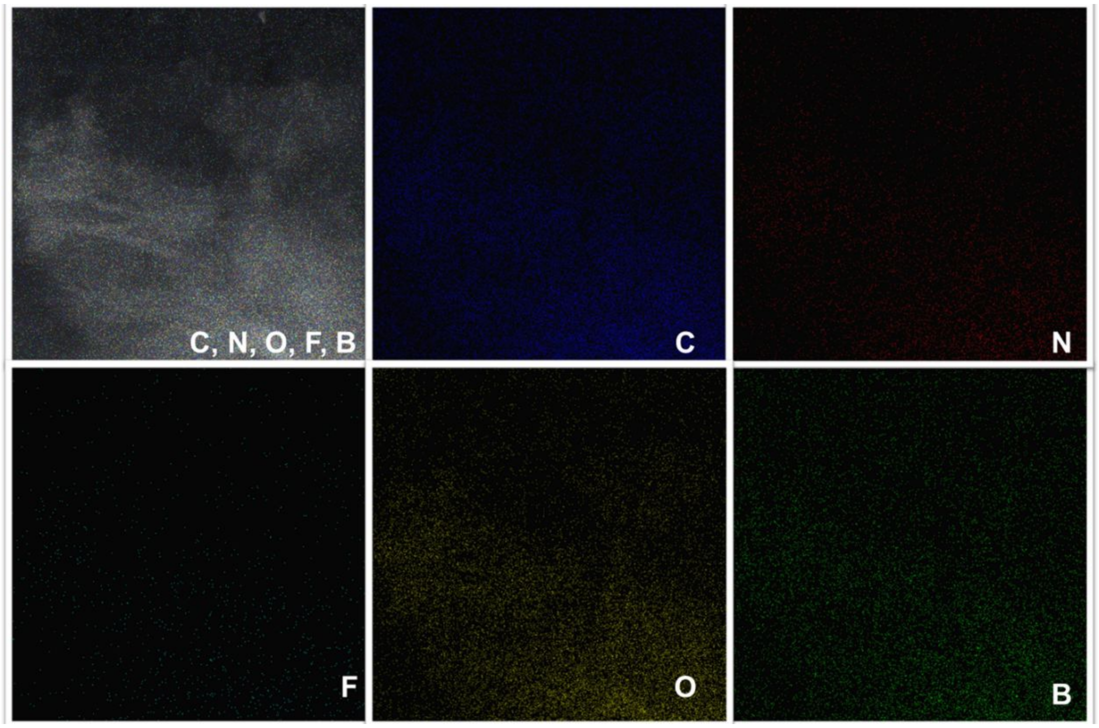

Figure S33. EDX analysis of **GO-12**

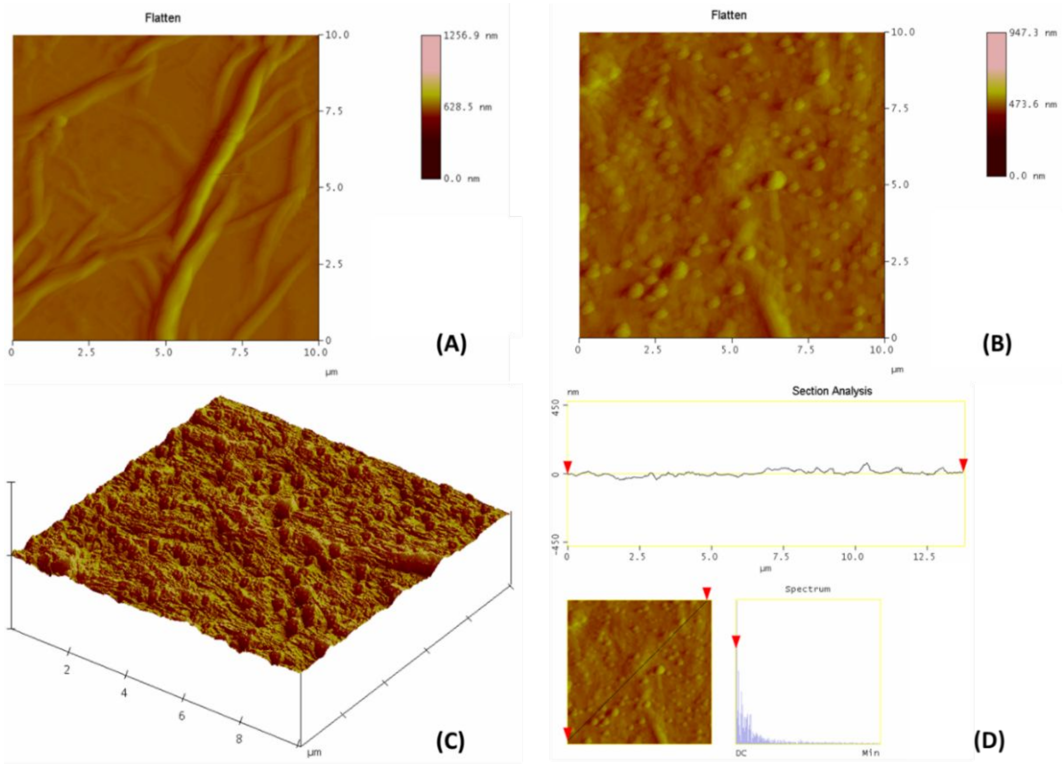

Figure S34. AFM images of (**A**) **GO** and (**B**-**D**) **GO-10**

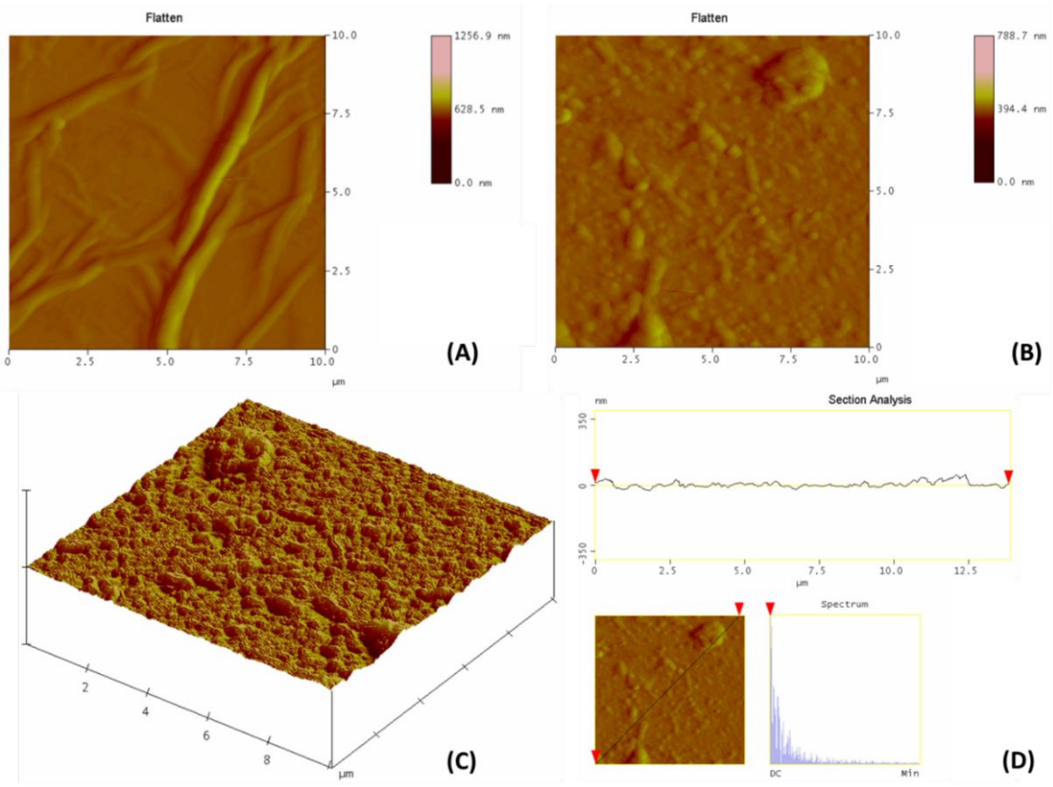

Figure S35. AFM images of (**A**) **GO** and (**B**-**D**) **GO-11**

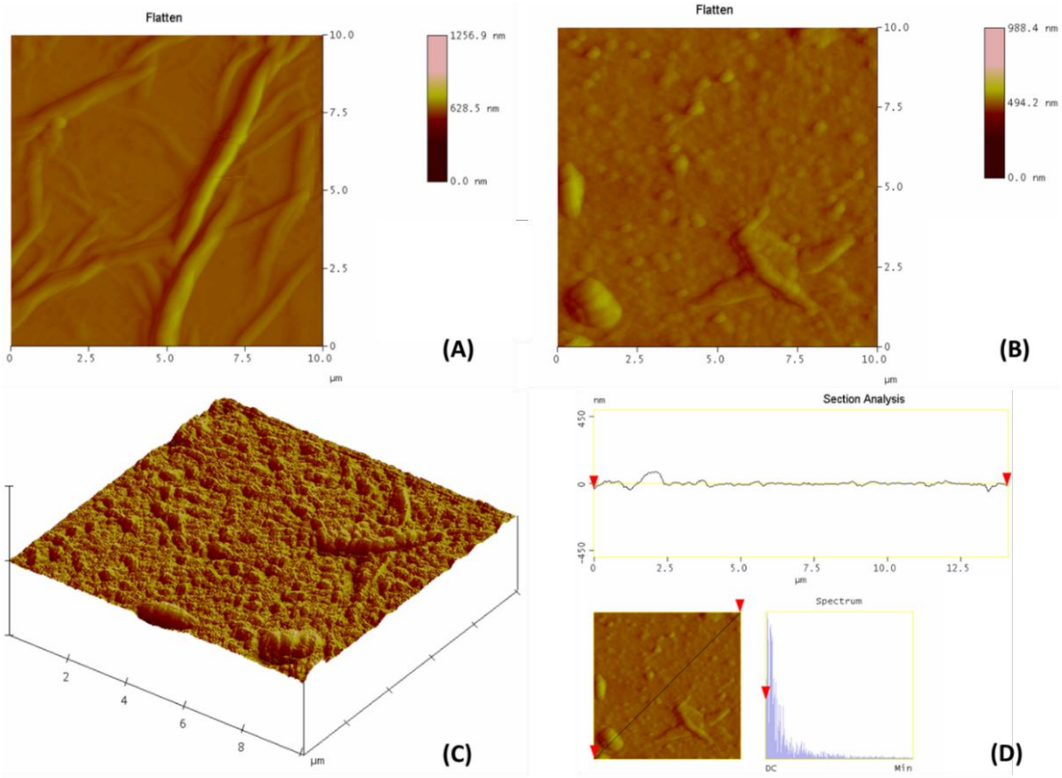

Figure S36. AFM images of (**A**) **GO** and (**B**-**D**) **GO-12**

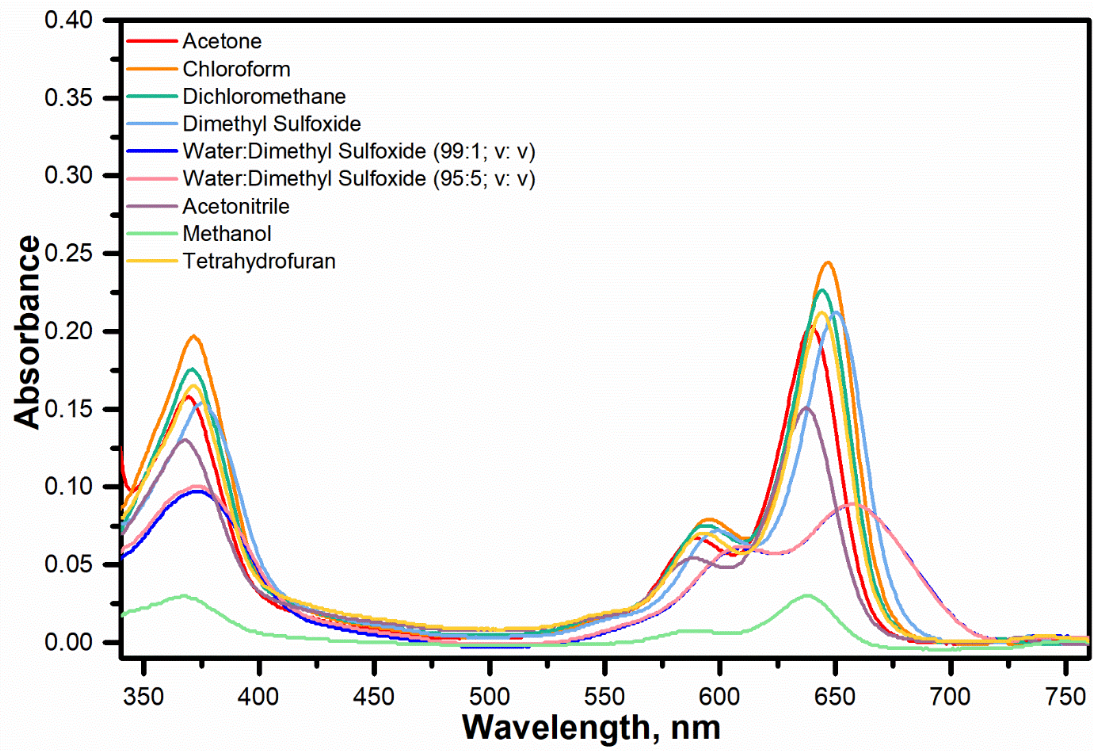

Figure S37. Absorbance spectra of compound **10** in different solvents (2 μM)

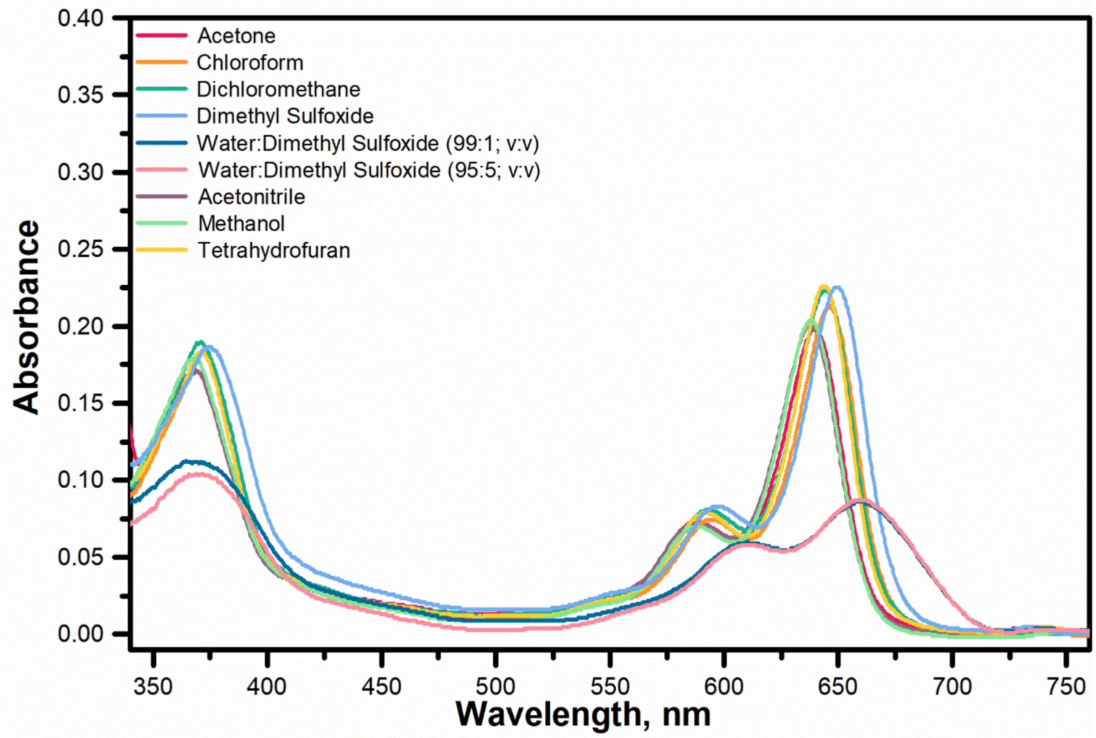

Figure S38. Absorbance spectra of **GO-10** in different solvents (2 µM)

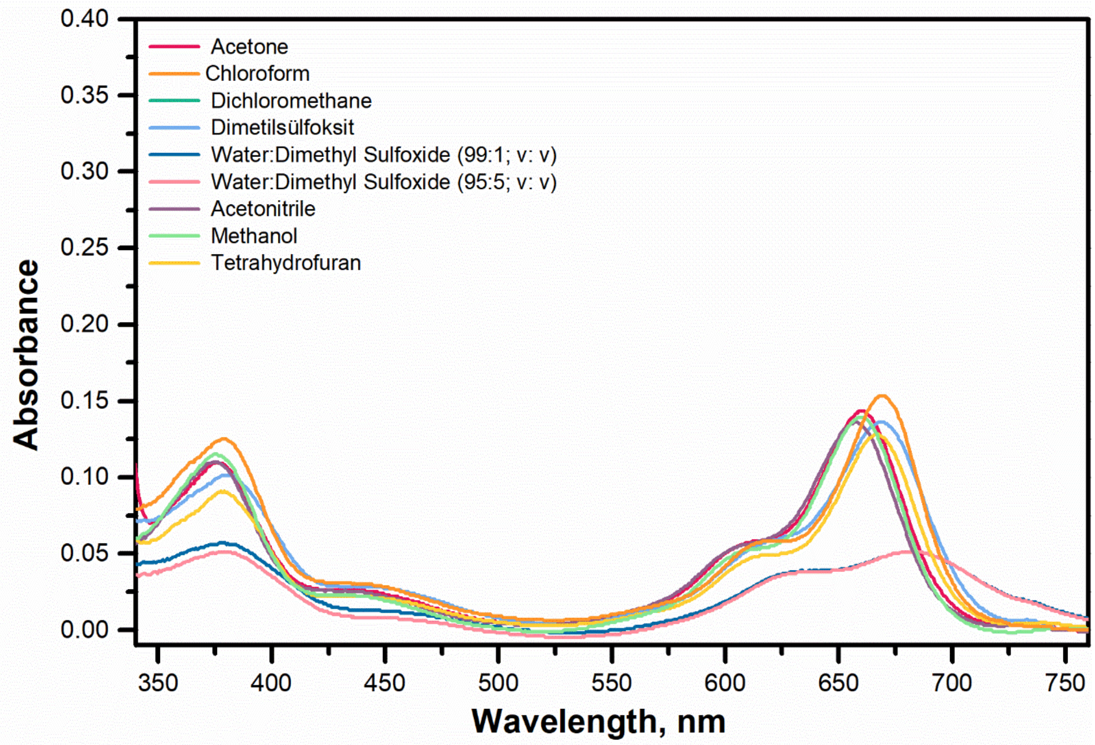

Figure S39. Absorbance spectra of compound **11** in different solvents (2 µM)

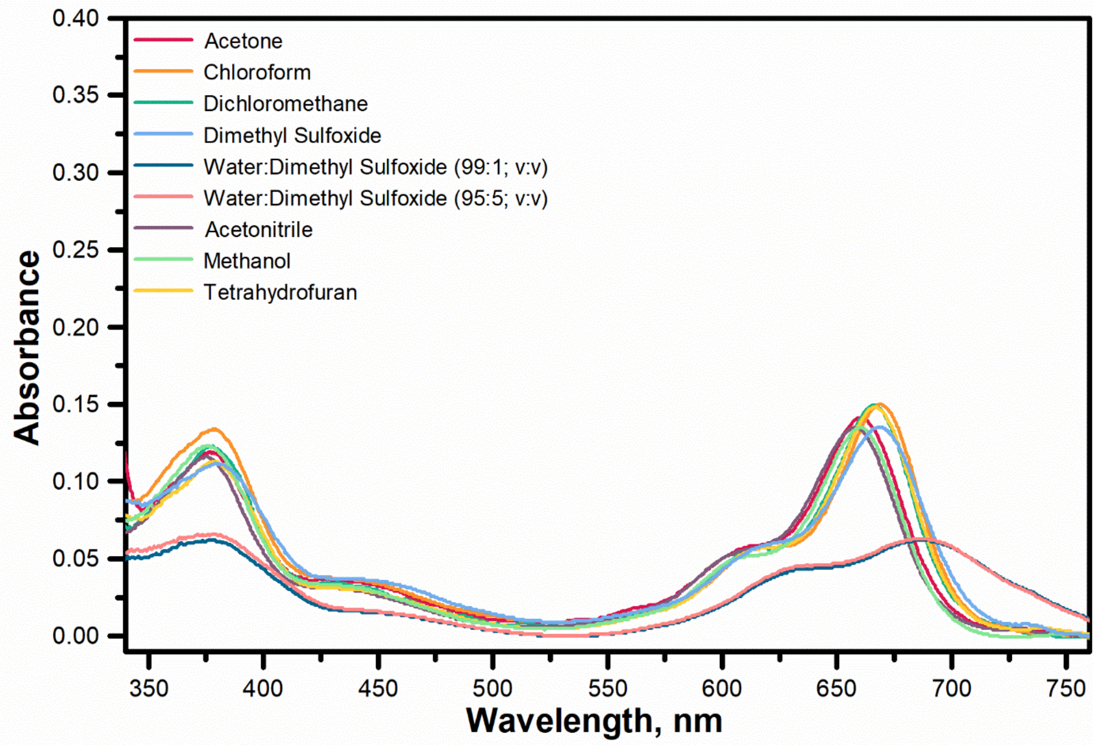

Figure S40. Absorbance spectra of **GO-11** in different solvents (2 μM)

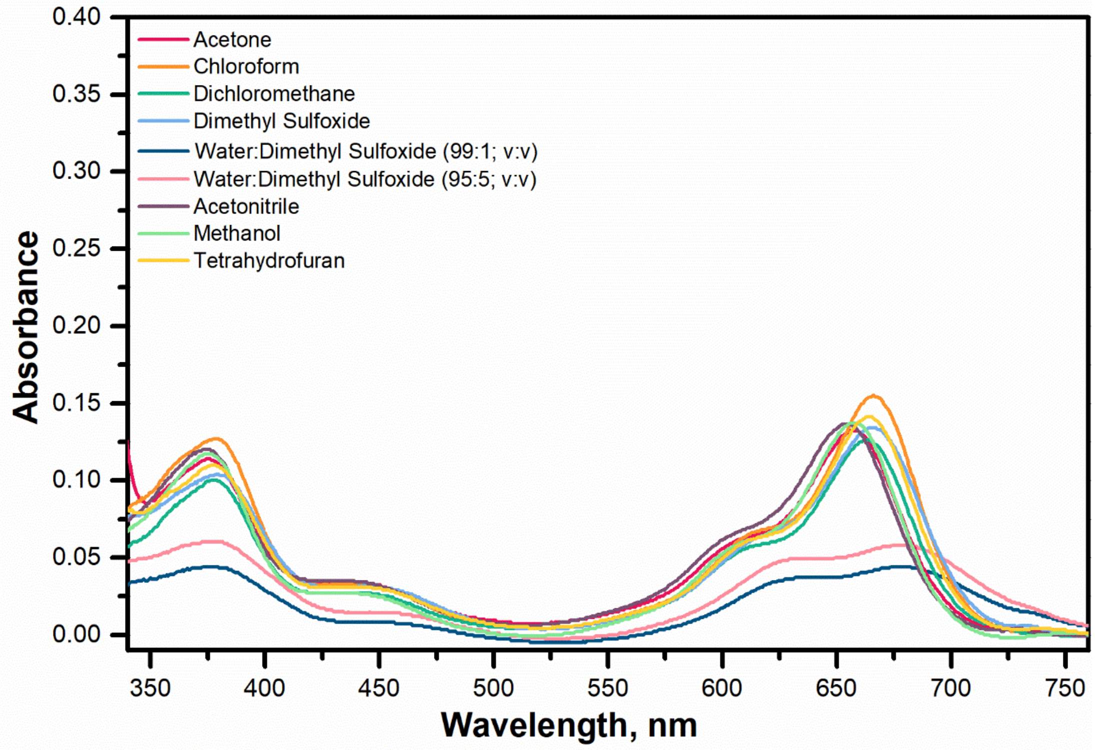

Figure S41. Absorbance spectra of compound **12** in different solvents (2 μM)

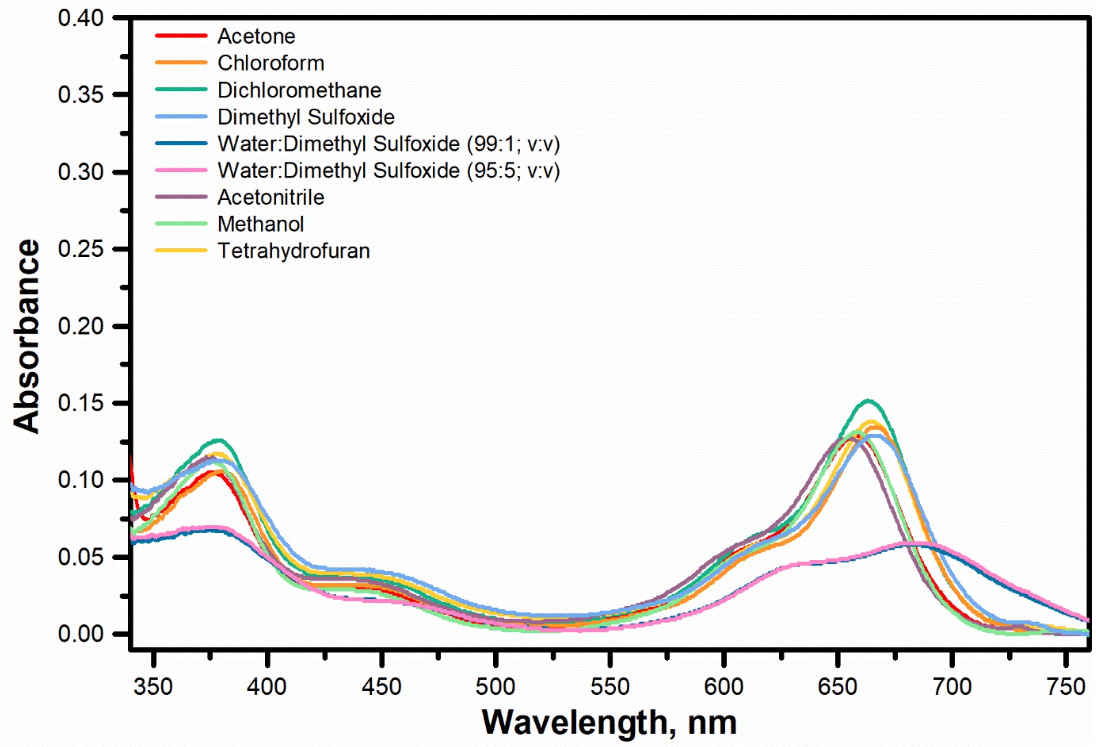

Figure S42. Absorbance spectra of **GO-12** in different solvents (2 μM)

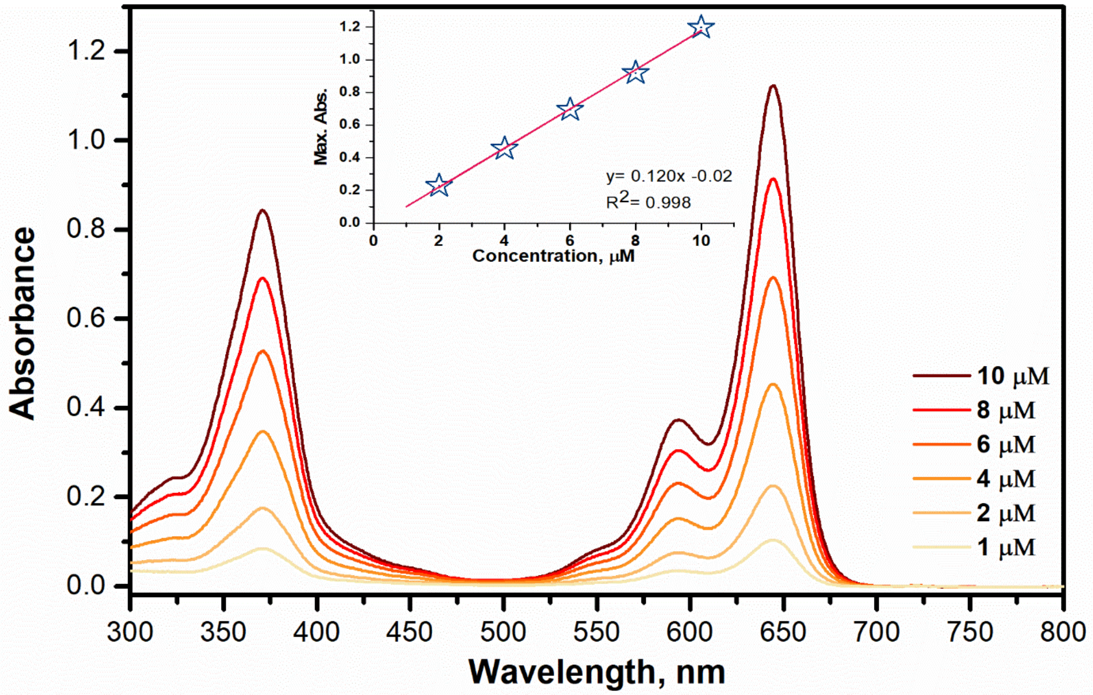

Figure S43. Absorption spectra of compound **10** in DCM at different concentrations

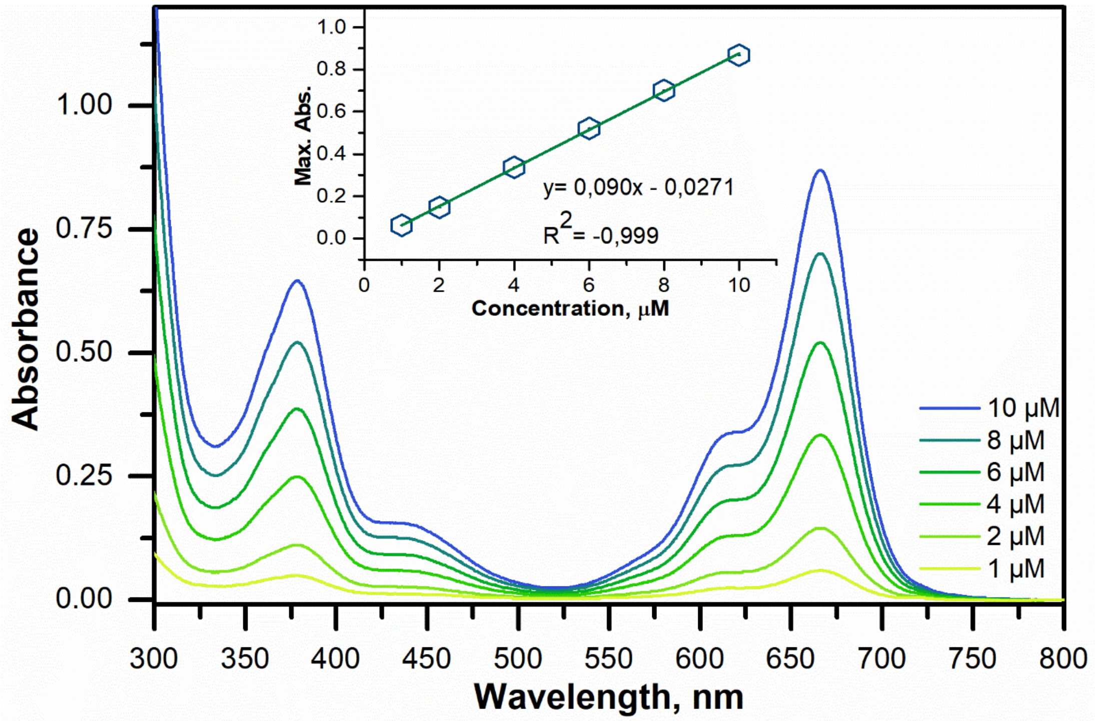

Figure S44. Absorption spectra of compound **11** in DCM at different concentrations

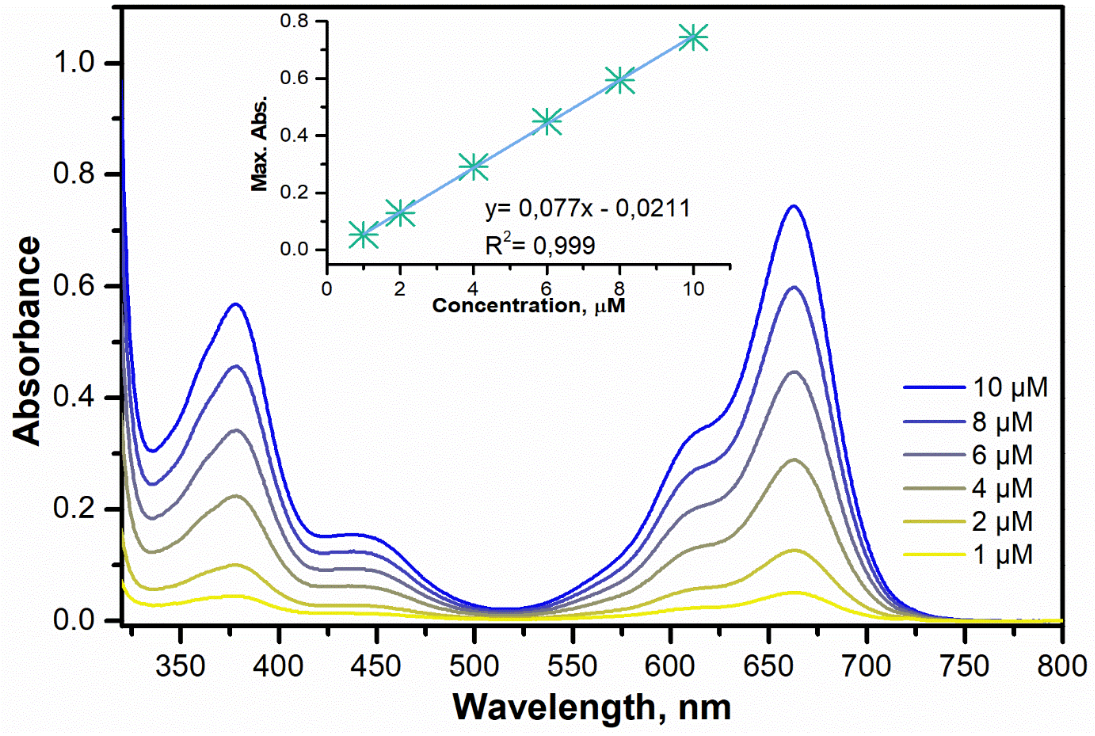

Figure S45. Absorption spectra of compound **12** in DCM at different concentrations

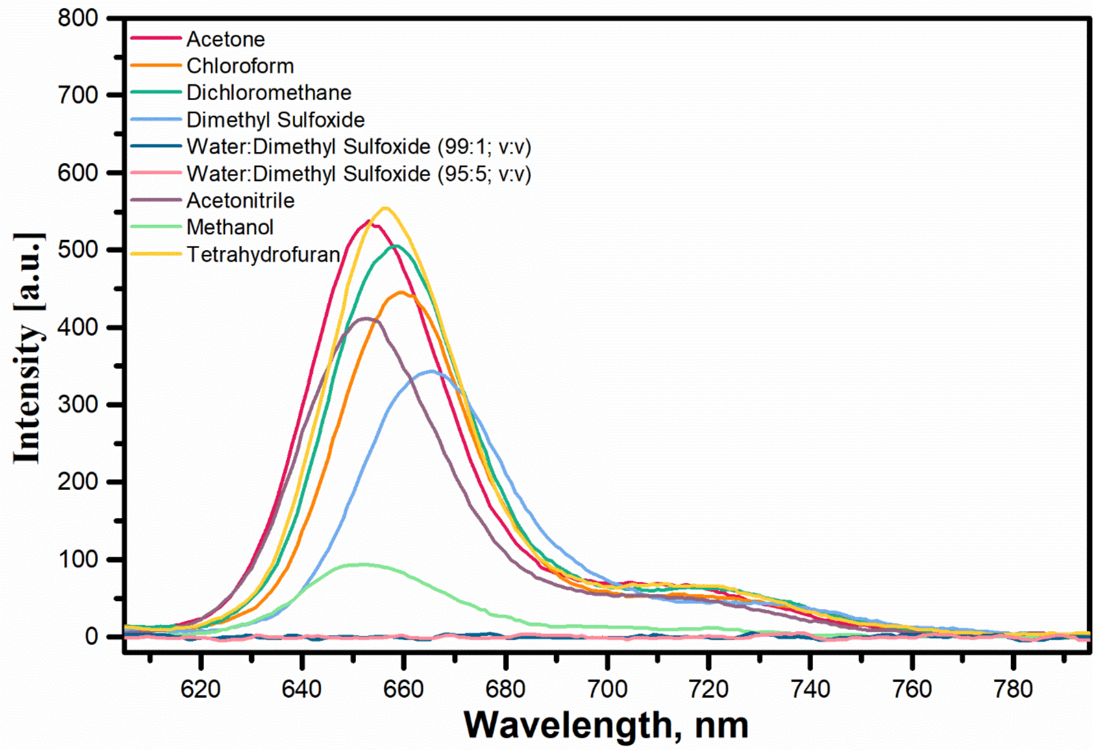

Figure S46. Fluorescence spectra of compound **10** (λ_{ex}:590 nm) in different solvents (0.2 µM)

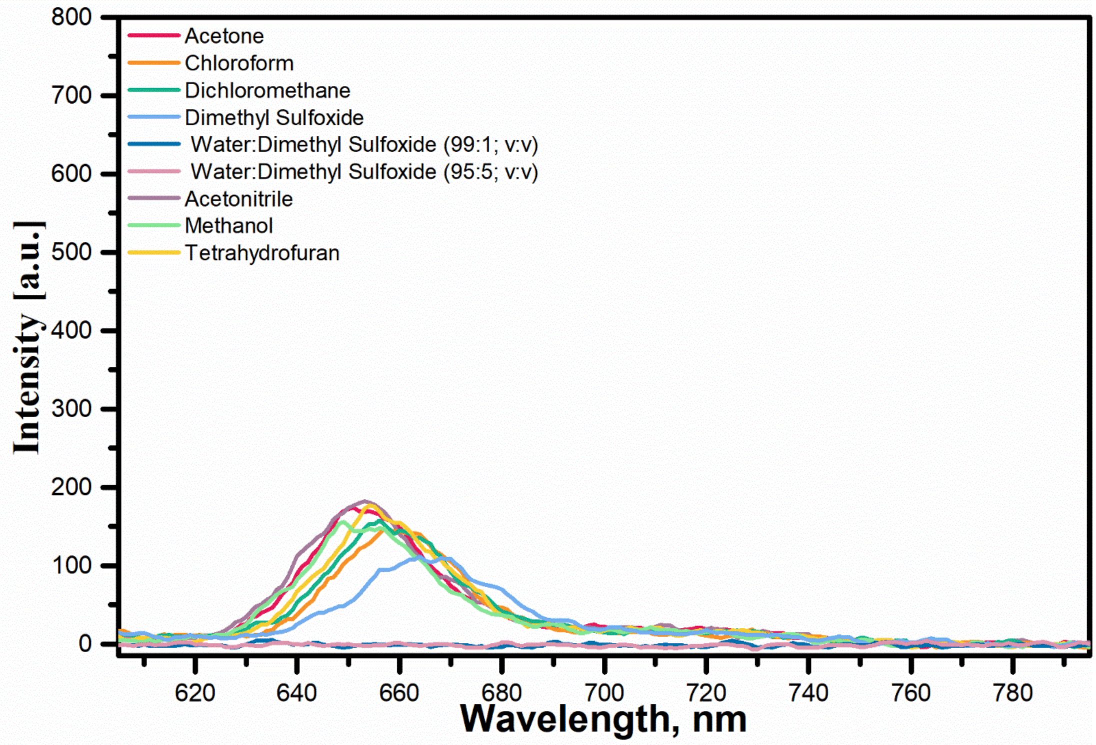

Figure S47. Fluorescence spectra of **GO-10** (λ_{ex}:590 nm) in different solvents (0.2 µM)

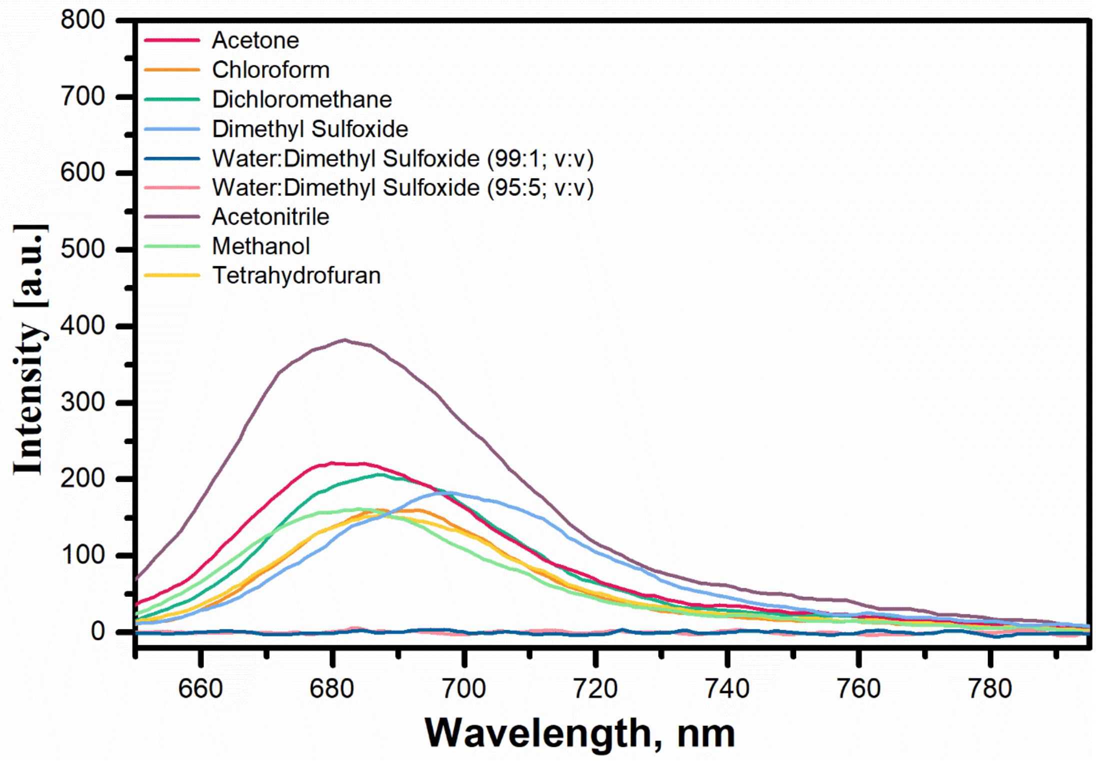

Figure S48. Fluorescence spectra of compound **11** (λ_{ex}:635 nm) in different solvents (0.2 µM)

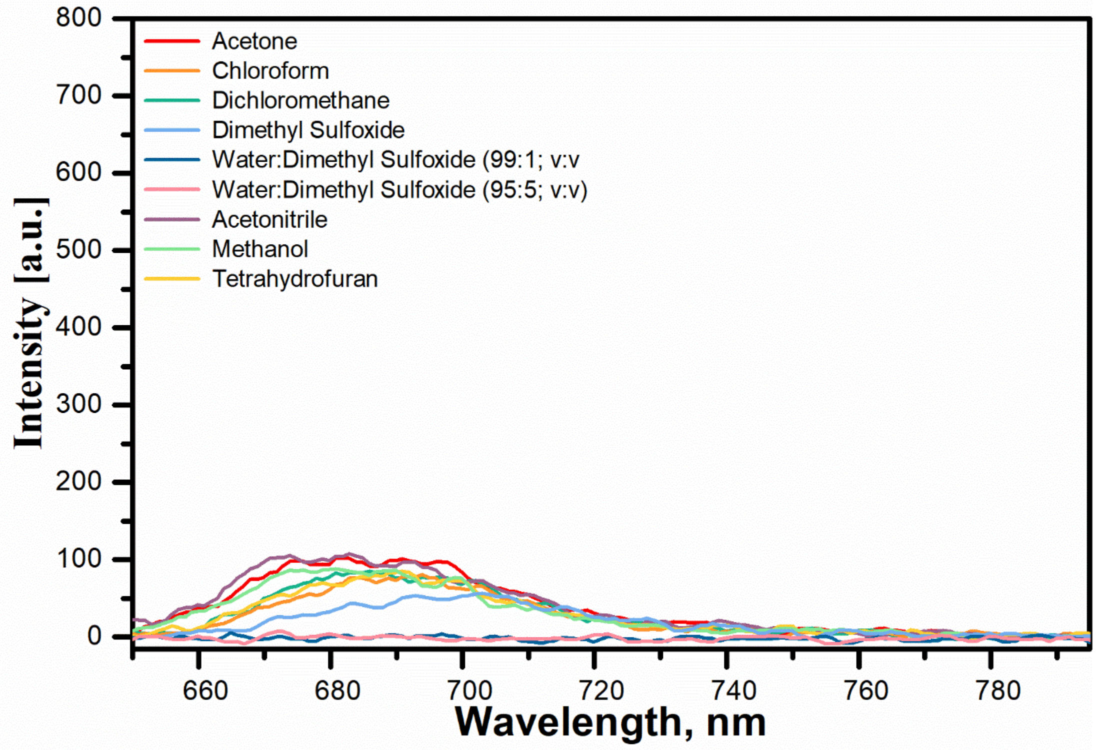

Figure S49. Fluorescence spectra of **GO-11** (λex:635 nm) in different solvents (0.2 µM)

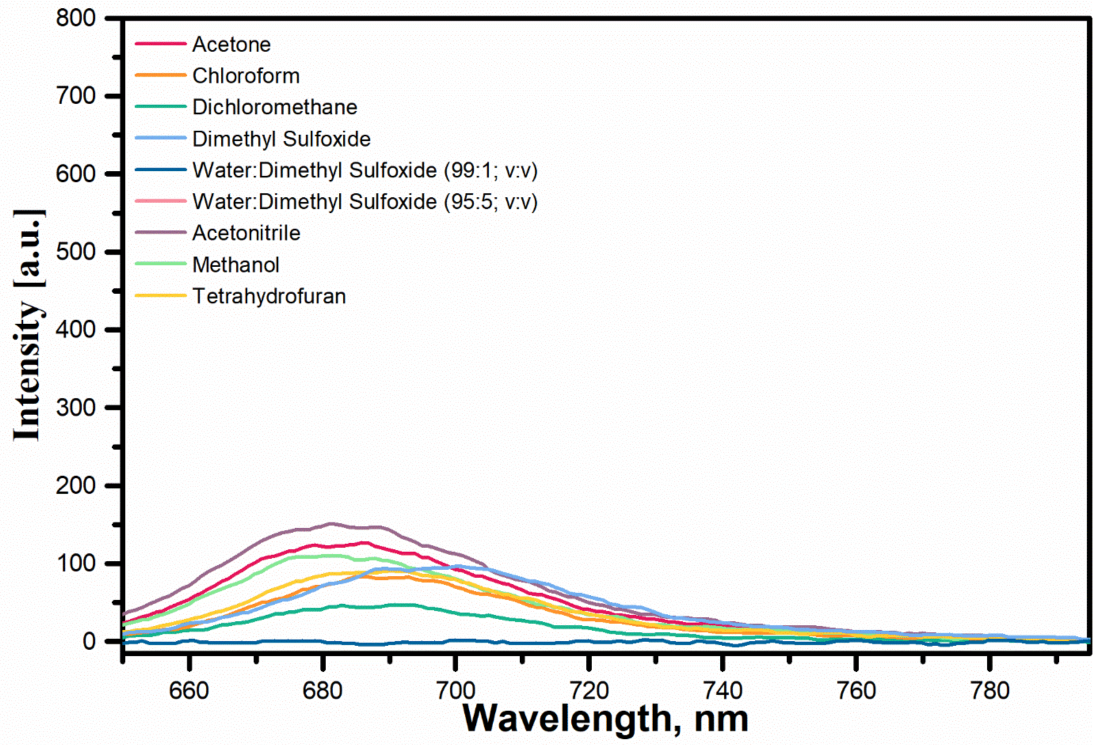

Figure S50. Fluorescence spectra of compound **12** (λ_{ex}:635 nm) in different solvents (0.2 μM)

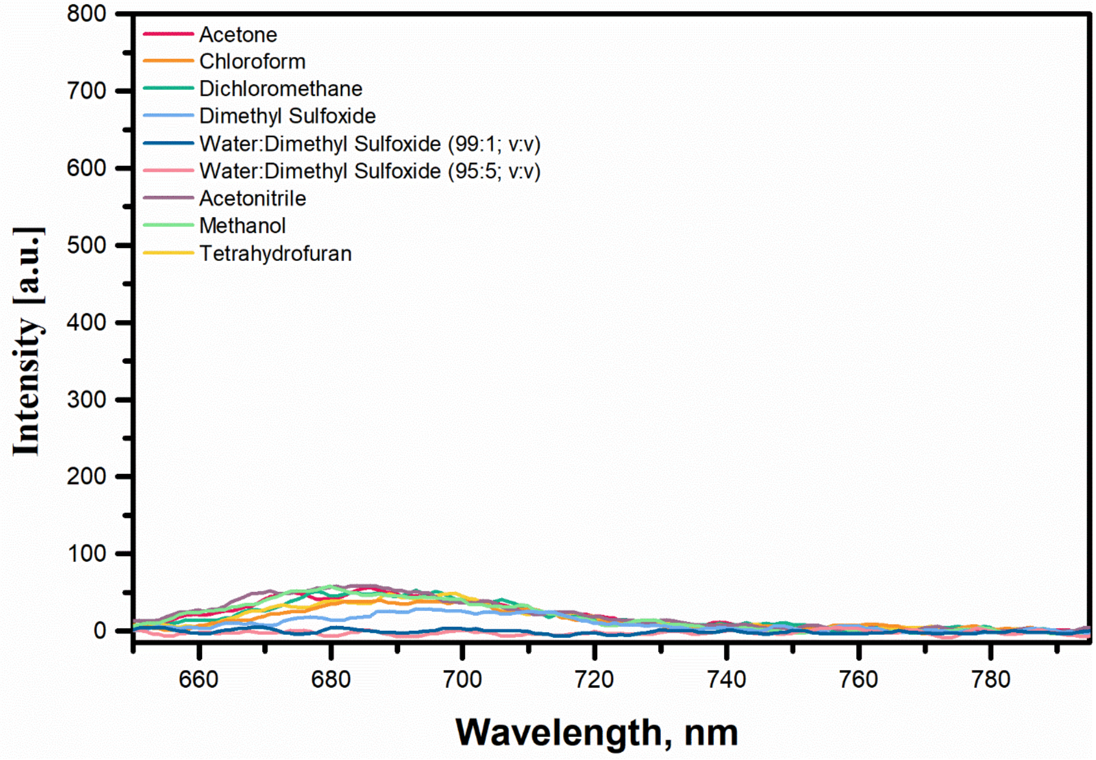

Figure S51. Fluorescence spectra of **GO-12** (λex:635 nm) in different solvents (0.2 μM)

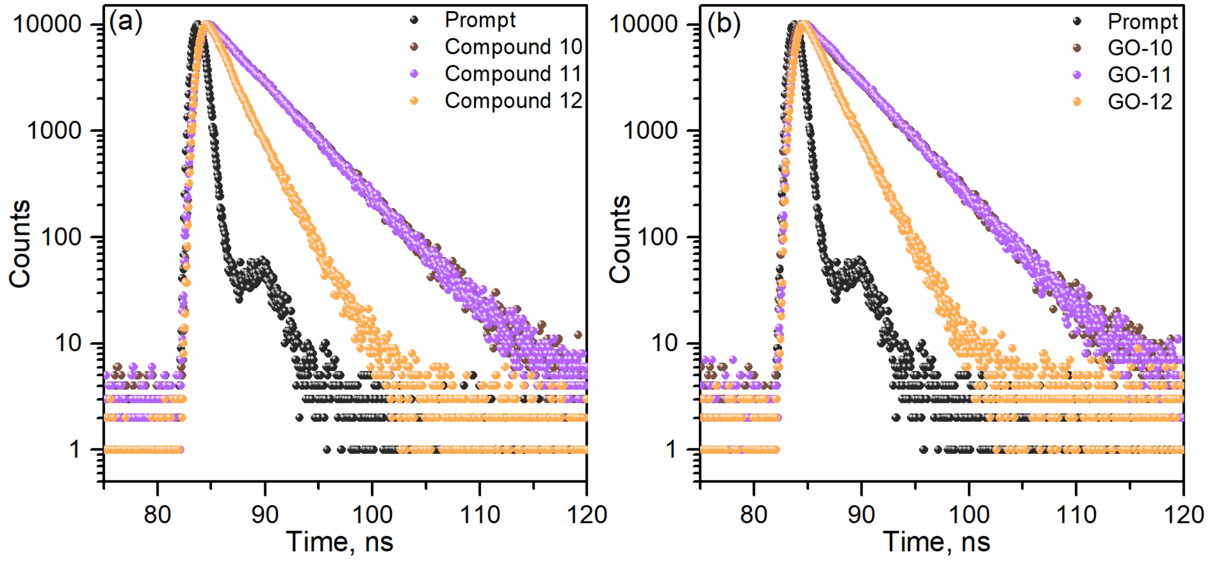

Figure S52. Fluorescence decay profiles of **10-12** and **GO-(10-12)** in DCM

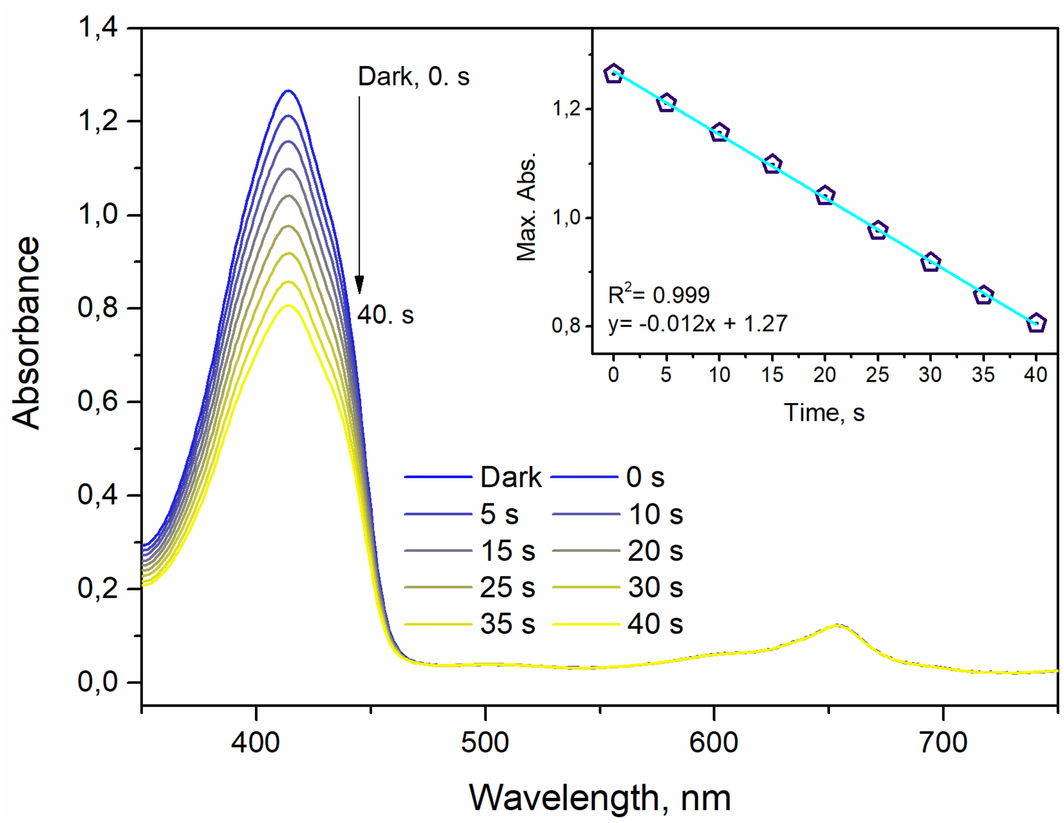

Figure S53. Decrease in absorbance spectra of DPBF in the presence of MB in DCM

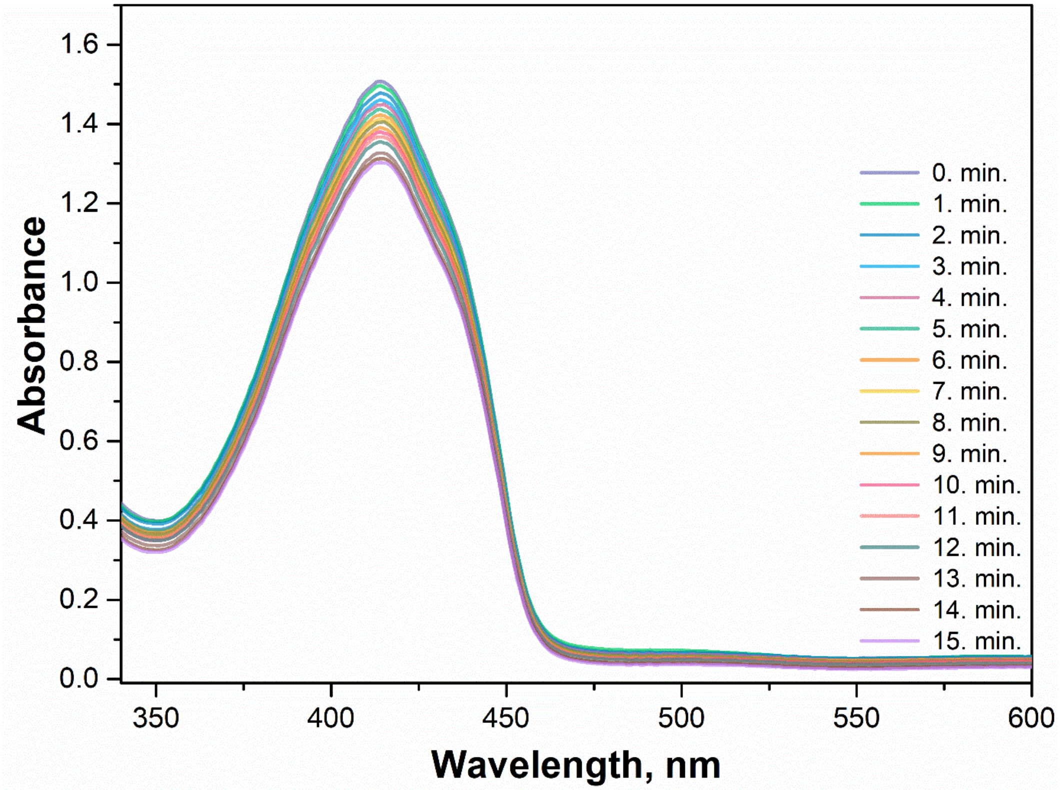

Figure S54. Decrease in absorbance spectra of DPBF in the presence of GO in DCM

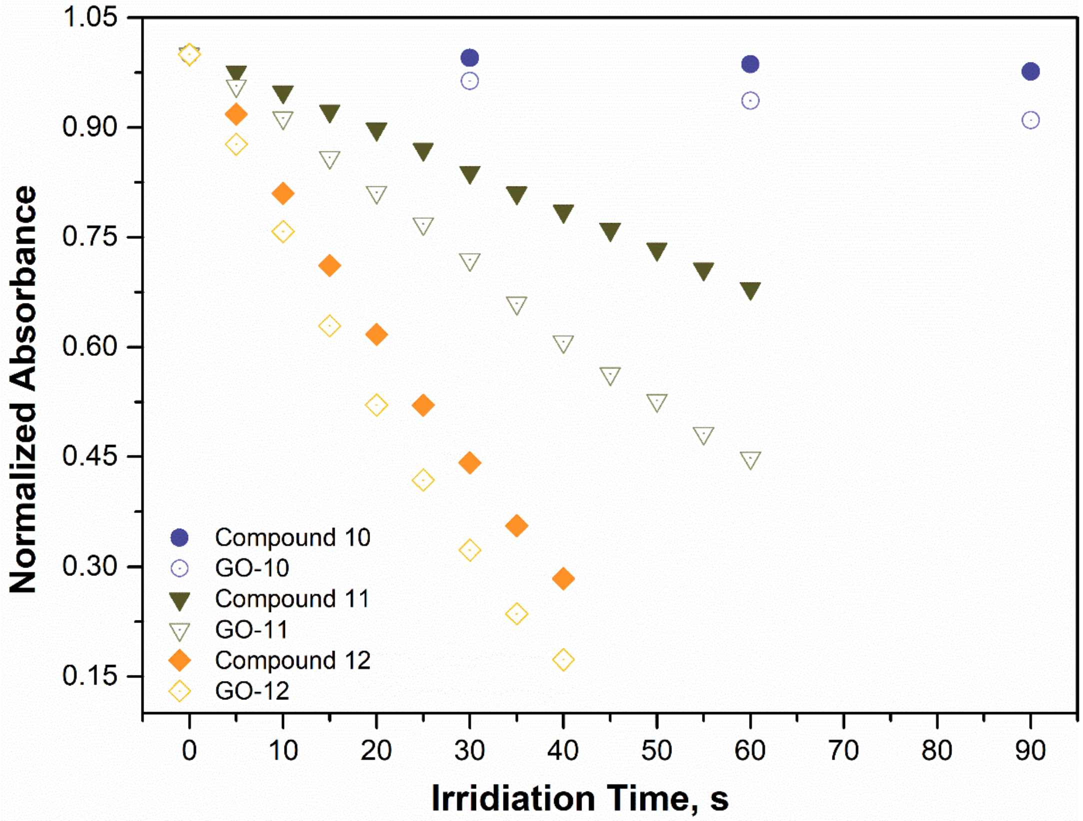

Figure S55. Decrease in maximum absorbances of DPBF after irradiation

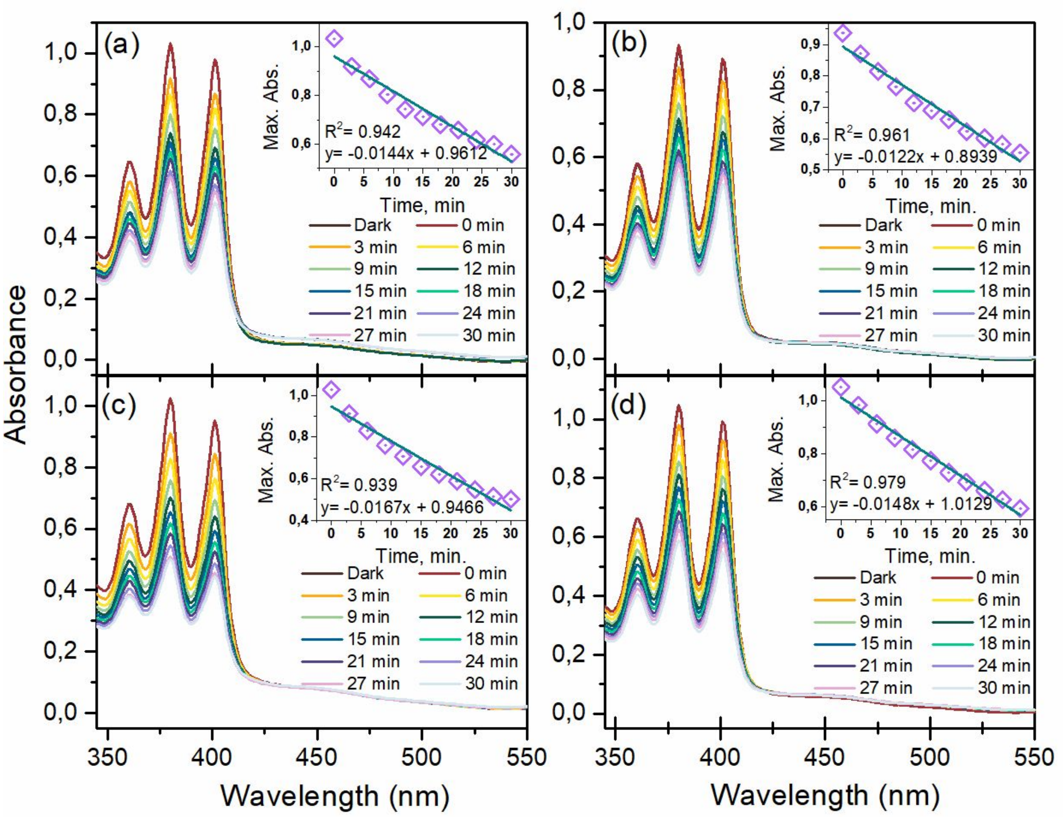

Figure S56. Decrease in absorbance spectra of ABDA in the presence of (a) **11**, (b) **12**, (c) **GO-11** and (d) **GO-12**
